# Molecular Characterization of the *Elaeis guineensis* Medium-Chain Fatty Acid Diacylglycerol Acyltransferase DGAT1-1 by Heterologous Expression in *Yarrowia lipolytica*

**DOI:** 10.1371/journal.pone.0143113

**Published:** 2015-11-18

**Authors:** Laure Aymé, Pascale Jolivet, Jean-Marc Nicaud, Thierry Chardot

**Affiliations:** 1 INRA, UMR1318, Institut Jean-Pierre Bourgin Saclay Plant Sciences, Versailles, France; 2 AgroParisTech, UMR1318, Institut Jean-Pierre Bourgin, Versailles, France; 3 Micalis, UMR1319 INRA-AgroParisTech, Jouy-en-Josas, France; Huazhong university of Science and Technology, CHINA

## Abstract

Diacylglycerol acyltransferases (DGAT) are involved in the acylation of *sn*-1,2-diacylglycerol. Palm kernel oil, extracted from *Elaeis guineensis* (oil palm) seeds, has a high content of medium-chain fatty acids mainly lauric acid (C12:0). A putative *E*. *guineensis* diacylglycerol acyltransferase gene (*EgDGAT1-1*) is expressed at the onset of lauric acid accumulation in the seed endosperm suggesting that it is a determinant of medium-chain triacylglycerol storage. To test this hypothesis, we thoroughly characterized EgDGAT1-1 activity through functional complementation of a *Yarrowia lipolytica* mutant strain devoid of neutral lipids. EgDGAT1-1 expression is sufficient to restore triacylglycerol accumulation in neosynthesized lipid droplets. A comparative functional study with *Arabidopsis thaliana* DGAT1 highlighted contrasting substrate specificities when the recombinant yeast was cultured in lauric acid supplemented medium. The EgDGAT1-1 expressing strain preferentially accumulated medium-chain triacylglycerols whereas AtDGAT1 expression induced long-chain triacylglycerol storage in *Y*. *lipolytica*. EgDGAT1-1 localized to the endoplasmic reticulum where TAG biosynthesis takes place. Reestablishing neutral lipid accumulation in the *Y*. *lipolytica* mutant strain did not induce major reorganization of the yeast microsomal proteome. Overall, our findings demonstrate that EgDGAT1-1 is an endoplasmic reticulum DGAT with preference for medium-chain fatty acid substrates, in line with its physiological role in palm kernel. The characterized EgDGAT1-1 could be used to promote medium-chain triacylglycerol accumulation in microbial-produced oil for industrial chemicals and cosmetics.

## Introduction

Triacylglycerols (TAGs) are highly reduced molecules, which provide a reservoir of carbon and metabolic energy for eukaryotic cells. TAGs are stored in dedicated organelles called lipid droplets (LDs). Plant LDs are mainly found in oil accumulating seeds and fruits [1]. They are composed of a hydrophobic core of neutral lipid, TAGs and steryl esters, surrounded by a monolayer of phospholipids which contains various types of specialized proteins [2]. The Kennedy pathway is the major metabolic pathway for TAG synthesis [3–5] mainly at the endoplasmic reticulum (ER) membrane [6]. It involves acyl-CoA dependent acylations of the *sn*-1 (glycerol-3-phosphate acyltransferase, EC 2.3.1.15) and *sn*-2 (lysophosphatidic acid acyltransferase, EC 2.3.1.51) positions of glycerol-3-phosphate. After removal of the phosphate group (phosphatidate phosphatase, EC 3.1.3.4) acyl-CoA dependent diacylglycerol acyltransferase (DGAT; EC 2.3.1.20) acylates the *sn*-3 position of *sn*-1,2-diacylglycerol (DAG). DGAT catalyzes the only step exclusively committed to TAG synthesis. In yeast and plants, an acyl-CoA independent phospholipid: DAG acyltransferase (PDAT, EC 2.3.1.158) also performs DAG acylation using phospholipids as acyl donors [7, 8].

Eukaryotic DGATs are mainly divided into two ER integral membrane protein families [9–11], which do not share sequence homology [12]. DGAT1 is a member of the membrane-bound *O*-acyltransferase (MBOAT) superfamily [13]. DGAT2 belongs to a family including monoacylglycerol acyltransferases (EC 2.3.1.22) and wax monoester synthases (EC 2.3.1.75) [4]. In mammals, both DGATs are responsible for TAG production [14, 15] and presumably have non-redundant physiological functions [16–18]. In *Saccharomyces cerevisiae*, the most significant contribution is from the DGAT2 family [19]. In contrast, in *Yarrowia lipolytica* a DGAT1 family member plays a major role in TAG synthesis [5]. Several plant DGAT1 proteins are involved in seed lipid accumulation. For example, inactivation of *Arabidopsis thaliana* DGAT1 (AtDGAT1) results in up to a 45% decrease in seed oil content [20]. Enzymes from the DGAT2 family are major actors in various plants accumulating unusual fatty acids (FAs), such as eleostearic acid in Tung tree (*Vernicia fordii*) [11] or ricinoleic acid in Castor bean (*Ricinus communis*) [21]. A third family of cytosolic DGAT enzymes (DGAT3) was also identified in plants [22, 23], suggesting that TAG synthesis may also occur out of the membrane. Recently, another MBOAT superfamily member was identified in *Euonymus alatus* (EaDAcT) seeds as a distinct DGAT using acetyl-CoA as the acyl donor [24].

The search for factors regulating *Elaeis guineensis* seed oil content and FA composition recently led Dussert *et al*. [25] to identify several genes overexpressed during oil accumulation in oil palm fruit and seed tissues. Using a transcriptomics approach, two *EgDGAT1* paralogs were identified. The *EgDGAT1-1* gene is expressed in palm kernel (endosperm and embryo) whereas *EgDGAT1-2* mRNA accumulates specifically in the mesocarp. Medium-chain FAs (MCFA) represent 73% of total FAs stored in the mature endosperm tissue with lauric acid being the most abundant FA (49% of total FAs) [25]. *EgDGAT1-1* is overexpressed in the endosperm at the onset of oil accumulation suggesting it transfers MCFA at the *sn*-3 position of DAG.

Because of their impact on oil yield and quality, DGAT proteins are exciting targets for the food industry [26] and for biotechnological approaches in plants [27] and microorganisms [28]. The availability of sequenced genomes has increased the number of putative DGAT sequences, all of which are possible candidate genes for metabolic engineering. Functional characterization can now be performed by heterologous expression in mutant yeast strains [5, 19] devoid of neutral lipids. In this report we characterized oil palm EgDGAT1-1 for the first time through heterologous expression in a *Y*. *lipolytica* mutant strain unable to produce neutral lipids [5]. EgDGAT1-1 activity is sufficient to restore TAG accumulation in neosynthesized LDs. Compared to AtDGAT1, another plant DGAT1 incorporating long-chain C18:1 and C20:1 FA [3, 29], EgDGAT1-1 showed contrasted substrate specificity with a marked preference for MCFAs. Proteomics analysis showed that EgDGAT1-1 expression was the main change in the microsomal proteome observed when TAG synthesis was restored.

## Materials and Methods

### *In silico* analysis of the EgDGAT1-1 sequence

The *EgDGAT1-1* gene sequence was retrieved from the transcriptome analysis reported in Dussert *et al*. [25]. A six-frame translation was performed using BioEdit software [30]. After removing the residues before the first methionine of the longest sequence, a 512 amino acid coding sequence was obtained and aligned using the Standard Protein BLAST software (blastp) and the National Center for Biotechnology Information (NCBI) non-redundant protein sequence database. A multiple sequence alignment was performed using the Clustal Omega program [31] and formatted using BioEdit [30]. The TMpred program [32] was used for topological organization prediction.

### Cloning of EgDGAT1-1 in a *Y*. *lipolytica* expression vector

The EgDGAT1-1 and AtDGAT1 (GenBank accession number AAF19262) coding sequences with a C-terminal six histidine tag were synthesized by Eurofins MWG Operon (Ebersberg, Germany). Sequence codons were optimized for expression in *Y*. *lipolytica*. Synthetic sequences were introduced into the BamHI/AvrII restriction sites of the JMP62 vector [5] under the control of the strong constitutive yeast TEF promoter. Constructs were sequenced to confirm the absence of mutations.

### Construction of *Y*. *lipolytica* strains and culture conditions

The *Y*. *lipolytica* quadruple mutant strain JMY1877 (*MATA leu2-270 ura3-302 Δdga1 Δlro1 Δare1 Δdga2*) [5] was transformed using the lithium acetate method [33] either with the pTEF-*EgDGAT1-1*-*URA3*, the pTEF-*AtDGAT1*-*URA3* cassette or the empty pTEF-*URA3* cassette (control strain) from the NotI linearized JMP62 recombinant and native vectors. Yeast were grown in uracil deficient medium containing 0.17% (*w/v*) yeast nitrogen base (YNB) supplemented with 0.5% (*w/v*) ammonium sulfate and 0.2% (*w/v*) casamino acids (BD, Le Pont de Claix, France) and YP medium containing 2.2% (*w/v*) peptone and 1.1% (*w/v*) yeast extract (Euromedex, Mundolsheim, France). The carbon sources (Sigma-Aldrich, Saint-Quentin Fallavier, France) were 2% (*w/v*) glucose for YPG and YNBG media or 0.02% (*w/v*) glucose and 2% (*w/v*) lauric acid methyl ester (LAME) emulsified by sonication with 0.2% (*w/v*) Tween 20 for YPL medium. LAME was used instead of lauric acid which is solid at temperatures below 44°C. Transformants were selected on YNBG plates. Cells from three independent transformants were grown for one day in YPG medium. Yeasts were diluted in YPG or YPL medium to an optical density of 0.5 for an 18h culture. All cultures were performed in baffled Erlenmeyer flasks at 28°C and 200 r.p.m.

### Fluorescence microscopy

Yeast were washed with Dulbecco’s phosphate buffered saline (Eurobio, Courtaboeuf, France) and incubated for 15 min at 200 rpm and 28°C in a Nile Red solution (1 mg/l, Sigma-Aldrich). Cells were washed again before examination with a Zeiss Axio Imager microscope (Zeiss, Le Pecq, France) with fluorescence (filter set 43HE) and Nomarski optics and a Roper CoolSnap HQ2 camera coupled to a Zeiss AxioVision driver. Images were acquired using an EC Plan-Neofluar 100x/1.30 Oil M27 objective with a numerical aperture of 1.3.

### Subcellular fractionation

#### Yeast cell disruption

All steps were performed at 4°C on three independent transformants. Yeast were cultured in 100 ml YPG then centrifuged at 1300 *g* for 10 min and washed with ultrapure water. After resuspension in lysis buffer (10 mM HEPES, 10 mM KCl, 0.1 mM EDTA, 0.1 mM EGTA, pH 7.5) supplemented with protease inhibitors (cOmplete, Mini, EDTA-free, Roche, Indianapolis, USA), cells were disrupted with a One Shot cell disruptor (Constant Systems Ltd, Daventry, UK) at a pressure of 2.97 kbar. Cellular debris were removed by centrifugation at 12 000 *g* for 10 min.

#### Lipid droplet purification by sucrose density gradients

Sucrose density gradients were assembled with the supernatant as previously described [34] and subjected to ultracentrifugation for 90 min at 4°C and 150,000 *g* in a SW41 Ti swing-out rotor (Beckman Coulter, Villepinte, France). The floating lipid layer, or the corresponding volume from the control strain, was collected and stored at -80°C.

#### Microsome extraction

Microsomes from EgDGAT1-1 transformants or the control strain were purified following a procedure similar to LD isolation (except without a sucrose density gradient) and ultracentrifugation for 90 min at 4°C and 100000 *g* according to Bouvier-Navé *et al*. [35]. Microsomal pellets were resuspended in 100 mM Tris-HCl pH 7 containing 20% (*v/v*) glycerol and frozen at -80°C [35].

### Proteomic analysis of recombinant *Y*. *lipolytica* quadruple mutant microsomes

Proteins were quantified using Bio-Rad Protein Assay (Bio-Rad, Marnes-la-Coquette, France) and separated using 10% Bis-Tris NuPAGE gels with MOPS SDS running buffer and NuPAGE LDS sample buffer (Life Technologies, Saint Aubin, France) containing 50 mM DTT according to the manufacturer’s recommendations. Twenty μg of microsomal protein separated over 1 cm were stained with Coomassie blue (G-250) according to Neuhoff *et al*.[36]. Each lane was cut in five small cubes (# 2 mm) collected in 96-well microplates. In-gel trypsin digestion was performed with the Progest system (Genomic Solution) according to Abdallah *et al*. [37] after protein reduction (10 mM DTT) and alkylation (55 mM iodoacetamide). NanoLC-MS/MS analysis was performed using an Ultimate 3000 LC system (Dionex, Sunnyvale, CA) connected to a LTQ Orbitrap mass spectrometer (Thermo Electron, Waltham, MA) according to Blein-Nicolas *et al*. [38]. Database searches were performed using X!Tandem (Release 2013.9.1.0; http://www.thegpm.org/TANDEM). Enzymatic cleavage was declared as trypsin digestion with one possible miscleavage. Cys carboxyamidomethylation was set as static modification whereas Met oxidation, Nter deamidation and Nter acetylation were set as variable modifications. Precursor mass tolerance was 10 ppm and fragment mass tolerance was 0.5 Da. Identifications were performed using the *Y*. *lipolytica* database of opened reading frames obtained from Génolevures [39]. Identified proteins were filtered and grouped using the X!Tandem pipeline v3.3.3 (http://pappso.inra.fr/bioinfo/xtandempipeline/). Data were filtered according to a peptide E value smaller than 0.05 with a minimum of two peptides to identify a protein. Annotated proteins were manually curated in functional classes.

### Lipidomic analyses

#### Yeast cell disruption

Cells were harvested by centrifugation at 1300 *g* for 10 min and washed three times with a solution containing 0.5% (*w/v*) BSA and 0.9% (*w/v*) NaCl. Disruption was performed in water at 2.97 kbar and yeast lysates were frozen at -80°C before freeze-drying.

#### Lipid extraction

Total lipids from yeast cells or LDs were extracted following a method developed by Folch *et al*. [40]. One hundred mg of dry cell lysate or floating layers from sucrose density gradients were treated as previously described [34]. Lipids were stored at -25°C.

#### Separation of lipid classes by High-Performance Thin-Layer Chromatography (HPTLC)

Lipids were solubilized in chloroform/methanol 2/1 (*v/v*) and spotted onto silica-coated glass plates (HPTLC Silica gel 60, Merck, Fontenay Sous Bois, France) pre-washed in 2-propanol using a CAMAG automatic TLC sampler ATS3 (Chromacim, Moirans, France). Plates were developed using a CAMAG automatic developing chamber ADC2 with hexane/diethyl ether/acetic acid 80/20/2 (*v/v/v*). Lipids were identified based on the migration of lipid standards (Sigma-Aldrich) after staining with 5% (*w/v*) phosphomolybdic acid for 30 min at 100°C. Plates were scanned using an Epson expression 1680 Pro Scanner.

#### Total fatty acid determination

The FA content and composition of yeast strains were determined based on a method developed by Browse *et al*. [41]. FAs from twenty-five mg of dry cell lysate were transmethylated and FA methyl esters (FAMEs) were extracted and analyzed by gas chromatography with flame ionization detection (GC-FID) according to Froissard *et al*. [42].

#### Compositional analysis of yeast TAGs by ultra-high performance liquid chromatography coupled to high-resolution tandem mass spectrometry (UHPLC—HRMS/MS)

Neutral lipids were obtained upon fractionation of total lipids using an Isolute solid phase extraction (SPE) Aminopropyl column (ALLTECH France Sarl, Epernon, France) according to Beopoulos *et al*. [5]. TAG composition was analyzed by UHPLC-HRMS/MS according to Gallart-Ayala *et al*. [43].

## Results

### EgDGAT1-1 contains major conserved DGAT1 sequence motifs

**Fig 1:**
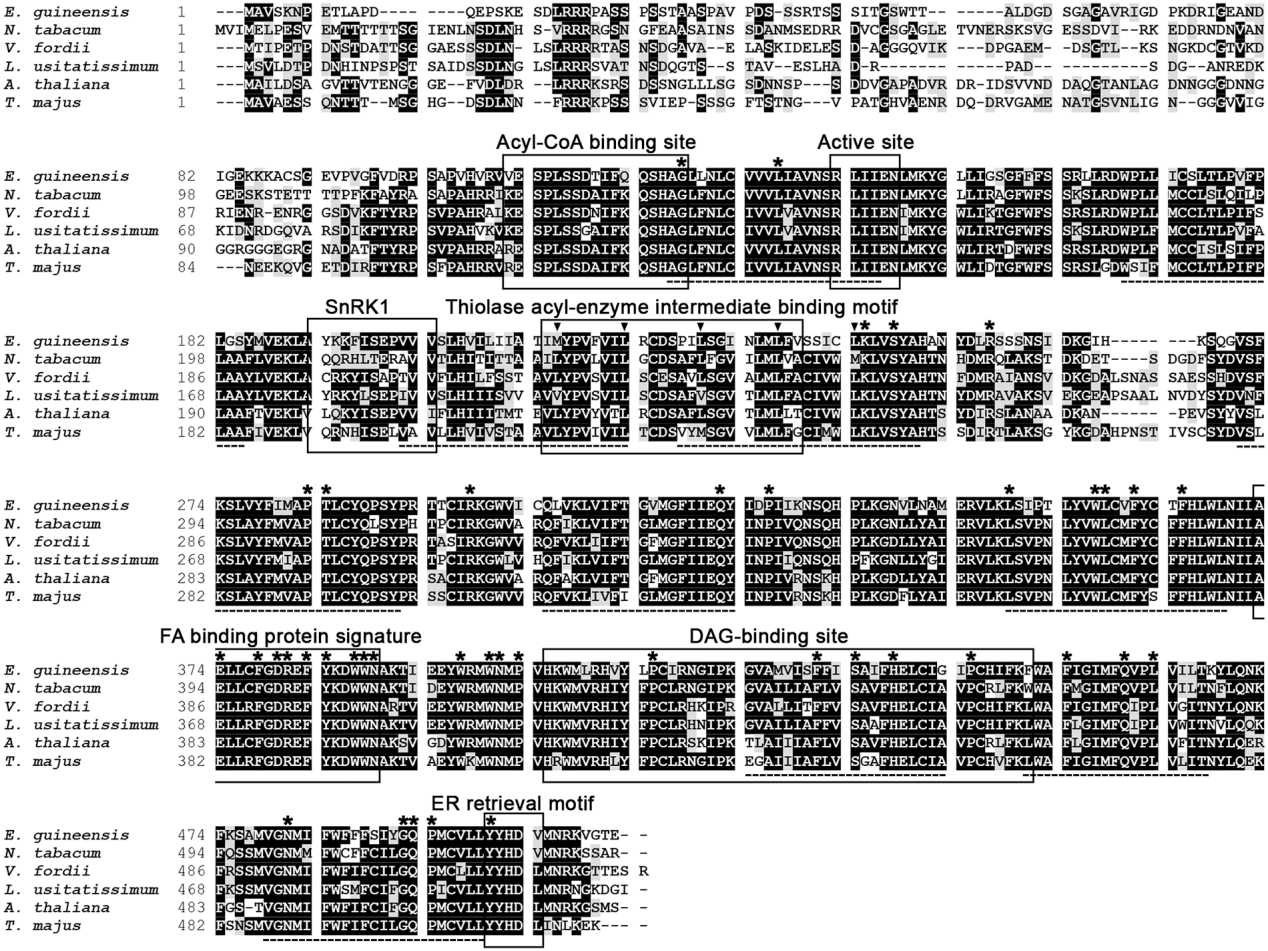
Comparison of the EgDGAT1-1 sequence with biochemically-characterized plant DGAT1 proteins. The EgDGAT1-1 amino acid sequence was aligned, using Clustal Omega [31], with five biochemically-characterized plant DGAT1 proteins: *Nicotiana tabacum* (GenBank accession number AAF19345), *V*. *fordii* (ABC94471), *Linum usitatissimum* (AHA57450) *A*. *thaliana* (AAF19262), and *Tropaeolum majus* (AAM03340). Identical residues are highlighted in black and similar residues are shaded. Transmembrane domains predicted by the TMpred program are underlined (hatched line). Several conserved motifs are boxed, including the acyl-CoA binding site [3, 45], active site [3, 45], typical SnRK1 protein kinase targeting motif [45, 46], thiolase acyl-enzyme intermediate binding motif [45, 46], FA binding protein signature [45], DAG-binding site [45, 46] and ER retrieval motif [11]. A leucine zipper motif [6, 47] is highlighted by vertical arrows. The 41 invariant residues among 55 DGAT1 sequences from animals, plants and fungi are shown with asterisks [48].

The *EgDGAT1-1* gene sequence was retrieved from the transcriptome analysis of Dussert *et al*. [25]. The sequence was translated in all six frames to generate a 512 amino acid sequence, after removal of the residues located before the first methionine. The NCBI non-redundant protein database was searched for similar sequences using blastp software. A predicted diacylglycerol *O*-acyltransferase 1-like protein from *E*. *guineensis* (NCBI Reference Sequence XP_010924968) with a strictly identical amino acid sequence was retrieved. This sequence was predicted by automated computational analysis from whole genome shotgun sequencing (NW_011550756.1) of *E*. *guineensis* chromosome 6. The EgDGAT1-1 amino acid sequence shares a high level of sequence identity with the biochemically-characterized plant DGAT1 [11, 35, 44, 45]. We found 64% identity with *Linum usitatissimum* (GenBank accession number AHA57450), 62% with *A*. *thaliana*, 61% with *Nicotiana tabacum* (AAF19345) or *V*. *fordii* (ABC94471) and 59% with *Tropaeolum majus* DGAT1 (AAM03340). EgDGAT1-1 was aligned with these five plant DGAT1 using Clustal Omega [31] (Fig 1). The results highlighted a set of conserved features, originally identified by Zou *et al*. [46], Hobbs *et al*. [47] and Jako *et al*. [3] in AtDGAT1 and Xu *et al*. [46] in *T*. *majus* DGAT1, that are all present in EgDGAT1-1. The ER retrieval motif shown to be necessary for the ER targeting of Tung tree DGAT1 [11] is also found in EgDGAT1-1. The protein also contains ten putative transmembrane domains in highly conserved hydrophobic regions and the 41 residues which Cao [48] showed are absolutely conserved among DGAT1 sequences from plants, animals and fungi.

### Expression of EgDGAT1-1 restores neutral lipid accumulation in the *Y*. *lipolytica* JMY1877 strain

**Fig 2:**
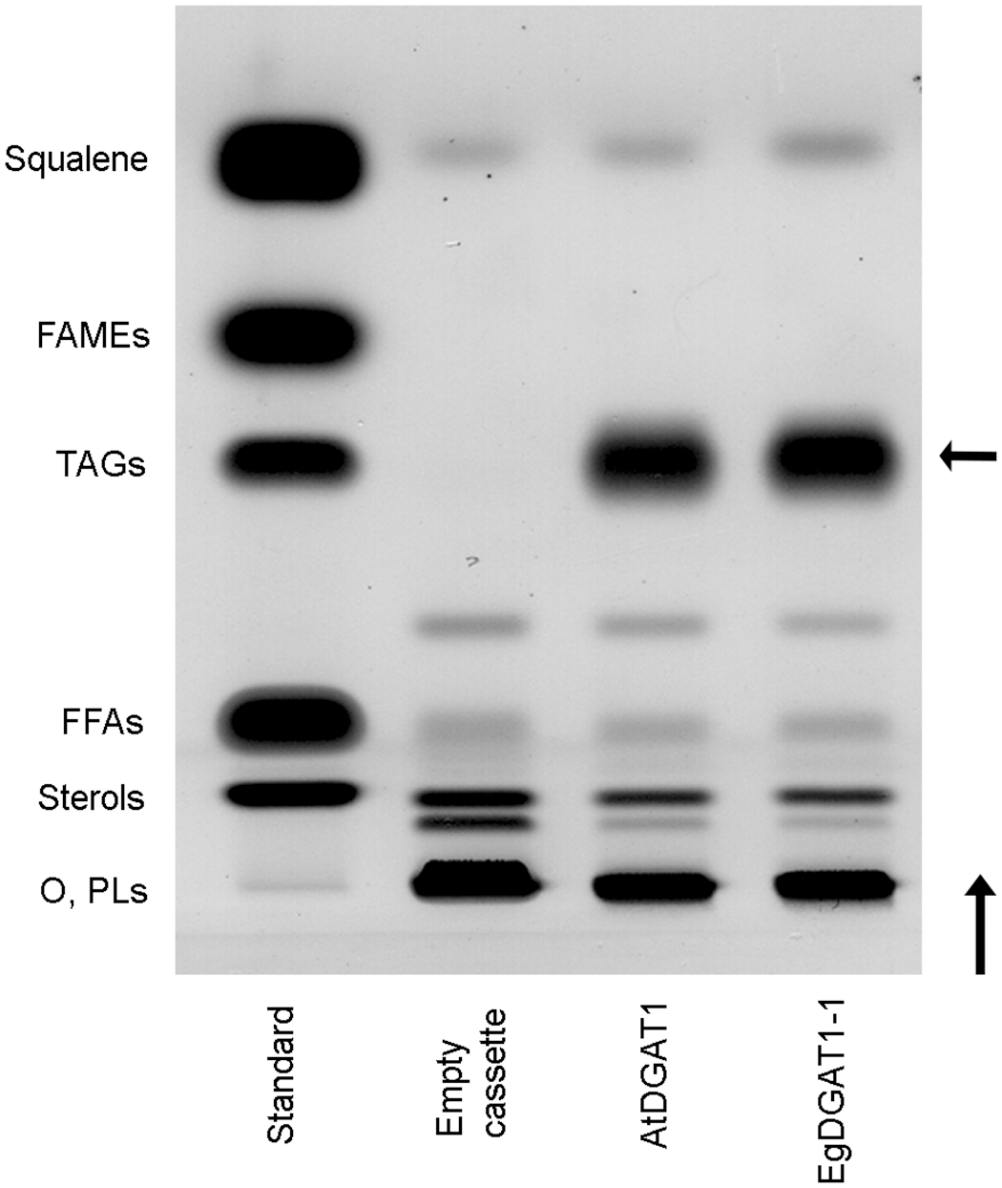
Separation of the lipid classes from the recombinant JMY1877 strains by HPTLC. JMY1877 recombinant strains transformed with the empty cassette or expressing AtDGAT1 or EgDGAT1-1 were grown for 18 h. Lipids corresponding to 0.4 mg dry cell lysate or lipids from the standard (four micrograms each) were separated on a silica plate. This experiment is representative of three independent cultures. A vertical arrow indicates the direction of migration and a horizontal arrow points out the position of TAG spots in strains expressing AtDGAT1 or EgDGAT1-1. O: origin of migration; PLs: phospholipids; FFAs: free fatty acids; TAGs: triacylglycerols; FAMEs: fatty acid methyl esters.

**Fig 3:**
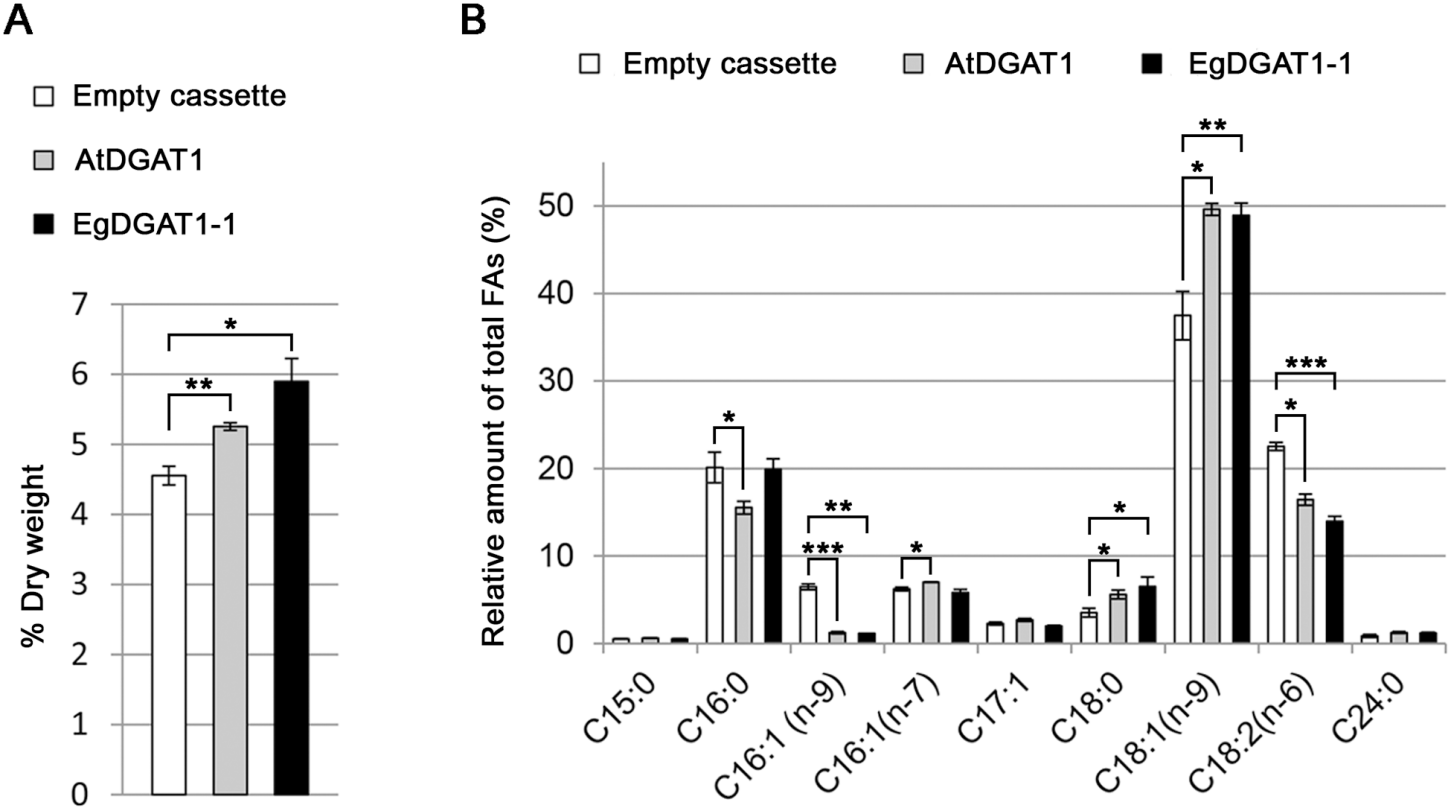
FA content (A) and composition (B) of yeast lipids. JMY1877 recombinant strains transformed with the empty cassette or expressing AtDGAT1 or EgDGAT1-1 were grown for 18 h. FAs from twenty-five milligrams of dry cell lysate were transmethylated. The resulting FAMEs from three independent cultures were identified and quantified by GC-FID. (**A**) FA content values are expressed in percentage of dry cell weight per strain. Asterisks indicate statistically significant differences according to a *t*-test (*P<0.05; **P<0.01). (**B**) FA composition values are expressed as relative amount of each FA per strain. Asterisks indicate statistically significant differences according to a *t*-test (*P<0.05; **P<0.01; ***P<0.001).

The *EgDGAT1-1* and *AtDGAT1* sequences were integrated into the genome of the *Y*. *lipolytica* JMY1877 mutant strain under the control of the TEF promoter. This strain lacks four acyltransferases and is completely defective in neutral lipid biosynthesis [5]. The diacylglycerol acyltransferase activity of AtDGAT1 was previously demonstrated in a yeast expression system [35, 49] and was used as the positive control in the present study. The JMY1877 strain transformed with the empty pTEF-*URA3* cassette was the negative control. Total lipids were extracted from recombinant yeast and separated on HPTLC plates (Fig 2). EgDGAT-1 and AtDGAT1 expression restored TAG production in the JMY1877 strain. Total FAs from the different strains were transmethylated and quantified using GC-FID analysis. Expression of both DGAT1 proteins significantly increased the total FA content in JMY1877 (Fig 3A). Cells expressing AtDGAT1 (5.3% total FA content) and EgDGAT1-1 (5.9%) contained 15% and 29% more FAs than the negative control (4.6%), respectively. The expression of both DGAT1 proteins induced a significant increase in the proportion of long chain oleic acid (Fig 3B).

**Fig 4:**
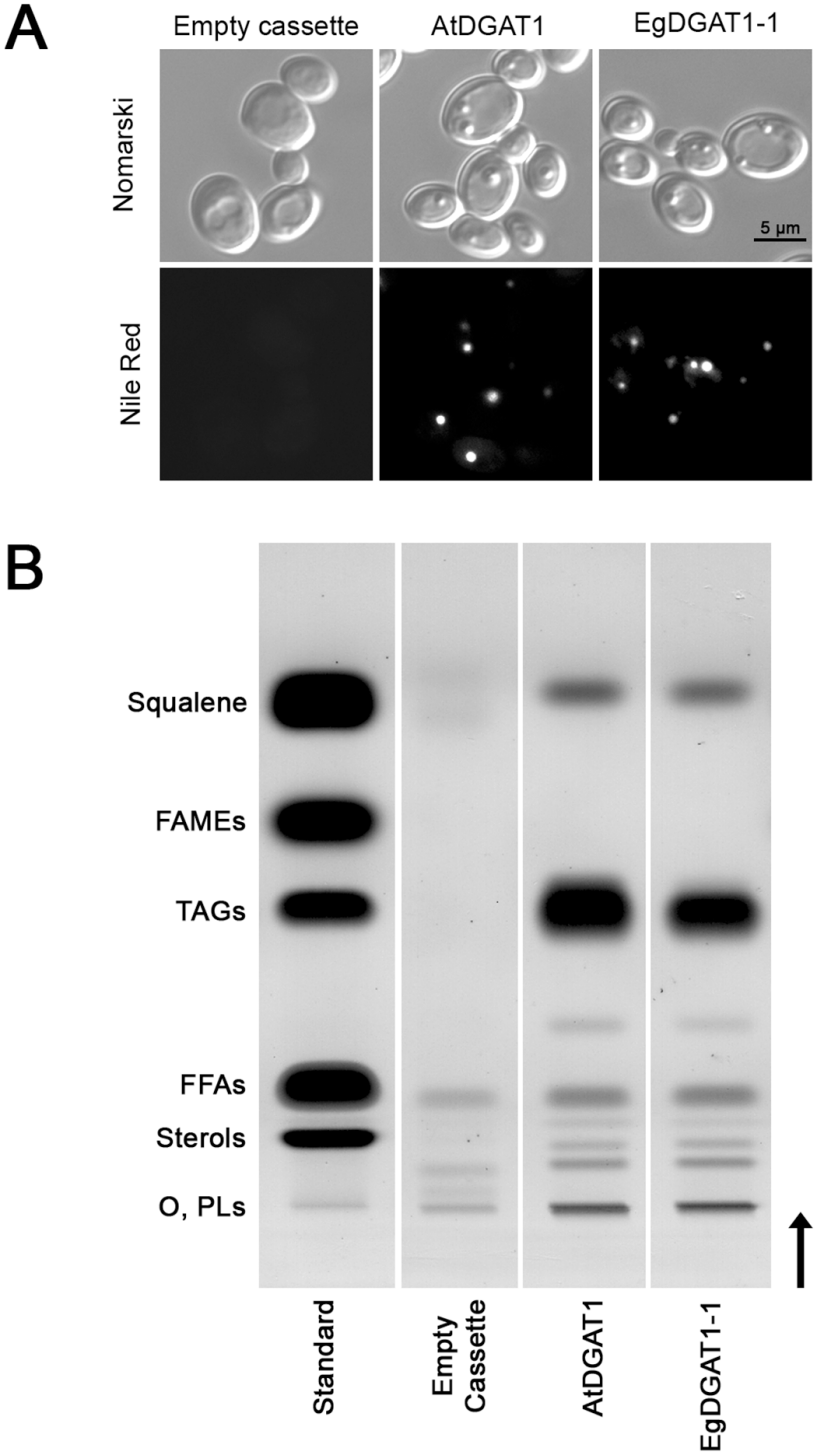
Microscopic observation (A) and lipid classes (B) of neosynthesized lipid droplets. (**A**) JMY1877 recombinant strains transformed with the empty cassette or expressing AtDGAT1 or EgDGAT1-1 were grown for 18 h. Cells were observed under the microscope with phase contrast (Nomarski) or fluorescence (Nile Red). (**B**) Separation of floating layer lipids by HPTLC. Lipids extracted from the 150,000 *g* floating layer of each recombinant JMY1877 strain or lipids from the standard (four micrograms each) were separated on silica plates. A vertical arrow indicates the direction of migration. O: origin of migration; PLs: phospholipids; FFAs: free fatty acids; TAGs: triacylglycerols; FAMEs: fatty acid methyl esters.

Neutral lipids such as TAGs are stored in LDs. To determine whether EgDGAT1-1 activity is associated with LD biosynthesis, cells were stained with the fluorescent neutral lipid dye, Nile red, and observed under a fluorescence microscope (Fig 4A). AtDGAT1 and EgDGAT1-1 expressing strains accumulated the neutral lipid dye in small cytoplasmic inclusions, which were absent in the control strain. To confirm the presence of neosynthesized LDs in DGAT1 expressing strains, a LD purification protocol was applied to lysates of the recombinant yeast strains. A floating layer in the top fraction of the 150,000 *g* supernatant was only found for the plant DGAT1 expressing strains. The lipids in this top fraction were extracted and separated on HPTLC plates (Fig 4B). Large amounts of TAGs accumulated in floating layers of the AtDGAT1 and EgDGAT1-1 expressing strains only, consistent with biogenesis of LDs. Both floating layers also contained noticeable amounts of squalene.

### EgDGAT1-1 and AtDGAT1 generate different TAG families

**Fig 5:**
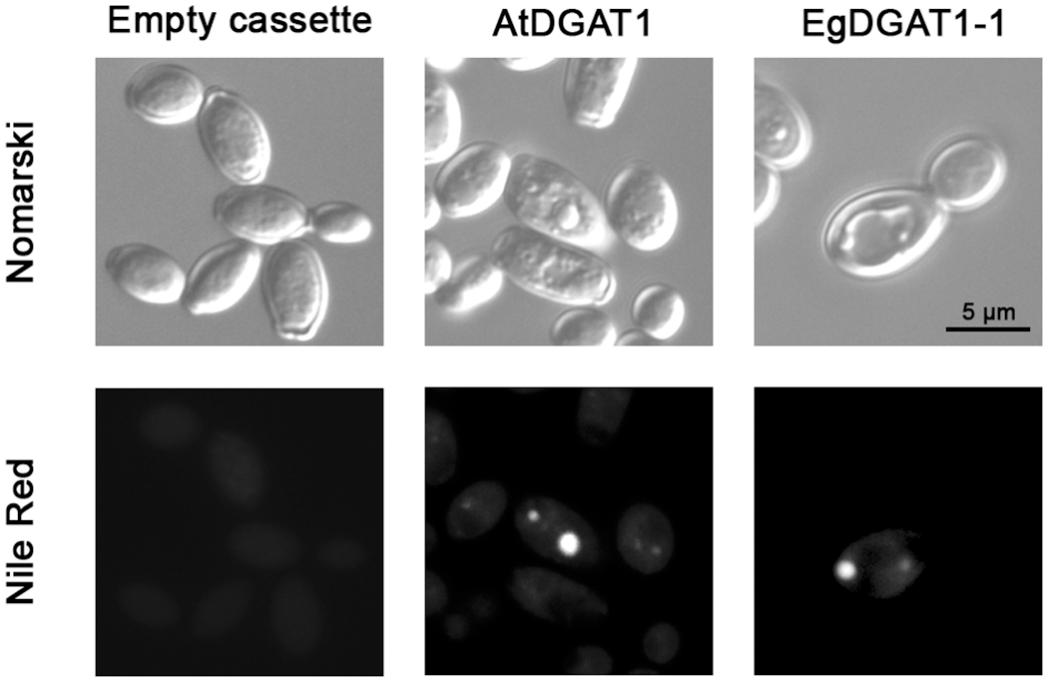
Microscopic observation of JMY1877 transformants grown in 2% LAME supplemented YP medium. JMY1877 recombinant strains transformed with the empty cassette or expressing AtDGAT1 or EgDGAT1-1 were grown for 18 h in YPL medium. Cells were observed under the microscope with phase contrast (Nomarski) or fluorescence (Nile Red).

**Fig 6:**
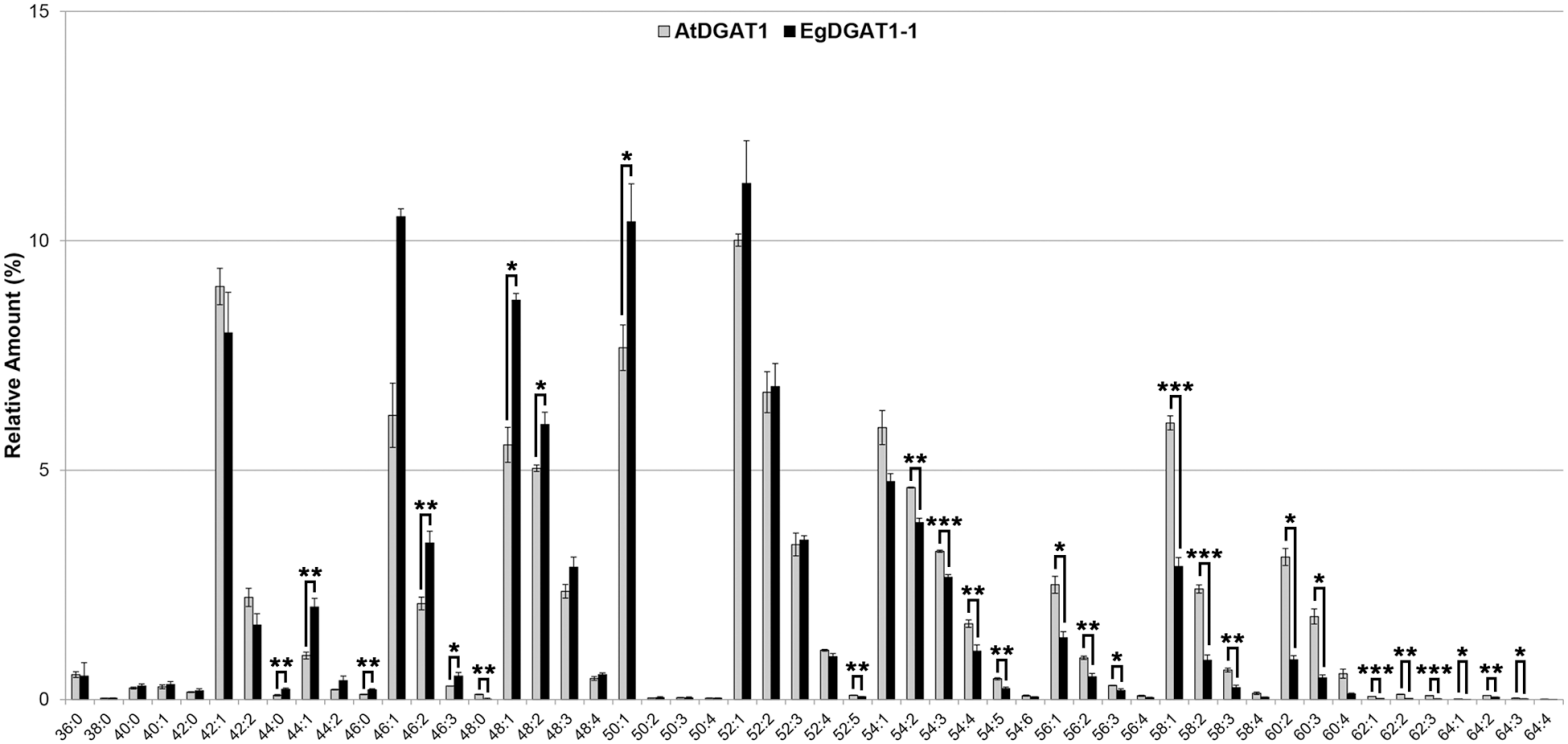
TAG profiling of recombinant yeast strains grown in 2% LAME supplemented YP. JMY1877 recombinant strains expressing AtDGAT1 or EgDGAT1-1 were grown for 18 h in YPL medium. Lipids were extracted from thirty milligrams of dry cell lysate. TAGs from three independent cultures were separated by SPE and composition was analyzed by UHPLC-HRMS/MS. Values are expressed as the relative amount of each TAG species per strain. For each species the overall number of carbon atoms of the three esterified FA chains and the overall number of double bonds is specified. Asterisks indicate statistically significant differences according to a *t*-test (*P<0.05; **P<0.01; ***P<0.001).

*In planta* EgDGAT1-1 is expressed in oil palm kernel at the onset of MCFA accumulation [25]. *Y*. *lipolytica* does not naturally produce MCFAs but this oleaginous yeast is able to import FAs from its environment [50]. In order to mimic *in planta* conditions, transformants were grown in a medium enriched with LAME. After 18 h of culture in a 2% LAME supplemented medium, cells were analyzed under a fluorescence microscope (Fig 5). LDs were observed by phase contrast microscopy and Nile red fluorescence in AtDGAT1 and EgDGAT1-1 expressing strains. TAGs were extracted and analyzed using UHPLC—HRMS/MS (Fig 6) in order to characterize the different families. The EgDGAT1-1 expressing strain accumulated significantly higher levels of medium and long chain TAGs (C44 to C50) than the AtDGAT1 expressing strain, with around twice more saturated and unsaturated medium-chain C44 and C46. The accumulation of long and very-long chain TAGs (C54 to C64) was significantly higher in the AtDGAT1 expressing strain, which contained at least twice as much C56 to C64 with even three times more C60 than the EgDGAT1-1 expressing strain. All major TAG species contained at least one unsaturated FA.

### EgDGAT1-1 is a microsomal acyltransferase which restores TAG accumulation without major reorganization of the microsomal proteome

The ER is involved in numerous biological processes including protein and lipid synthesis. Microsomes are vesicular fragments of the ER obtained upon cell disruption. DGAT1 have been described as ER integral membrane proteins. The microsomal proteomes of the EgDGAT1-1 expressing strain and the control strain were characterized using nanoLC-MS/MS. Differential proteome analysis was carried out to search for the presence of EgDGAT1-1 and to identify protein variation associated with the restoration of neutral lipid biosynthesis.

The total number of spectra per replicate did not vary much (between 12730 and 10048 spectra) suggesting good reproducibility of the whole process. Only proteins for which peptides were identified in the three biological replicates of at least one strain were considered as significant. According to this criterion, 764 different proteins were retained among the 1194 proteins identified. Six different peptides belonging to EgDGAT1-1 were found in all the samples derived from the yeast expressing EgDGAT1-1, and not in the control strains. EgDGAT1-1 coverage was 14.8% and was ranked 320 among 764 microsomal proteins observed in each transformant, suggesting a low abundancy.

**Fig 7:**
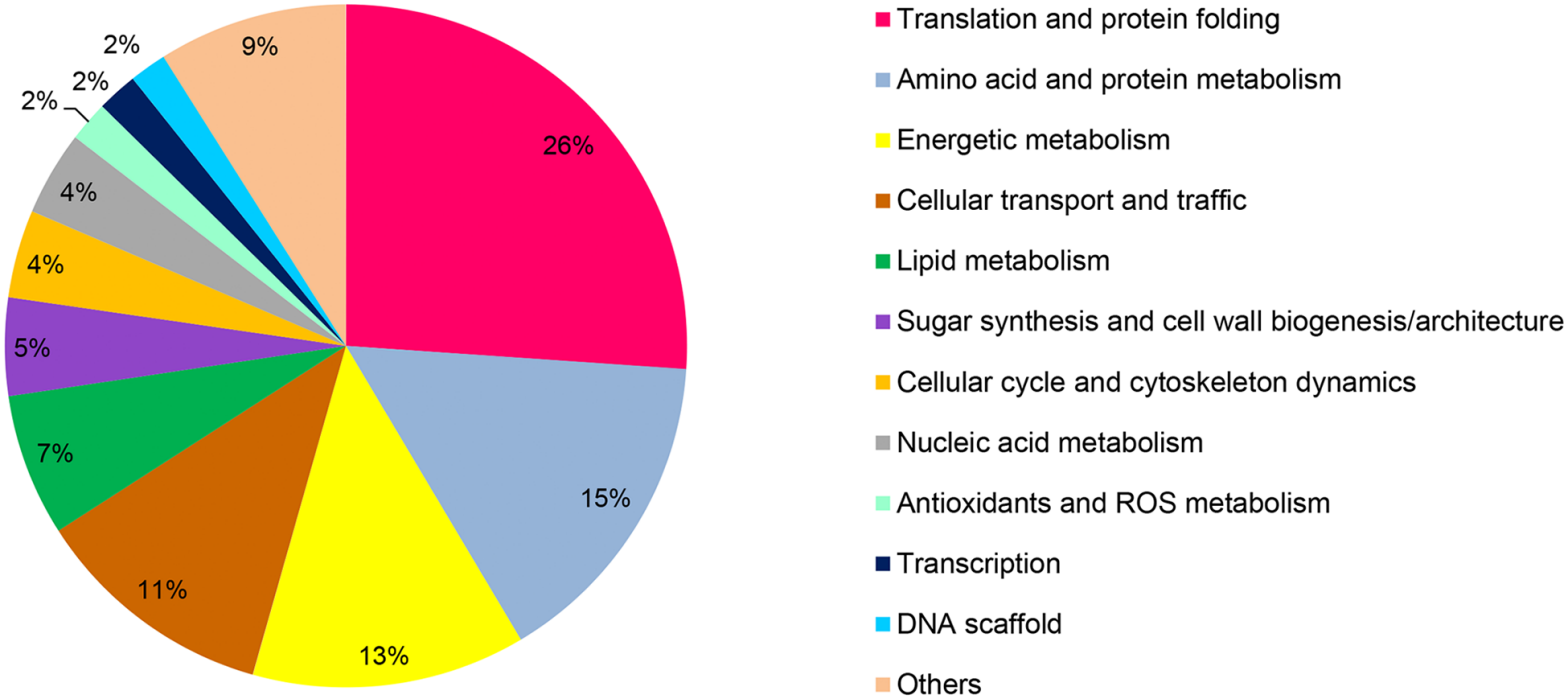
Functional annotation of the microsomal proteins present in the JMY1877 transformants. Proteins in 100,000 *g* microsomes from three independent transformants of the control strain or the EgDGAT1-1 expressing strain were digested in gel. Peptides were separated by liquid chromatography and analyzed with a LTQ Orbitrap mass spectrometer using a nano-electrospray interface. Proteins found in the three transformants of each strain were annotated using the Génolevures annotated sequence database [39] and manually curated into twelve functional classes. Six hundred and twenty-five of the 744 proteins could be sorted into twelve functional classes.

Functional annotation of the 744 microsomal proteins present in the three biological replicates of both samples was attempted using the Génolevures annotated sequence database [39]. Six hundred and twenty-five of the 744 proteins could be sorted into twelve classes (Fig 7 and Tables A-L in S1 Table). The remaining 119 proteins were not identified because of the lack of similarity with known proteins. The largest class contained proteins related to translation and protein folding (26%) and was followed by protein metabolism (15%). Seven percent of microsomal proteins were associated with lipid metabolism.

We then focused on the 15 most abundant proteins found in the control strain with a mean protein abundance index (PAI) per replicate comprised between 9.9 and 4.0 (Table 1). These 15 proteins were mostly associated with translation and protein folding (10 proteins). The relative abundance of most proteins (11 out of 15) did not vary significantly in the EgDGAT1-1 expressing strain.

**Table 1:** The top 15 proteins in the microsomes of JMY1877 transformed with the empty cassette.

Protein ID	Mean PAI per control replicate	Mean PAI per EgDGAT1-1 replicate	*t*-test	Classification	Protein description in Génolevures database	Present in [51]
YALI0C09141p	9.9	10.9		Translation and protein folding	*Y*. *lipolytica* Elongation factor 1- alpha (EF-1-alpha)	Yes
YALI0B22066p	7.8	8.9		Cellular transport and traffic	highly similar to *S*. *cerevisiae* YGL008c PMA1 H^+^-transporting P-type ATPase major isoform plasma membrane	Yes
YALI0F24255p	7.8	8.3		Unknown function	no similarity	No
YALI0F25289p	5.6	6.6		Translation and protein folding	highly similar to *S*. *cerevisiae* YER103w SSA4 of HSP70 family	No
YALI0C07953p	5.5	5.4		Translation and protein folding	highly similar to *Candida albicans* CaHSC82 HSP90 homolog	No
YALI0D08184p	5.5	6.7		Translation and protein folding	highly similar to *S*. *cerevisiae* YER103w SSA4 of HSP70 cytosolic family	No
YALI0F16819p	5.3	6.3	**	Energetic metabolism	highly similar to *S*. *cerevisiae* YHR174w ENO2 or YGR254w ENO1 enolases	Yes
YALI0E13706p	5.1	3.2	**	Translation and protein folding	*Y*. *lipolytica* dnaK-type molecular chaperone involved in ER translocation of secretory proteins (BiP homologue)	No
YALI0D22352p	4.9	7.0	**	Translation and protein folding	highly similar to *S*. *cerevisiae* YER103w SSA4 of HSP70 cytosolic family	No
YALI0C17347p	4.7	4.8		Translation and protein folding	highly similar to *S*. *cerevisiae* YJR045c HSP70-related protein SSC1 mitochondrial precursor (Endonuclease SCEI 75 kDa subunit)	Yes
YALI0A00352p	4.7	5.5		Translation and protein folding	highly similar to *S*. *cerevisiae* YOR133w EFT1 or YDR385W EFT2, paralogs encoding elongation factor 2	Yes
YALI0E35046p	4.4	5.3		Translation and protein folding	highly similar to *S*. *cerevisiae* YER103w SSA4 of HSP70 cytosolic family	No
YALI0D09361p	4.3	4.5		Energetic metabolism	highly similar to *S*. *cerevisiae* YLR304C Aconitate hydratase mitochondrial precursor (Citrate hydro-lyase)	No
YALI0E13277p	4.1	3.6		Translation and protein folding	*Y*. *lipolytica* Elongation factor 3 (EF-3)	No
YALI0D08272p	4.0	4.6	*	Cellular cycle and cytoskeleton dynamics	*Y*. *lipolytica* Actin	Yes

*P<0.05;

**P<0.01

Proteins from three independent transformants of 100,000 *g* microsomes of the control strain or the EgDGAT1-1 expressing strain were digested in gel. Peptides were separated by liquid chromatography and analyzed with a LTQ Orbitrap mass spectrometer using a nano-electrospray interface. Proteins found in the three transformants of each strain were ranked according to their mean PAI. Only the 15 most abundant proteins of the control strain are displayed, their PAI are compared with corresponding ones of the EgDGAT1-1 expressing strain. Asterisks indicate statistically significant differences according to a *t*-test (*P<0.05; **P<0.01). Homologs found in microsomes of *P*. *pastoris* [51] are specified.

The variation in abundance of microsomal proteins upon EgDGAT1-1 expression was analyzed from the ratio of the mean PAI observed for each strain. We searched for proteins that were more abundant than EgDGAT1-1 and with at least a two-fold increase in expression compared with the control proteome. Sixteen proteins meeting these criteria were found. In comparison with the control strain, most proteins (14 out of 16) displayed significant differences in abundance. Only ten were functionally annotated and belonged to six different classes (Table 2). Thus the difference between the two strains was limited to a small number of proteins and no specific functional class.

**Table 2:** List of 16 proteins which were more abundant than EgDGAT1-1 and whose expression increased at least two-fold in the EgDGAT1-1 expressing strain compared to the control strain.

Protein ID	Mean PAI EgDGAT1-1/ control	*t*-test	Classification	Protein description in Génolevures database
YALI0F01210p	5.1	**	Cellular transport and traffic	similar to *Candida albicans* CaAQY1 putative plasma membrane and water channel protein
YALI0E34309p	4.4	*	Unknown function	weakly similar *S*. *cerevisiae* YPL210c SRP72 signal recognition particle protein
YALI0B18282p	4.1	**	Cellular transport and traffic	similar to *Schizosaccharomyces pombe* Gluconate transport inducer 1
YALI0D09889p	4.0		Unknown function	similar to *Neurospora crassa* NCU08949. 1 hypothetical protein
YALI0D10131p	3.7	*	Others	similar to *S*. *cerevisiae* YLR044c PDC1 pyruvate decarboxylase isozyme
YALI0E31691p	3.5	*	Unknown function	weakly similar to *S*. *cerevisiae* YMR021c MAC1 metal binding activator singleton
YALI0E34749p	3.4	*	Antioxidants and ROS metabolism	similar to *S*. *cerevisiae* YGR088w CTT1 cytosolic catalase T
YALI0F09273p	2.5	**	Others	similar to *S*. *cerevisiae* YFL053w DAK2 and YML070w DAK1 Dihydroxyacetone kinases
YALI0F04169p	2.2	*	Sugar synthesis and cell wall biogenesis/ architecture	similar to *S*. *cerevisiae* YPR160w GPH1 glycogen phosphorylase
YALI0D08536p	2.2	*	Cellular cycle and cytoskeleton dynamics	some similarities with *S*. *cerevisiae* YIL105c SLM1 Phosphoinositide PI4,5P(2) binding protein
YALI0C22000p	2.2	**	Antioxidants and ROS metabolism	similar to *S*. *cerevisiae* YDR533c HSP31 Methylglyoxalase
YALI0E01782p	2.2	*	Translation and protein folding	similar to *S*. *cerevisiae* YMR290c HAS1 helicase associated with SET1P
YALI0E03718p	2.1	*	Unknown function	no similarity
YALI0D23947p	2.1	**	Cellular transport and traffic	some similarities with *S*. *cerevisiae* YDL058w USO1 intracellular transport protein
YALI0F05654p	2.0	*	Unknown function	some similarities with an uncharacterized protein from *Shewanella oneidensis*
YALI0F22693p	2.0		Unknown function	no similarity

*P<0.05;

**P<0.01

Proteins from three independent transformants of 100,000 *g* microsomes of the control strain or the EgDGAT1-1 expressing strain were digested in gel. Peptides were separated by liquid chromatography and analyzed with a LTQ Orbitrap mass spectrometer using a nano-electrospray interface. Proteins found in the three transformants of each strain were ranked according to their mean PAI. Only the 16 proteins more abundant than EgDGAT1-1 and whose expression increased at least two-fold in the EgDGAT1-1 expressing strain compared to the control strain are displayed. Asterisks indicate statistically significant differences between the mean PAI of both strains according to a *t*-test (*P<0.05; **P<0.01).

### EgDGAT1-1 N-terminal sequence harbors conserved motifs found in DGAT1 from plant storing lauric acid

**Fig 8:**
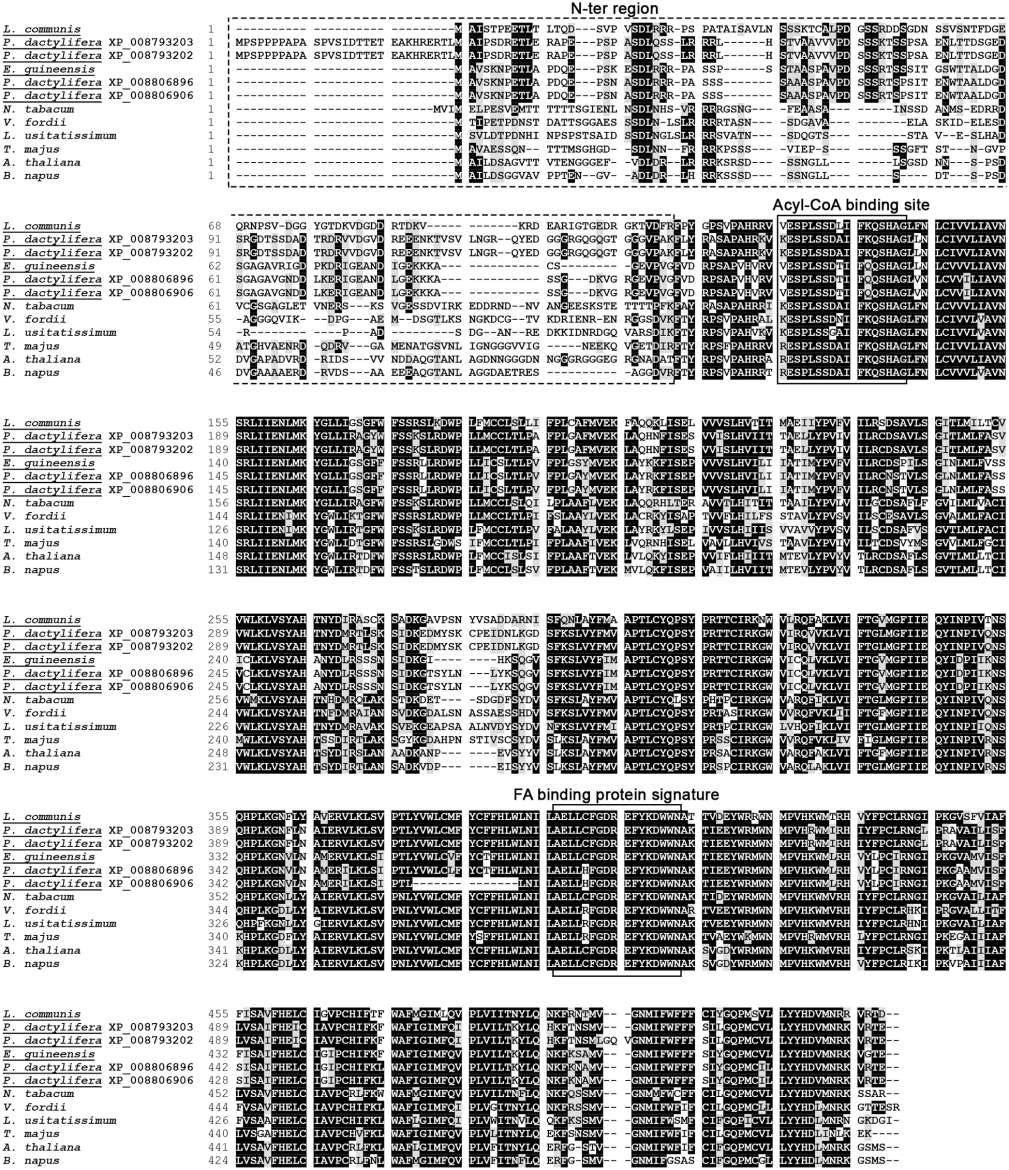
Comparison of the N-terminal sequence of EgDGAT1-1 with several plant DGAT1. The EgDGAT1-1 sequence was aligned, using Clustal Omega [31], with eleven plant DGAT1 proteins: *L*. *communis* (AHN93288), *P*. *dactylifera* (XP_008793203), *P*. *dactylifera* (XP_008793202), *P*. *dactylifera* (XP_008806896), *P*. *dactylifera* (XP_008806906), *N*. *tabacum* (AAF19345), *V*. *fordii* (ABC94471), *L*. *usitatissimum* (AHA57450), *T*. *majus* (AAM03340), *A*. *thaliana* (AAF19262), *B*. *napus* (AAD45536). The name of the plants able to store lauric acid in their seeds is underlined. The variable N-terminal region is framed with dashed lines. Conserved motif important for acyl-CoA binding [3, 45] and the FA binding protein signature [45] are boxed with straight lines.

Several DGAT1 from plant species storing medium-chain FA display the highest level of identity obtained upon alignment with EgDGAT1-1 sequence using blastp software. Among those, the putative DGAT1 from *Lindera communis* (displaying 65.7% identity), a plant storing 59.1% lauric acid in its seeds [52], and four putative DGAT1 isoforms found in *Phoenix dactylifera* also known as the date palm (displaying respectively 91%, 88.5%, 65.2% and 64.9% identity). This palm species stores limited amounts of oil in its fruit with the kernel being the only fat-storing tissue. Oleic acid (42.3%) and lauric (21.8%) are the two main fatty acids stored in *P*. *dactylifera* kernel [53]. These results suggest that one or several date palm DGAT might be able to use lauric acid as an acyl donor. However the family and isoforms involved in synthesis remain unknown. A multiple sequence alignment (Fig 8) of EgDGAT1-1 with eleven DGAT1 including those from Fig 1 and the DGAT1 from lauric acid storing plants was performed in order to search for possible molecular determinant of acyl-CoA chain length preference. As shown on Fig 1 plant DGAT1 sequences are highly identical except for the poorly conserved N-terminal region found before the first consensus motif (acyl-CoA binding motif). For instance, the N-terminal region of *Brassica napus* DGAT1, binding preferentially erucoyl-CoA [54], as well as the DGAT1 from Fig 1 display a low level of identity with EgDGAT1-1 (Fig 8). Unlike them, the four isoforms of *P*. *dactylifera* DGAT1 and *L*. *communis* DGAT1 exhibited a significant sequence identity in the N-terminal region with EgDGAT1-1. These five DGAT1 originating from plants able to store lauric acid are potentially able to use it as acyl donor. Among the conserved features of these sequences we observe a three residue deletion gap (between Glu15 and Pro16 of EgDGAT1-1) and the insertion of a conserved motif (XPDXSSXX) from Val39 to Thr46 of EgDGAT1-1. A three residue motif ^8^ETL^10^ found in EgDGAT1-1 N-terminus is also conserved.

## Discussion

MCFA accumulation is an interesting biotechnological target. The transcriptomic and lipidomic data reported by Dussert *et al*. [25] suggested that oil palm EgDGAT1-1 is a determinant of medium-chain triacylglycerol storage in palm kernel endosperm. In the present study, insights into EgDGAT1-1 activity and localization were obtained for the first time through heterologous expression in a *Y*. *lipolytica* strain defective in neutral lipid accumulation. A comparative study of substrate specificity with AtDGAT1, identified over fifteen years ago as having a major role in seed lipid accumulation [29, 46], was undertaken.

### EgDGAT1-1 encodes a canonical plant DGAT1

The putative EgDGAT1-1 amino acid sequence was identified from an *in silico* analysis of transcriptomics data [25]. We retrieved a completely identical DGAT1-like sequence (XP_010924968) predicted from oil palm whole-genome shotgun sequencing. Thus, the fact that the same amino acid sequence for EgDGAT1-1 was predicted from independent transcriptomic and genomic resources strongly suggest that it is accurate. A multiple sequence alignment with biochemically-characterized plant DGAT1 (Fig 1), including *A*. *thaliana* DGAT1, revealed more than 50% identity with strong conservation of putative transmembrane domains and functional regions. These conserved plant DGAT1 regions were identified in multiple sequence alignments [3, 11, 46] and the importance of several was highlighted through site-directed mutagenesis [45, 55]. A well-conserved leucine zipper motif in plant DGAT1 and absent from animal DGAT1 [6] supports the correct annotation of EgDGAT1-1. In addition, 41 invariant residues highlighted by Cao [48] strongly suggests that the putative EgDGAT1-1 belongs to the DGAT1 family. Taken together these results encouraged us to assess the DGAT function of EgDGAT1-1.

### EgDGAT1-1 restores lipid accumulation in the neutral lipid-defective *Y*. *lipolytica* JMY1877 strain

Until now, the *S*. *cerevisiae* H1246 strain [19] has been the only mutant strain available for exploring DGAT function. The present study is the first example proving that *Y*. *lipolytica* JMY1877 is a valuable tool for studying putative DGATs. Expression of AtDGAT1 or EgDGAT1-1 restored TAG accumulation and significantly increased the yeast total FA content (Figs 2 and 3A). Fluorescence and contrast phase microscopy of both DGAT1 expressing strains stained with Nile red highlighted small cytoplasmic inclusions accumulating neutral lipids (Fig 4A). The lipid composition of the 150,000 *g* floating layer of each DGAT expressing strain was typical of LDs with a high proportion of TAG. Altogether these results demonstrate that EgDGAT1-1 expression is sufficient to restore TAG accumulation in LDs (Fig 4B). The amount of squalene in *Y*. *lipolytica* was not significantly modified by DGAT expression and the presence of LDs (Fig 2). This triterpene was found in LDs in both DGAT1 expressing strains (Fig 4B). The presence of squalene was previously reported in *S*. *cerevisiae* LDs [34, 56] and also in microsomal and mitochondrial membranes when LD biosynthesis was impaired [56].

### Plant DGAT1 show contrasting substrate specificity

In oil palm, EgDGAT1-1 is expressed in the endosperm at the onset of lauric acid accumulation suggesting a preference for MCFAs. Despite the absence of MCFAs in JMY1877 cells grown on YP-glucose (Fig 3B) EgDGAT1-1 was found to be active as it increased total FA content (Fig 3A) and the proportion of long chain oleic acid (Fig 3B). This suggests that the EgDGAT1-1 active site shows plasticity towards various acyl-CoA chain lengths. MCFA accumulation in palm kernel may therefore be driven by the active production of medium-chain acyl-CoA. According to Dussert *et al*. [25] *EgDGAT1-1* is highly expressed in the endosperm and embryo. The endosperm mainly accumulates MCFA but the embryo stores saturated and unsaturated long chain FAs. These results are consistent with the hypothesis of EgDGAT1-1 active site plasticity.

In order to assess the affinity of EgDGAT1-1 for MCFA, we took advantage of *Y*. *lipolytica*’s ability to import lipids from the growth medium and use them as substrates [50, 57]. Each yeast strain was cultured in LAME supplemented medium. Previously, a stereospecific analysis of TAG produced by AtDGAT1 mutant (AS11) seeds revealed a marked reduction in C18:1 and C20:1 incorporation in the *sn*-3 position compared to wild-type [29] suggesting preference of the enzyme for long and very long chain FAs. A high-resolution mass spectrometry analysis of TAG composition was undertaken to precisely determine the esterified FA chain length and degree of unsaturation. When cultured in LAME supplemented medium, the EgDGAT1-1 expressing strain accumulates around twice as much medium-chain C44 and C46 in TAG compared to the AtDGAT1 expressing strain (Fig 6). These results strongly suggest that EgDGAT1-1 has a marked preference for MCFA. Together with the previous (Dussert *et al*. [25]) transcriptome analysis, our results show that EgDGAT1-1 can accommodate various chain lengths and degrees of unsaturation in its active site but retains a marked substrate specificity toward MCFAs.

### The microsomal proteome containing EgDGAT1-1 is conserved upon restoration of TAG synthesis

EgDGAT1-1 is expressed in an active form in *Y*. *lipolytica*, as shown by the restoration of TAG accumulation and LD biogenesis in the JMY1877 strain. DGAT1 are described as ER integral membrane proteins [10, 11]. Proteomics analysis led to non-ambiguous identification of EgDGAT1-1 in the microsomal fraction of the EgDGAT1-1 expressing strain confirming previous reports on DGAT1 localization. To date, the only report of proteomics analysis of yeast microsomal fractions by Klug *et al*. [51] identified 294 proteins in *Pichia pastoris*. In this study we identified and classified 744 microsomal proteins present in the three biological replicates of both strains (Tables A-L in S1 Table). The major functional class (26%) was related to translation and protein folding (Fig 7). Ten of the 15 most abundant proteins found in the control strain belonged to this class. Among them six are homologs of proteins identified in *P*. *pastoris* microsomes (Table 1). Klug *et al*. [51] classified *P*. *pastoris* microsomal proteins in categories related to their localization and function. Ninety-five proteins (32%) were associated with ribosome/translation and chaperone categories. Both studies identified proteins related to lipid metabolism (7% in *Y*. *lipolytica* and 12% in *P*. *pastoris*). These two categories represented at least a third of total proteins. Overall the findings of both studies are consistent with the ER playing an important role in protein synthesis and lipid metabolism. Expression of EgDGAT1-1 did not modify the overall microsomal proteome. Indeed, the *Y*. *lipolytica* proteins, which were overexpressed following EgDGAT1-1 expression, belonged to at least six different functional classes (Table 2). This approach allowed us to gain new insight into the microsomal proteome of oleaginous yeast suggesting that TAG synthesis is decoupled from the level of proteins involved in lipid metabolism.

### The variability of the N-terminal region could be the clue to the diverse substrate specificities found in plant DGAT1

Results obtained upon multiple sequence alignment highlighted a good conservation of the N-terminal region of several DGAT1 expressed in plants storing lauric acid (Fig 8) suggesting that molecular determinants of EgDGAT1-1 substrate specificity could reside in the protein N-terminus. This hypothesis is strengthened by a previous study [54] highlighting that the N-terminal fragment of oilseed rape DGAT1 (first 116 residues of the sequence displayed on Fig 8) preferentially binds erucoyl-CoA, an acyl-CoA derived from a FA highly abundant in original *B*. *napus* cultivars. This N-terminal fragment harbors both the highly variable N-terminal region and the acyl-CoA binding motif described by Jako *et al*. [3] and Xu *et al*. [45]. As the acyl-CoA binding motif is conserved in plant DGAT1 this result suggests that the variability of the N-terminal region could be a clue to the diverse substrate specificity observed among DGAT1 proteins [3, 54, 55]. Similar results were obtained with murine DGAT1 [58]. The N-terminal fragment is directly involved in acyl-CoA binding and selection. However, performing a thorough biochemical characterization of *P*. *dactylifera* and *L*. *communis* DGAT1 in order to assess their affinity for lauric acid as well as site-directed mutagenesis of conserved features will be necessary to determine if the protein N-terminus is involved in acyl-coA selection. A previous study of *T*. *majus* DGAT1 conserved features also described the importance of the FA binding protein signature [45] (Figs 1 and 8). This motif is also found to be critical in closely related acyl-CoA: cholesterol acyltransferase [35, 59], another family of enzyme using acyl-CoA as an acyl donor to mediate the production of sterol esters suggesting a role of this motif in acyl-CoA binding and/or processing. These results are strengthened by a recent study on bovine DGAT1 [60]. A synthetic peptide corresponding to the predicted FA binding protein signature of bovine DGAT1 was shown to have the ability to bind specifically the acyl chain of oleoyl-CoA. We hypothesized that a three-dimension region gathering the FA binding protein signature with an extended N-terminal region including the acyl-CoA binding motif (residues 1–126 of EgDGAT1-1) could be involved in acyl-CoA binding and selection.

DGAT specificity is poorly characterized despite its interest in various fields, including the modification of seed oil composition for the production of industrial and nutritional feedstocks [61]. *Y*. *lipolytica* can accumulate upwards of 90% (w/w) lipid content [62]. It is able to grow on various FAs and their derivatives [50] and to incorporate them into TAG. Our results provide strong evidence that EgDGAT1-1 is involved in lauric acid accumulation in palm kernel oil. A synthetic biology approach combining expression of FA synthesis and incorporation enzymes with dedicated substrate specificities in oleaginous microorganisms will constitute a platform for high-value oil production.

## Supporting Information

S1 Table List of the 625 proteins identified in the three transformants of each strain (control strain and EgDGAT1-1 expressing strain) and manually curated in 12 classes. Proteins from three independent transformants of *Y*. *lipolytica* 100,000 g microsomes were digested in gel. Peptides were separated by liquid chromatography and analyzed with a LTQ Orbitrap mass spectrometer using a nano-electrospray interface. Proteins found in the three transformants of each strain were manually curated in 12 classes (Tables A-L in S1 Table) and ranked according to their mean PAI in the control strain.
(PDF)

## References

[1] Graham IA. Seed storage oil mobilization. Annu Rev Plant Biol. 2008;59:115–42. 10.1146/annurev.arplant.59.032607.092938 18444898

[2] Zweytick D, Athenstaedt K, Daum G. Intracellular lipid particles of eukaryotic cells. Biochim Biophys Acta. 2000 9 18;1469(2):101–20. 10998572 10.1016/s0005-2736(00)00294-7

[3] Jako C, Kumar A, Wei Y, Zou J, Barton DL, Giblin EM, et al Seed-specific over-expression of an Arabidopsis cDNA encoding a diacylglycerol acyltransferase enhances seed oil content and seed weight. Plant Physiol. 2001 6;126(2):861–74. 11402213 10.1104/pp.126.2.861 PMC111175

[4] Yen CLE, Stone SJ, Koliwad S, Harris C, Farese RV. DGAT enzymes and triacylglycerol biosynthesis. J Lipid Res. 2008 11;49(11):2283–301. 10.1194/jlr.R800018-JLR200 18757836 PMC3837458

[5] Beopoulos A, Haddouche R, Kabran P, Dulermo T, Chardot T, Nicaud JM. Identification and characterization of DGA2, an acyltransferase of the DGAT1 acyl-CoA:diacylglycerol acyltransferase family in the oleaginous yeast Yarrowia lipolytica. New insights into the storage lipid metabolism of oleaginous yeasts. Appl Microbiol Biotechnol. 2012 2;93(4):1523–37. 10.1007/s00253-011-3506-x 21808970 PMC3275733

[6] Liu Q, Siloto RM, Lehner R, Stone SJ, Weselake RJ. Acyl-CoA:diacylglycerol acyltransferase: molecular biology, biochemistry and biotechnology. Prog Lipid Res. 2012 10;51(4):350–77. 10.1016/j.plipres.2012.06.001 22705711

[7] Dahlqvist A, Stahl U, Lenman M, Banas A, Lee M, Sandager L, et al Phospholipid:diacylglycerol acyltransferase: an enzyme that catalyzes the acyl-CoA-independent formation of triacylglycerol in yeast and plants. Proc Natl Acad Sci U S A. 2000 6 6;97(12):6487–92. 10829075 10.1073/pnas.120067297 PMC18631

[8] Ståhl U, Carlsson AS, Lenman M, Dahlqvist A, Huang B, Banaś W, et al Cloning and functional characterization of a phospholipid:diacylglycerol acyltransferase from Arabidopsis. Plant Physiol. 2004 7 1;135(3):1324–35. 15247387 10.1104/pp.104.044354 PMC519051

[9] McFie PJ, Banman SL, Kary S, Stone SJ. Murine diacylglycerol acyltransferase-2 (DGAT2) can catalyze triacylglycerol synthesis and promote lipid droplet formation independent of its localization to the endoplasmic reticulum. J Biol Chem. 2011 8;286(32):28235–46. 10.1074/jbc.M111.256008 21680734 PMC3151068

[10] McFie PJ, Stone SL, Banman SL, Stone SJ. Topological orientation of acyl-CoA:diacylglycerol acyltransferase-1 (DGAT1) and identification of a putative active site histidine and the role of the N terminus in dimer/tetramer formation. J Biol Chem. 2010 11;285(48):37377–87. 10.1074/jbc.M110.163691 20876538 PMC2988343

[11] Shockey JM, Gidda SK, Chapital DC, Kuan JC, Dhanoa PK, Bland JM, et al Tung tree DGAT1 and DGAT2 have nonredundant functions in triacylglycerol biosynthesis and are localized to different subdomains of the endoplasmic reticulum. Plant Cell. 2006 9;18(9):2294–313. 16920778 10.1105/tpc.106.043695 PMC1560902

[12] Turchetto-Zolet A, Maraschin F, de Morais G, Cagliari A, Andrade C, Margis-Pinheiro M, et al Evolutionary view of acyl-CoA diacylglycerol acyltransferase (DGAT), a key enzyme in neutral lipid biosynthesis. BMC Evol Biol. 2011;11(1):263. 21933415 10.1186/1471-2148-11-263 PMC3185287

[13] Hofmann K. A superfamily of membrane-bound O-acyltransferases with implications for Wnt signaling. Trends Biochem Sci. 2000;25(3):111–2. 10694878 10.1016/s0968-0004(99)01539-x

[14] Cases S, Smith SJ, Zheng YW, Myers HM, Lear SR, Sande E, et al Identification of a gene encoding an acyl CoA:diacylglycerol acyltransferase, a key enzyme in triacylglycerol synthesis. Proc Natl Acad Sci U S A. 1998 10 27;95(22):13018–23. 9789033 10.1073/pnas.95.22.13018 PMC23692

[15] Cases S, Stone SJ, Zhou P, Yen E, Tow B, Lardizabal KD, et al Cloning of DGAT2, a second mammalian diacylglycerol acyltransferase, and related family members. J Biol Chem. 2001 10;276(42):38870–6. 11481335 10.1074/jbc.M106219200

[16] Smith SJ, Cases S, Jensen DR, Chen HC, Sande E, Tow B, et al Obesity resistance and multiple mechanisms of triglyceride synthesis in mice lacking Dgat. Nat Genet. 2000 5;25(1):87–90. 10802663 10.1038/75651

[17] Chen HC, Smith SJ, Ladha Z, Jensen DR, Ferreira LD, Pulawa LK, et al Increased insulin and leptin sensitivity in mice lacking acyl CoA:diacylglycerol acyltransferase 1. J Clin Invest. 2002;109(8):1049–55. 11956242 10.1172/JCI14672 PMC150948

[18] Stone SJ, Myers HM, Watkins SM, Brown BE, Feingold KR, Elias PM, et al Lipopenia and skin barrier abnormalities in DGAT2-deficient mice. J Biol Chem. 2004 3 19;279(12):11767–76. 14668353 10.1074/jbc.M311000200

[19] Sandager L, Gustavsson MH, Stahl U, Dahlqvist A, Wiberg E, Banas A, et al Storage lipid synthesis is non-essential in yeast. J Biol Chem. 2002 2 22;277(8):6478–82. 11741946 10.1074/jbc.M109109200

[20] Routaboul J-M, Benning C, Bechtold N, Caboche M, Lepiniec L. The TAG1 locus of Arabidopsis encodes for a diacylglycerol acyltransferase. Plant Physiol Biochem. 1999;37(11):831–40. 10580283 10.1016/s0981-9428(99)00115-1

[21] Kroon JTM, Wei W, Simon WJ, Slabas AR. Identification and functional expression of a type 2 acyl-CoA:diacylglycerol acyltransferase (DGAT2) in developing castor bean seeds which has high homology to the major triglyceride biosynthetic enzyme of fungi and animals. Phytochemistry. 2006;67(23):2541–9. 17084870 10.1016/j.phytochem.2006.09.020

[22] Saha S, Enugutti B, Rajakumari S, Rajasekharan R. Cytosolic triacylglycerol biosynthetic pathway in oilseeds. Molecular cloning and expression of Peanut cytosolic diacylglycerol acyltransferase. Plant Physiol. 2006 8;141(4):1533–43. 16798944 10.1104/pp.106.082198 PMC1533943

[23] Hernandez ML, Whitehead L, He Z, Gazda V, Gilday A, Kozhevnikova E, et al A cytosolic acyltransferase contributes to triacylglycerol synthesis in sucrose-rescued Arabidopsis seed oil catabolism mutants. Plant Physiol. 2012 9;160(1):215–25. 10.1104/pp.112.201541 22760209 PMC3440200

[24] Durrett TP, McClosky DD, Tumaney AW, Elzinga DA, Ohlrogge J, Pollard M. A distinct DGAT with sn-3 acetyltransferase activity that synthesizes unusual, reduced-viscosity oils in Euonymus and transgenic seeds. Proc Natl Acad Sci U S A. 2010 5;107(20):9464–9. 10.1073/pnas.1001707107 20439724 PMC2889089

[25] Dussert S, Guerin C, Andersson M, Joet T, Tranbarger TJ, Pizot M, et al Comparative transcriptome analysis of three oil palm fruit and seed tissues that differ in oil content and fatty acid composition. Plant Physiol. 2013 7;162(3):1337–58. 10.1104/pp.113.220525 23735505 PMC3707537

[26] Grisart B, Farnir F, Karim L, Cambisano N, Kim J-J, Kvasz A, et al Genetic and functional confirmation of the causality of the DGAT1 K232A quantitative trait nucleotide in affecting milk yield and composition. Proc Natl Acad Sci U S A. 2004 2 24;101(8):2398–403. 14983021 10.1073/pnas.0308518100 PMC356962

[27] Weselake RJ, Shah S, Tang MG, Quant PA, Snyder CL, Furukawa-Stoffer TL, et al Metabolic control analysis is helpful for informed genetic manipulation of oilseed rape (Brassica napus) to increase seed oil content. J Exp Bot. 2008 10;59(13):3543–9. 10.1093/jxb/ern206 18703491 PMC2561151

[28] Liang MH, Jiang JG. Advancing oleaginous microorganisms to produce lipid via metabolic engineering technology. Prog Lipid Res. 2013 10;52(4):395–408. 10.1016/j.plipres.2013.05.002 23685199

[29] Katavic V, Reed DW, Taylor DC, Giblin EM, Barton DL, Zou J, et al Alteration of seed fatty acid composition by an ethyl methanesulfonate-induced mutation in Arabidopsis thaliana affecting diacylglycerol acyltransferase activity. Plant Physiol. 1995 5 1;108(1):399–409. 7784510 10.1104/pp.108.1.399 PMC157347

[30] Hall TA. BioEdit: a user-friendly biological sequence alignment editor and analysis program for Windows 95/98/NT. Nucl Acids Symp Ser. 1999;41:95–8.

[31] Sievers F, Wilm A, Dineen D, Gibson TJ, Karplus K, Li W, et al Fast, scalable generation of high-quality protein multiple sequence alignments using Clustal Omega. Mol Syst Biol. 2011;7:539 10.1038/msb.2011.75 21988835 PMC3261699

[32] Hofmann K, Stoffel W. TMbase—A database of membrane spanning proteins segments. Biol Chem Hoppe-Seyler. 1993;374:166.

[33] Barth G, Beckerich J-M, Dominguez A, Kerscher S, Ogrydziak D, Titorenko V, et al Functional genetics of Yarrowia lipolytica In: de Winde J, editor. Functional Genetics of Industrial Yeasts: Springer Berlin Heidelberg; 2003 p. 227–71.

[34] Aymé L, Baud S, Dubreucq B, Joffre F, Chardot T. Function and localization of the Arabidopsis thaliana diacylglycerol acyltransferase DGAT2 expressed in yeast. Plos One. 2014;9(3):e92237 10.1371/journal.pone.0092237 24663078 PMC3963872

[35] Bouvier-Navé P, Benveniste P, Oelkers P, Sturley SL, Schaller H. Expression in yeast and tobacco of plant cDNAs encoding acyl CoA: diacylglycerol acyltransferase. Eur J Biochem. 2000 1;267(1):85–96. 10601854 10.1046/j.1432-1327.2000.00961.x

[36] Neuhoff V, Arold N, Taube D, Ehrhardt W. Improved staining of proteins in polyacrylamide gels including isoelectric focusing gels with clear background at nanogram sensitivity using Coomassie Brilliant Blue G-250 and R-250. Electrophoresis. 1988 6;9(6):255–62. 2466658 10.1002/elps.1150090603

[37] Abdallah C, Valot B, Guillier C, Mounier A, Balliau T, Zivy M, et al The membrane proteome of Medicago truncatula roots displays qualitative and quantitative changes in response to arbuscular mycorrhizal symbiosis. Journal of proteomics. 2014 8 28;108:354–68. 10.1016/j.jprot.2014.05.028 24925269

[38] Blein-Nicolas M, Albertin W, Valot B, Marullo P, Sicard D, Giraud C, et al Yeast proteome variations reveal different adaptive responses to grape must fermentation. Mol Biol Evol. 2013 6;30(6):1368–83. 10.1093/molbev/mst050 23493259

[39] Sherman DJ, Martin T, Nikolski M, Cayla C, Souciet JL, Durrens P. Genolevures: protein families and synteny among complete hemiascomycetous yeast proteomes and genomes. Nucleic Acids Res. 2009 1;37(Database issue):D550–4. 10.1093/nar/gkn859 19015150 PMC2686504

[40] Folch J, Lees M, Sloane Stanley GH. A simple method for the isolation and purification of total lipides from animal tissues. J Biol Chem. 1957 5;226(1):497–509. 13428781

[41] Browse J, McCourt PJ, Somerville CR. Fatty acid composition of leaf lipids determined after combined digestion and fatty acid methyl ester formation from fresh tissue. Anal Biochem. 1986 1;152(1):141–5. 3954036 10.1016/0003-2697(86)90132-6

[42] Froissard M, D'Andrea S, Boulard C, Chardot T. Heterologous expression of AtClo1, a plant oil body protein, induces lipid accumulation in yeast. FEMS Yeast Res. 2009 5;9(3):428–38. 10.1111/j.1567-1364.2009.00483.x 19220478

[43] Gallart-Ayala H, Courant F, Severe S, Antignac JP, Morio F, Abadie J, et al Versatile lipid profiling by liquid chromatography-high resolution mass spectrometry using all ion fragmentation and polarity switching. Preliminary application for serum samples phenotyping related to canine mammary cancer. Anal Chim Acta. 2013 9 24;796:75–83. 10.1016/j.aca.2013.08.006 24016586

[44] Pan X, Siloto RM, Wickramarathna AD, Mietkiewska E, Weselake RJ. Identification of a pair of phospholipid:diacylglycerol acyltransferases from developing flax (Linum usitatissimum L.) seed catalyzing the selective production of trilinolenin. J Biol Chem. 2013 8 16;288(33):24173–88. 10.1074/jbc.M113.475699 23824186 PMC3745363

[45] Xu JY, Francis T, Mietkiewska E, Giblin EM, Barton DL, Zhang Y, et al Cloning and characterization of an acyl-CoA-dependent diacylglycerol acyltransferase 1 (DGAT1) gene from Tropaeolum majus, and a study of the functional motifs of the DGAT protein using site-directed mutagenesis to modify enzyme activity and oil content. Plant Biotechnol J. 2008 10;6(8):799–818. 10.1111/j.1467-7652.2008.00358.x 18631243

[46] Zou JT, Wei YD, Jako C, Kumar A, Selvaraj G, Taylor DC. The Arabidopsis thaliana TAG1 mutant has a mutation in a diacylglycerol acyltransferase gene. Plant J. 1999 9;19(6):645–53. 10571850 10.1046/j.1365-313x.1999.00555.x

[47] Hobbs DH, Lu C, Hills MJ. Cloning of a cDNA encoding diacylglycerol acyltransferase from Arabidopsis thaliana and its functional expression. FEBS Lett. 1999;452(3):145–9. 10386579 10.1016/s0014-5793(99)00646-8

[48] Cao H. Structure-function analysis of diacylglycerol acyltransferase sequences from 70 organisms. BMC research notes. 2011;4:249 10.1186/1756-0500-4-249 21777418 PMC3157451

[49] Zhang M, Fan JL, Taylor DC, Ohlrogge JB. DGAT1 and PDAT1 acyltransferases have overlapping functions in Arabidopsis triacylglycerol biosynthesis and are essential for normal pollen and seed development. Plant Cell. 2009 12;21(12):3885–901. 10.1105/tpc.109.071795 20040537 PMC2814504

[50] Papanikolaou S, Chevalot I, Komaitis M, Marc I, Aggelis G. Single cell oil production by Yarrowia lipolytica growing on an industrial derivative of animal fat in batch cultures. Appl Microbiol Biotechnol. 2002 3;58(3):308–12. 11935181 10.1007/s00253-001-0897-0

[51] Klug L, Tarazona P, Gruber C, Grillitsch K, Gasser B, Trotzmuller M, et al The lipidome and proteome of microsomes from the methylotrophic yeast Pichia pastoris. Biochim Biophys Acta. 2014 2;1841(2):215–26. 24246743 10.1016/j.bbalip.2013.11.005

[52] Dong S, Huang J, Li Y, Zhang J, Lin S, Zhang Z. Cloning, characterization, and expression analysis of acyl-acyl carrier protein (ACP)-thioesterase B from seeds of Chinese Spicehush (Lindera communis). Gene. 2014 5 25;542(1):16–22. 10.1016/j.gene.2014.03.028 24631366

[53] Devshony S, Eteshola E, Shani A. Characteristics and some potential applications of date palm (Phoenix dactylifera L.) seeds and seed oil. J Am Oil Chem Soc. 1992 6;69(6):595–7.

[54] Weselake RJ, Madhavji M, Szarka SJ, Patterson NA, Wiehler WB, Nykiforuk CL, et al Acyl-CoA-binding and self-associating properties of a recombinant 13.3 kDa N-terminal fragment of diacylglycerol acyltransferase-1 from oilseed rape. BMC Biochem. 2006;7:24 17192193 10.1186/1471-2091-7-24 PMC1764880

[55] Manas-Fernandez A, Vilches-Ferron M, Garrido-Cardenas JA, Belarbi EH, Alonso DL, Garcia-Maroto F. Cloning and molecular characterization of the Acyl-CoA:Diacylglycerol Acyltransferase 1 (DGAT1) gene from Echium. Lipids. 2009 6;44(6):555–68. 10.1007/s11745-009-3303-9 19412626

[56] Spanova M, Czabany T, Zellnig GN, Leitner E, Hapala I, Daum GN. Effect of lipid particle biogenesis on the subcellular distribution of squalene in the yeast Saccharomyces cerevisiae. J Biol Chem. 2010 2 26;285(9):6127–33. 10.1074/jbc.M109.074229 20032462 PMC2825407

[57] Mlickova K, Roux E, Athenstaedt K, d'Andrea S, Daum G, Chardot T, et al Lipid accumulation, lipid body formation, and acyl coenzyme A oxidases of the yeast Yarrowia lipolytica. Appl Environ Microbiol. 2004 7;70(7):3918–24. 15240264 10.1128/AEM.70.7.3918-3924.2004 PMC444788

[58] Siloto RM, Madhavji M, Wiehler WB, Burton TL, Boora PS, Laroche A, et al An N-terminal fragment of mouse DGAT1 binds different acyl-CoAs with varying affinity. Biochem Biophys Res Commun. 2008 8 29;373(3):350–4. 10.1016/j.bbrc.2008.06.031 18571500

[59] Guo Z, Cromley D, Billheimer JT, Sturley SL. Identification of potential substrate-binding sites in yeast and human acyl-CoA sterol acyltransferases by mutagenesis of conserved sequences. J Lipid Res. 2001 8;42(8):1282–91. 11483630

[60] Lopes JL, Nobre TM, Cilli EM, Beltramini LM, Araujo AP, Wallace BA. Deconstructing the DGAT1 enzyme: Binding sites and substrate interactions. Biochim Biophys Acta. 2014 12;1838(12):3145–52. 10.1016/j.bbamem.2014.08.017 25152299

[61] Metzger JO. Fats and oils as renewable feedstock for chemistry. Eur J Lipid Sci Tech. 2009 9;111(9):865–76.

[62] Blazeck J, Hill A, Liu L, Knight R, Miller J, Pan A, et al Harnessing Yarrowia lipolytica lipogenesis to create a platform for lipid and biofuel production. Nat Commun. 2014;5:3131 10.1038/ncomms4131 24445655

