# Supplementary material for: Molecular Characterization of the Elaeis guineensis Medium-Chain Fatty Acid Diacylglycerol Acyltransferase DGAT1-1 by Heterologous Expression in Yarrowia lipolytica
Source: PLoS One. 2015 Nov 18;10(11):e0143113. doi: 10.1371/journal.pone.0143113 (PMC4651311; doi:10.1371/journal.pone.0143113)
Supplement: S1 Table — Proteins from three independent transformants of Y. lipolytica 100,000 g microsomes were digested in gel. Peptides were separated by liquid chromatography and analyzed with a LTQ Orbitrap mass spectrometer using a nano-electrospray interface. Proteins found in the three transformants of each strain were manually curated in 12 classes (Tables A-L in S1 Table) and ranked according to their mean PAI in the control strain. (PDF) [file pone.0143113.s001.pdf]

S1 TABLE. List of the 625 proteins identified in the three transformants of each strain (control strain and EgDGAT1-1 expressing strain) and manually curated in 12 classes.

Proteins from three independent transformants of Y. lipolytica 100,000 g microsomes were digested in gel. Peptides were separated by liquid chromatography and analyzed with a LTQ Orbitrap mass spectrometer using a nano-electrospray interface. Proteins found in the three transformants of each strain were manually curated in 12 classes (S1A Table to S1L Table) and ranked according to their mean PAI in the control strain.

S1A TABLE. Translation and protein folding class

Gene ID	Mean PAI per control replicate	Mean PAI per EgDGAT1-1 replicate	Protein description in Génolevures database
YALI0C09141g	9.86	10.90	uniprot|O59949 Yarrowia lipolytica Elongation factor 1-alpha (EF-1-alpha)
YALI0F25289g	5.63	6.56	highly similar to uniprot|P22202 Saccharomyces cerevisiae YER103w SSA4 heat shock protein of HSP70 family
YALI0C07953g	5.54	5.40	highly similar to uniprot|P46598 Candida albicans CaHSC82 Heat shock protein 90 homolog
YALI0D08184g	5.53	6.67	highly similar to uniprot|P22202 Saccharomyces cerevisiae YER103w SSA4 heat shock protein of HSP70 family cytosolic
YALI0E13706g	5.14	3.20	uniprot|Q99170 Yarrowia lipolytica dnaK-type molecular chaperone involved in ER translocation of secretory proteins (BiP homologue)
YALI0D22352g	4.92	6.99	highly similar to uniprot|P22202 Saccharomyces cerevisiae YER103w SSA4 heat shock protein of HSP70 family cytosolic
YALI0C17347g	4.74	4.82	highly similar to uniprot|P12398 Saccharomyces cerevisiae YJR045c SSC1 Heat shock protein 70-related protein SSC1 mitochondrial precursor (Endonuclease SCEI 75 kDa subunit)
YALI0A00352g	4.67	5.46	highly similar to uniprot|P32324 Saccharomyces cerevisiae YOR133w EFT1 and highly similar to uniprot|P32324 Saccharomyces cerevisiae YDR385W translation elongation factor eEF2
YALI0E35046g	4.42	5.33	highly similar to uniprot|P22202 Saccharomyces cerevisiae YER103w SSA4 heat shock protein of HSP70 family cytosolic P14.1.f13.1
YALI0E13277g	4.06	3.60	uniprot|O93814 Yarrowia lipolytica Elongation factor 3 (EF-3)
YALI0E13255g	3.25	3.71	similar to uniprot|P32589 Saccharomyces cerevisiae YPL106c SSE1 heat shock protein of HSP70 family
YALI0A00132g	2.83	3.53	highly similar to uniprot|P40150 Saccharomyces cerevisiae YNL209w SSB2 heat shock protein of HSP70 family cytosolic
YALI0E27962g	2.57	3.77	highly similar to uniprot|P31539 Saccharomyces cerevisiae YLL026w HSP104 heat shock protein
YALI0F09790g	2.44	2.78	highly similar to uniprot|P05759 Saccharomyces cerevisiae YLR167W RPS31 Fusion protein that is cleaved to yield ubiquitin and a ribosomal protein of the small subunit
YALI0F08745g	2.28	2.61	highly similar to uniprot|P61864 Saccharomyces cerevisiae YKR094c RPL40B and highly similar to uniprot|P61864 Saccharomyces cerevisiae YIL148W RPL40A Fusion protein that is cleaved to yield ubiquitin

			and a ribosomal protein of the large subunit
YALI0F14465g	2.20	2.07	highly similar to uniprot|P14127 Saccharomyces cerevisiae YDR447c RP51B ribosomal protein S17.e.B or uniprot|P02407 Saccharomyces cerevisiae YML024w RP51A ribosomal protein S17.e.A
YALI0C05082g	2.11	1.56	similar to uniprot|P40525 Saccharomyces cerevisiae YIL052c RPL34B ribosomal protein L34.e
YALI0B08866g	1.87	1.80	similar to uniprot|P07279 Saccharomyces cerevisiae YNL301C 60S ribosomal protein L18 (RP28)
YALI0C03872g	1.83	1.83	highly similar to uniprot|P07282 Saccharomyces cerevisiae YLR333c RPS31B ribosomal protein
YALI0D20614g	1.83	1.92	highly similar to uniprot|Q8ISP0 Branchiostoma belcheri Ribosomal protein S18
YALI0F24739g	1.71	1.67	highly similar to uniprot|P05736 Saccharomyces cerevisiae YIL018w RPL5A 60S large subunit ribosomal protein L8.e
YALI0F12463g	1.69	1.93	similar to uniprot|P33416 Saccharomyces cerevisiae YDR258c
YALI0C06820g	1.67	1.91	highly similar to uniprot|P49626 Saccharomyces cerevisiae YDR012W 60S ribosomal protein L4-B (L2B) (RP2)
YALI0E19701g	1.67	1.56	highly similar to uniprot|P39938 Saccharomyces cerevisiae YGL189c RPS26A 40S small subunit ribosomal protein S26e.c7 or uniprot|P39939 Saccharomyces cerevisiae YER131w RPS26B 40S small subunit ribosomal protein S26e- c5
YALI0E25025g	1.61	1.56	similar to uniprot|P05735 Saccharomyces cerevisiae YBR084C-A RPL19A and similar to uniprot|P05735 Saccharomyces cerevisiae YBL027W RPL19B Ribosomal Protein of the Large subunit
YALI0C15895g	1.61	1.44	highly similar to uniprot|P46990 Saccharomyces cerevisiae YJL177w RPL20B 60s large subunit ribosomal protein L17.e P2.300.f2.1
YALI0E13618g	1.58	1.39	highly similar to uniprot|O60143 Schizosaccharomyces pombe 60S ribosomal protein L7-C
YALI0F02805g	1.56	2.04	highly similar to uniprot|P19882 Saccharomyces cerevisiae YLR259c HSP60 heat shock protein - chaperone mitochondrial
YALI0D13728g	1.53	1.53	similar to uniprot|P26782 Saccharomyces cerevisiae YIL069c RP50B 40S small subunit ribosomal protein S24.e
YALI0F24695g	1.48	1.63	highly similar to uniprot|P29453 Saccharomyces cerevisiae YLL045c RPL4B 60s large subunit ribosomal protein L7a.e.B alternative splicing generates different translational starts
YALI0E22352g	1.45	1.33	highly similar to uniprot|P26321 Saccharomyces cerevisiae YPL131w RPL1 60S large subunit ribosomal protein L5.e
YALI0E20031g	1.44	1.39	similar to uniprot|P38011 Saccharomyces cerevisiae YMR116c ASC1 40S small subunit ribosomal protein

YALI0D17116g	1.42	1.17	highly similar to uniprot|Q12460 Saccharomyces cerevisiae YLR197w SIK1 involved in pre-rRNA processing
YALI0B04180g	1.40	1.43	similar to uniprot|P40825 Saccharomyces cerevisiae YOR335C Alanyl-tRNA synthetase cytoplasmic (EC 6.1.1.7) (Alanine--tRNA ligase)
YALI0D12903g	1.39	1.14	uniprot|O59950 Yarrowia lipolytica 40S ribosomal protein S4 (S7)
YALI0E00550g	1.38	1.19	similar to uniprot|P36105 Saccharomyces cerevisiae YKL006w RPL14A ribosomal protein or uniprot|P38754 Saccharomyces cerevisiae YHL001w RPL14B ribosomal protein
YALI0E21219g	1.38	1.05	similar to uniprot|P53221 Saccharomyces cerevisiae YGR034W 60S ribosomal protein L26-B (YL33)
YALI0E29073g	1.38	1.25	highly similar to uniprot|P51401 Saccharomyces cerevisiae YNL067W RPL9B 60S ribosomal protein L9-B
YALI0B12848g	1.37	1.30	highly similar to uniprot|P40213 Saccharomyces cerevisiae YMR143w RPS16A ribosomal protein or uniprot|P40213 Saccharomyces cerevisiae YDL083c RPS16B ribosomal protein
YALI0F05676g	1.36	1.38	highly similar to uniprot|P23248 Saccharomyces cerevisiae YML063w or uniprot|P33442 Saccharomyces cerevisiae YLR441c
YALI0D05753g	1.33	1.14	highly similar to wi|NCU07830.1 Neurospora crassa NCU07830.1 40S ribosomal protein S14 (CRP2)
YALI0B05896g	1.33	2.33	similar to uniprot|P0C2H6 Saccharomyces cerevisiae YHR010w RPL27A and similar to uniprot|P0C2H7 Saccharomyces cerevisiae YDR471W RPL27B Ribosomal Protein of the Large subunit
YALI0D05731g	1.33	0.83	highly similar to uniprot|P0C0W1 Saccharomyces cerevisiae YJL190c RPS22A and highly similar to uniprot|Q3E7Y3 Saccharomyces cerevisiae YLR367W RPS22B Ribosomal Protein of the Small subunit
YALI0E20581g	1.33	1.33	similar to uniprot|P38701 Saccharomyces cerevisiae YHL015w URP2 ribosomal protein
YALI0F25531g	1.33	1.53	highly similar to uniprot|P08792 Pichia jadinii 60S ribosomal protein L25
YALI0E34826g	1.33	1.22	similar to uniprot|P38711 Saccharomyces cerevisiae YHR021c R27B 40S ribosomal protein S27-B
YALI0F20482g	1.33	1.33	highly similar to uniprot|P26781 Saccharomyces cerevisiae YDR025w RPS11A and RPS11B ribosomal protein S11. e or uniprot|P26781 Saccharomyces cerevisiae YBR048w RPS11B ribosomal protein S11.e.B
YALI0D13104g	1.30	1.04	similar to uniprot|P41805 Saccharomyces cerevisiae YLR075w GRC5 60S large subunit ribosomal protein
YALI0A18205g	1.29	1.46	similar to uniprot|P32905 Saccharomyces cerevisiae YGR214w NAB1A 40S ribosomal protein p40 homolog A
YALI0E27830g	1.29	1.25	similar to uniprot|Q02326 Saccharomyces cerevisiae YML073c YL16A 60S large subunit ribosomal protein
YALI0F18766g	1.29	1.48	highly similar to uniprot|P02365 Saccharomyces

			cerevisiae YPL090c RPS10B ribosomal protein S6.e and uniprot|P02365 Saccharomyces cerevisiae YBR181c RPS101 ribosomal protein S6.e
YALI0B12826g	1.28	1.28	similar to uniprot|Q12690 Saccharomyces cerevisiae YDL082w RPL13A 60S large subunit ribosomal protein L13 or uniprot|P40212 Saccharomyces cerevisiae YMR142c RPL13B 60S large subunit ribosomal protein
YALI0C21560g	1.26	1.28	highly similar to uniprot|P14126 Saccharomyces cerevisiae YOR063w TCM1 60S large subunit ribosomal protein L3.e
YALI0B00946g	1.25	1.10	highly similar to uniprot|Q12499 Saccharomyces cerevisiae YOR310C Nucleolar protein NOP58 (Nucleolar protein NOP5)
YALI0C09218g	1.22	1.26	similar to uniprot|P26784 Saccharomyces cerevisiae YIL133C 60S ribosomal protein L16-A (L13A) (RP22)
YALI0E23694g	1.18	1.08	some similarities with uniprot|P05750 Saccharomyces cerevisiae YNL178w RPS3 ribosomal protein S3
YALI0E14597g	1.18	1.29	similar to uniprot|P38088 Saccharomyces cerevisiae YBR121c GRS1 glycine--tRNA ligase
YALI0C05148g	1.17	1.00	highly similar to uniprot|Q10421 Schizosaccharomyces pombe 40S ribosomal protein S28 (S33)
YALI0D24387g	1.17	1.00	highly similar to uniprot|P54780 Saccharomyces cerevisiae YMR121C 60S ribosomal protein L15-B (YL10) (L13) (RP15R) (YP18)
YALI0E32208g	1.17	0.67	similar to uniprot|P05749 Saccharomyces cerevisiae YLR061W 60S ribosomal protein L22-A (YL31) (RP4)
YALI0F24123g	1.17	1.27	highly similar to uniprot|P47913 Saccharomyces cerevisiae YOR312c RPL18B 60S large subunit ribosomal protein and highly similar to uniprot|P47913 Saccharomyces cerevisiae YMR242c RPL18A 60s large subunit ribosomal protein
YALI0E28468g	1.16	1.25	similar to uniprot|P46655 Saccharomyces cerevisiae YGL245w Glutamyl-tRNA synthetase cytoplasmic
YALI0B12562g	1.15	1.40	similar to uniprot|P36008 Saccharomyces cerevisiae YKL081w TEF4 translation elongation factor eEF1 gamma chain
YALI0F18942g	1.14	0.97	similar to uniprot|P38249 Saccharomyces cerevisiae YBR079c RPG1 translation initiation factor eIF3 (p110 subunit)
YALI0E14465g	1.13	0.96	similar to uniprot|P25443 Saccharomyces cerevisiae YGL123w SUP44 40S small subunit ribosomal protein
YALI0B20922g	1.10	1.37	highly similar to uniprot|P47943 Schizosaccharomyces pombe Eukaryotic initiation factor 4A (eIF-4A) (eIF4A)
YALI0E24607g	1.08	1.38	similar to uniprot|P26637 Saccharomyces cerevisiae YPL160w CDC60 leucine--tRNA ligase cytosolic
YALI0F05522g	1.06	0.89	highly similar to uniprot|Q12672 Saccharomyces cerevisiae YPL079w URP1B ribosomal or uniprot|Q02753 Saccharomyces cerevisiae YBR191w URP1A ribosomal protein L21
YALI0F06160g	1.06	0.83	highly similar to uniprot|P48589 Saccharomyces

			cerevisiae YOR369c
YALI0A10725g	1.05	0.95	similar to uniprot|P26786 Saccharomyces cerevisiae YOR096W 40S ribosomal protein S7-A (RP30)
YALI0E31911g	1.04	0.89	highly similar to uniprot|P53030 Saccharomyces cerevisiae YPL220w RPL1A and highly similar to uniprot|P53030 Saccharomyces cerevisiae YGL135W RPL1B Ribosomal Protein of the Large subunit
YALI0F24387g	1.04	1.04	highly similar to uniprot|P02992 Saccharomyces cerevisiae YOR187w TUF1 translation elongation factor TU mitochondrial
YALI0F08569g	1.03	1.10	similar to uniprot|P26783 Saccharomyces cerevisiae YJR123w RPS5 ribosomal protein S5
YALI0F16291g	1.03	1.36	highly similar to uniprot|P15180 Saccharomyces cerevisiae YDR037w KRS1 lysyl-tRNA synthetase cytosolic
YALI0F11231g	1.02	1.18	similar to uniprot|P04801 Saccharomyces cerevisiae YIL078w THS1 threonyl tRNA synthetase
YALI0F20218g	1.02	1.34	similar to uniprot|P07806 Saccharomyces cerevisiae YGR094w VAS1 valyl-tRNA synthetase
YALI0E23562g	1.00	1.08	similar to uniprot|P38664 Kluyveromyces lactis RL30_KLULA 60S ribosomal protein L30 (L32)
YALI0E30602g	1.00	1.00	similar to uniprot|O14455 Saccharomyces cerevisiae YPL249C-A R36B 60S ribosomal protein L36-B (L39B)
YALI0B12474g	0.99	1.33	similar to uniprot|P38788 Saccharomyces cerevisiae YHR064c PDR13 regulator protein involved in pleiotropic drug resistance
YALI0E27071g	0.94	0.61	similar to uniprot|P35691 Saccharomyces cerevisiae YKL056c strong similarity to human IgE-dependent histamine- releasing factor
YALI0E23584g	0.93	0.67	highly similar to uniprot|P38665 Kluyveromyces lactis RL24_KLULA 60S ribosomal protein L24 (L30)
YALI0C08987g	0.91	0.96	similar to uniprot|P15705 Saccharomyces cerevisiae YOR027W Heat shock protein STI1
YALI0F09669g	0.90	0.95	highly similar to uniprot|P32471 Saccharomyces cerevisiae YAL003w EFB1 translation elongation factor eEF1beta
YALI0C15873g	0.89	0.86	highly similar to uniprot|P15646 Saccharomyces cerevisiae YDL014w NOP1 fibrillarin
YALI0C24420g	0.87	1.03	similar to uniprot|P29547 Saccharomyces cerevisiae YPL048w CAM1 translation elongation factor eEF1 gamma homologue
YALI0E30811g	0.83	0.75	highly similar to uniprot|P38061 Saccharomyces cerevisiae YBL092w RPL32 60S large subunit ribosomal protein L32.e
YALI0E34573g	0.83	1.00	highly similar to uniprot|P49631 Saccharomyces cerevisiae YPR043w RPL43A and highly similar to uniprot|P49631 Saccharomyces cerevisiae YJR094W-A RPL43B Ribosomal Protein of the Large subunit
YALI0F11055g	0.81	0.74	highly similar to uniprot|P05756 Saccharomyces cerevisiae YDR064w YS15 ribosomal protein

YALI0F24959g	0.79	0.96	similar to uniprot|P05754 Saccharomyces cerevisiae YER102w RPS8B and similar to uniprot|P05754 Saccharomyces cerevisiae YBL072c RPS8A Ribosomal Protein of the Small subunit
YALI0B20504g	0.78	0.72	similar to uniprot|P07280 Saccharomyces cerevisiae YOL121c RP55A 40S small subunit ribosomal protein S19 or uniprot|P07281 Saccharomyces cerevisiae YNL302c RP55B 40S small subunit ribosomal protein S19
YALI0A00803g	0.77	0.30	similar to uniprot|P39730 Saccharomyces cerevisiae YAL035w FUN12 general translation factor eIF2 homolog
YALI0B15103g	0.75	0.83	highly similar to uniprot|P0C0W9 Saccharomyces cerevisiae YPR102c RPL16A and highly similar to uniprot|Q3E757 Saccharomyces cerevisiae YGR085C RPL16B Ribosomal Protein of the Large subunit
YALI0C03674g	0.74	0.91	similar to uniprot|P13188 Saccharomyces cerevisiae YOR168W Glutaminyl-tRNA synthetase (EC 6.1.1.18) (Glutamine--tRNA ligase) (GlnRS)
YALI0A00264g	0.72	0.91	similar to uniprot|P09436 Saccharomyces cerevisiae YBL076c ILS1 isoleucyl-tRNA synthetase
YALI0B04334g	0.69	0.73	similar to uniprot|Q08972 Saccharomyces cerevisiae YPL226w NEW1 translation elongation factor eEF3-like
YALI0D07326g	0.69	0.81	highly similar to uniprot|P25039 Saccharomyces cerevisiae YLR069c MEF1 translation elongation factor G mitochondrial
YALI0E05005g	0.69	0.96	similar to uniprot|P38707 Saccharomyces cerevisiae YHR019c DED81 asparaginyl-tRNA-synthetase
YALI0B21076g	0.67	0.63	highly similar to uniprot|P02406 Saccharomyces cerevisiae YGL103w CYH2 60S large subunit ribosomal protein L27a
YALI0F29843g	0.67	0.74	similar to uniprot|P00958 Saccharomyces cerevisiae YGR264c MES1 methionyl-tRNA synthetase
YALI0A09922g	0.67	0.67	similar to uniprot|P39741 Saccharomyces cerevisiae YDL136w SOS2 60S large subunit ribosomal protein
YALI0B08748g	0.67	0.50	highly similar to uniprot|P41057 Saccharomyces cerevisiae YLR388w YS29A ribosomal protein S29
YALI0E24475g	0.67	0.72	highly similar to uniprot|P04649 Saccharomyces cerevisiae YLR406c 60S large subunit ribosomal protein L31B (L34B) (YL28)
YALI0E31955g	0.67	0.93	highly similar to uniprot|P41056 Saccharomyces cerevisiae YOR234c RPL37B ribosomal protein L35a.e.c15 P2. 57.f2.1
YALI0F05803g	0.67	0.60	highly similar to uniprot|Q01855 Saccharomyces cerevisiae YOL040c RPS21 40S small subunit ribosomal protein
YALI0D22264g	0.64	0.86	highly similar to uniprot|P04802 Saccharomyces cerevisiae YLL018c DPS1 aspartyl-tRNA synthetase cytosolic P6.12.f3.1
YALI0D13882g	0.63	0.63	highly similar to uniprot|P17079 Saccharomyces

			cerevisiae YEL054c RPL12A and highly similar to uniprot|P17079 Saccharomyces cerevisiae YDR418W RPL12B Ribosomal Protein of the Large subunit
YALI0B08250g	0.61	0.79	highly similar to uniprot|Q9HGI5 Yarrowia lipolytica eukaryotic peptide chain release factor GTP- binding subunit 3
YALI0C06886g	0.61	0.50	highly similar to uniprot|P19211 Saccharomyces cerevisiae YJR047c ANB1 translation initiation factor eIF5A.2 P2.324.f2.1
YALI0F00880g	0.61	0.36	similar to uniprot|P25491 Saccharomyces cerevisiae YNL064c YDJ1 mitochondrial and ER import protein
YALI0E22979g	0.60	1.05	similar to uniprot|O13432 Candida albicans FRS1 Phenylalanyl-tRNA synthetase beta chain (EC 6.1.1.20)
YALI0B14146g	0.60	0.73	highly similar to uniprot|P05317 Saccharomyces cerevisiae YLR340w RPLA0 acidic ribosomal protein L10.e singleton
YALI0A03839g	0.57	0.72	highly similar to uniprot|P32481 Saccharomyces cerevisiae YER025w GCD11 translation initiation factor eIF2 gamma chain
YALI0B20482g	0.55	0.42	highly similar to uniprot|P50085 Saccharomyces cerevisiae YGR231c PHB2 Prohibitin P2.384.f2.1
YALI0F05544g	0.54	0.67	highly similar to wi|NCU01949.1 Neurospora crassa NCU01949.1 and uniprot|P05755 Saccharomyces cerevisiae YBR189w SUP46 ribosomal protein
YALI0E14058g	0.52	0.31	similar to uniprot|P38922 Saccharomyces cerevisiae YNL004w HRB1
YALI0D22572g	0.51	0.67	similar to uniprot|O14164 Schizosaccharomyces pombe Probable eukaryotic translation initiation factor 3 93 kDa subunit (eIF3 p93)
YALI0C21472g	0.51	0.68	similar to uniprot|P06634 Saccharomyces cerevisiae YOR204w DED1 ATP-dependent RNA helicase P28.1.f22.1
YALI0F16115g	0.51	0.66	similar to uniprot|P06103 Saccharomyces cerevisiae YOR361c PRT1 translation initiation factor eIF3 subunit
YALI0D09449g	0.50	0.61	highly similar to uniprot|P20447 Saccharomyces cerevisiae YGL078C Probable ATP-dependent RNA helicase DBP3 (Helicase CA3)
YALI0F02629g	0.49	0.33	highly similar to uniprot|P07284 Saccharomyces cerevisiae YDR023w SES1 seryl-tRNA synthetase cytosolic
YALI0C11473g	0.48	0.52	highly similar to uniprot|Q08745 Saccharomyces cerevisiae YOR293W RPS10A and highly similar to uniprot|P46784 Saccharomyces cerevisiae YMR230W RPS10B Ribosomal Protein of the Small subunit
YALI0B15840g	0.48	0.50	similar to uniprot|Q12449 Saccharomyces cerevisiae YDR214W Hypothetical 39.4 kDa protein
YALI0A00594g	0.46	0.24	similar to uniprot|P32527 Saccharomyces cerevisiae YGR285c ZUO1 zuotin a putative Z-DNA binding protein
YALI0D10263g	0.46	0.63	highly similar to uniprot|P04451 Saccharomyces cerevisiae YER117w RPL23B ribosomal protein L23.e

YALI0E01782g	0.46	0.99	similar to uniprot|Q03532 Saccharomyces cerevisiae YMR290c HAS1 helicase associated with SET1P
YALI0E17985g	0.46	0.68	similar to uniprot|Q05506 Saccharomyces cerevisiae YDR341c arginyl-tRNA synthetase cytosolic
YALI0F20416g	0.45	0.23	highly similar to uniprot|P39078 Saccharomyces cerevisiae YDL143w CCT4 component of chaperonin-containing T-complex
YALI0D14916g	0.45	0.70	similar to uniprot|P09880 Saccharomyces cerevisiae YJL087c TRL1 tRNA ligase
YALI0B05610g	0.44	0.28	similar to uniprot|P38910 Saccharomyces cerevisiae YOR020C 10 kDa heat shock protein mitochondrial (HSP10) (10 kDa chaperonin
YALI0E26851g	0.43	0.23	similar to uniprot|Q9P748 Neurospora crassa Related to translation initiation factor 3
YALI0B00990g	0.43	0.49	highly similar to uniprot|Q02892 Saccharomyces cerevisiae YPL093W Nucleolar GTP-binding protein 1
YALI0E06765g	0.42	0.69	similar to uniprot|P38205 Saccharomyces cerevisiae YBL024w NCL1 tRNA (cytosine-5-)-methyltransferase
YALI0F05808g	0.40	0.33	highly similar to uniprot|P05319 Saccharomyces cerevisiae YOL039w RPLA2 acidic ribosomal protein P2.beta
YALI0B15774g	0.40	0.51	highly similar to uniprot|P12612 Saccharomyces cerevisiae YDR212w CCT1 component of chaperonin-containing T-complex P10.3.f8.1
YALI0E04719g	0.38	0.31	similar to uniprot|P40961 Saccharomyces cerevisiae YGR132c PHB1 prohibitin antiproliferative protein
YALI0C00605g	0.38	0.19	similar to uniprot|P38879 Saccharomyces cerevisiae YHR193c EGD2 alpha subunit of the nascent polypeptide-associated complex
YALI0E30217g	0.38	0.45	similar to uniprot|Q05022 Saccharomyces cerevisiae YMR229c RRP5 processing of pre-ribosomal RNA P2.264.f2.1
YALI0D13794g	0.33	0.44	highly similar to uniprot|P05760 Saccharomyces cerevisiae YJL136c RPS25B 40S small subunit ribosomal protein
YALI0F25399g	0.33	0.39	highly similar to uniprot|Q3E7Y3 Saccharomyces cerevisiae YLR367w RPS22B and highly similar to uniprot|P0C0W1 Saccharomyces cerevisiae YJL190c RPS22A Ribosomal Protein of the Small subunit
YALI0D16643g	0.30	0.14	similar to uniprot|P15625 Saccharomyces cerevisiae YFL022c FRS2 phenylalanine--tRNA ligase beta chain cytosolic
YALI0B01034g	0.29	0.23	highly similar to uniprot|P56286 Schizosaccharomyces pombe Eukaryotic translation initiation factor 2 alpha subunit (eIF-2- alpha)
YALI0C20999g	0.29	0.10	highly similar to uniprot|P42943 Saccharomyces cerevisiae YJL111w CCT7 component of chaperonin-containing T-complex P10.3.f8.1
YALI0B23342g	0.29	0.35	highly similar to uniprot|P53261 Saccharomyces cerevisiae YGR103w similarity to zebrafish essential for

			embryonic development gene pescadillo singleton
YALI0E03432g	0.27	0.35	similar to uniprot|P46672 Saccharomyces cerevisiae YGL105w G4P1 protein with specific affinity for G4 quadruplex nucleic acids
YALI0B14751g	0.26	0.35	highly similar to uniprot|P53914 Saccharomyces cerevisiae YNL132W Hypothetical 119.3 kDa protein in FPR1- TOM22 intergenic region
YALI0B10560g	0.25	0.53	similar to uniprot|P40991 Saccharomyces cerevisiae YNL061w NOP2 nucleolar protein
YALI0D16291g	0.24	0.11	similar to uniprot|P53893 Saccharomyces cerevisiae YNL163c translation elongation factor eEF4
YALI0B16896g	0.24	0.32	similar to uniprot|P32892 Saccharomyces cerevisiae YLL008w DRS1 RNA helicase of the DEAD box family P28.1.f22. 1
YALI0D12210g	0.24	0.28	similar to uniprot|P47047 Saccharomyces cerevisiae YJL050W ATP-dependent RNA helicase DOB1
YALI0D15268g	0.22	0.28	similar to uniprot|P53852 Saccharomyces cerevisiae YNL247w cysteinyl-tRNA synthetase
YALI0D12012g	0.21	0.19	similar to uniprot|P39744 Saccharomyces cerevisiae YOR206w (RAD4) crucial for intranuclear movement of ribosomal precursor
YALI0C16049g	0.21	0.08	some similarities with uniprot|P26642 Xenopus laevis Elongation factor 1-gamma type 1 (EF-1-gamma) (P47)
YALI0D24299g	0.19	0.22	similar to uniprot|P78954 Schizosaccharomyces pombe Eukaryotic translation initiation factor 4E-1 CDC33 homologue
YALI0D17732g	0.18	0.27	similar to uniprot|P33892 Saccharomyces cerevisiae YGL195w GCN1 translational activator
YALI0F17358g	0.18	0.29	similar to uniprot|Q04373 Saccharomyces cerevisiae YDR496c
YALI0F22671g	0.18	0.17	similar to uniprot|O94513 Schizosaccharomyces pombe Eukaryotic translation initiation factor 3 subunit 6 (eIF- 3 p48)
YALI0E29506g	0.18	0.21	similar to uniprot|P42945 Saccharomyces cerevisiae YJL109c weak similarity to ATPase DRS2P
YALI0D11242g	0.17	0.22	similar to uniprot|P47077 Saccharomyces cerevisiae YJL010C Hypothetical 77.7 kDa protein in CCT3-CCT8 intergenic region
YALI0C21956g	0.14	0.21	similar to uniprot|P53145 Saccharomyces cerevisiae YGL099w
YALI0B15708g	0.14	0.18	similar to uniprot|P32501 Saccharomyces cerevisiae YDR211w GCD6 translation initiation factor eIF2b epsilon 81 kDa subunit
YALI0C21846g	0.12	0.13	highly similar to uniprot|Q08965 Saccharomyces cerevisiae YPL217c BMS1 Ribosome biogenesis protein
YALI0F31471g	0.10	0.17	similar to DEHA0F17490g Debaryomyces hansenii IPF 7716.1 and uniprot|P40362 Saccharomyces cerevisiae YJL069c
YALI0A21197g	0.04	0.05	similar to uniprot|P48234 Saccharomyces cerevisiae YGR145w

S1B TABLE. Amino acid and protein metabolism class

Gene ID	Mean PAI per control replicate	Mean PAI per EgDGAT1-1 replicate	Protein description in Génolevures database
YALI0E09603g	2.76	2.68	similar to uniprot|P33327 Saccharomyces cerevisiae YDL215c GDH2 NAD-specific glutamate dehydrogenase (NAD)
YALI0E12683g	2.32	3.05	highly similar to uniprot|P05694 Saccharomyces cerevisiae YER091c MET6 5- methyltetrahydropteroyltriglutamate--homocysteine methyltransferase
YALI0B01364g	2.04	3.51	uniprot|Q9P3Y5 Yarrowia lipolytica 3- isopropylmalate dehydratase
YALI0C10230g	1.95	2.38	highly similar to uniprot|P14832 Saccharomyces cerevisiae YDR155C Peptidyl-prolyl cis-trans isomerase (EC 5.2.1.8) (PPIase) (Rotamase)(Cyclophilin) (Cyclosporin A- binding protein) (CPH)(PPI-II)
YALI0B02178g	1.76	2.76	similar to uniprot|P23542 Saccharomyces cerevisiae YLR027c AAT2 aspartate aminotransferase
YALI0E03036g	1.73	1.36	similar to uniprot|P55059 Humicola insolens Protein disulfide isomerase precursor
YALI0A14806g	1.70	2.26	similar to uniprot|P11913 Neurospora crassa Mitochondrial processing peptidase beta subunit
YALI0E27808g	1.63	2.04	similar to uniprot|P38911 Saccharomyces cerevisiae YML074c NPI46 proline cis-trans isomerase
YALI0E06017g	1.37	1.51	similar to uniprot|P22515 Saccharomyces cerevisiae YKL210w UBA1 E1-like (ubiquitin-activating) enzyme
YALI0C04433g	1.24	2.00	highly similar to uniprot|Q92413 Emericella nidulans Ornithine aminotransferase (EC 2.6.1.13) (Ornithine--oxo-acid aminotransferase)
YALI0C23969g	1.20	1.34	highly similar to uniprot|P03965 Saccharomyces cerevisiae YJR109c CPA2 arginine-specific carbamoylphosphate synthase large chain
YALI0B14509g	1.19	1.62	highly similar to uniprot|Q9P842 Candida albicans S-adenosylmethionine synthetase 2 (CaSAM2)
YALI0E07271g	1.18	0.76	similar to uniprot|P32528 Saccharomyces cerevisiae YBR208c DUR1_2 urea amidolyase
YALI0C00253g	1.13	1.51	highly similar to uniprot|P07342 Saccharomyces cerevisiae YMR108W Acetolactate synthase mitochondrial precursor (EC 4.1.3.18) (Acetohydroxy-acid synthase) (ALS) (AHAS)
YALI0A13387g	1.10	1.20	highly similar to uniprot|P49090 Saccharomyces cerevisiae YGR124w ASN2 asparagine synthetase
YALI0E06457g	1.06	1.31	similar to uniprot|P07702 Saccharomyces cerevisiae YBR115c LYS2 L-aminoadipate-semialdehyde dehydrogenase large subunit
YALI0B09647g	1.06	1.28	similar to uniprot|P07275 Saccharomyces cerevisiae YHR037W Delta-1-pyrroline-5-carboxylate dehydrogenase mitochondrial precursor (EC 1.5.1.12) (P5C

			dehydrogenase)
YALI0F12639g	0.93	0.94	similar to uniprot|P08566 Saccharomyces cerevisiae YDR127W Pentafunctional AROM polypeptide
YALI0F02849g	0.92	0.90	similar to uniprot|P48015 Saccharomyces cerevisiae YDR019c GCV1 glycine decarboxylase
YALI0D03135g	0.83	1.13	highly similar to uniprot|P06168 Saccharomyces cerevisiae YLR355c ILV5 ketol-acid reducto-isomerase
YALI0B19580g	0.79	0.97	similar to uniprot|Q08225 Saccharomyces cerevisiae YOL057W Probable dipeptidyl-peptidase III (EC 3.4.14.4)
YALI0F29337g	0.76	0.86	similar to uniprot|P23542 Saccharomyces cerevisiae YLR027c AAT2 aspartate aminotransferase cytosolic and wi|NCU07941.1 Neurospora crassa
YALI0F09834g	0.74	0.74	similar to uniprot|P32565 Saccharomyces cerevisiae YIL075c SEN3 26S proteasome regulatory subunit and DEHA0B11220g Debaryomyces hansenii
YALI0B07447g	0.73	1.25	similar to uniprot|P06208 Saccharomyces cerevisiae YNL104C 2-isopropylmalate synthase (EC 4.1.3.12) (Alpha- isopropylmalate synthase) (Alpha-IPM synthetase)
YALI0D06930g	0.71	0.82	similar to uniprot|Q06408 Saccharomyces cerevisiae YDR380W ARO10 phenylpyruvate decarboxylase, catalyzes decarboxylation of phenylpyruvate to phenylacetaldehyde
YALI0A09856g	0.70	1.06	similar to uniprot|P49095 Saccharomyces cerevisiae YMR189w GSD2 glycine decarboxylase P subunit
YALI0E07535g	0.69	0.87	similar to uniprot|P00812 Saccharomyces cerevisiae YPL111w CAR1 arginase singleton
YALI0E09108g	0.67	0.32	similar to uniprot|P32582 Saccharomyces cerevisiae YGR155w CYS4 cystathionine beta-synthase
YALI0F20988g	0.66	0.84	similar to uniprot|P25375 Saccharomyces cerevisiae YCL057W Saccharolysin (EC 3.4.24.37) (Protease D) (Proteinase yscD) (Oligopeptidase YSCD)
YALI0F17974g	0.64	0.58	similar to uniprot|Q01532 Saccharomyces cerevisiae YNL239w Cysteine proteinase 1 (EC 3.4.22.40) (Y3) (Bleomycin hydrolase) (BLM hydrolase)
YALI0C03355g	0.62	0.55	highly similar to uniprot|P38219 Saccharomyces cerevisiae YBR025c strong similarity to Ylf1p
YALI0D16335g	0.62	0.73	similar to uniprot|P50101 Saccharomyces cerevisiae YMR304w UBP15
YALI0C09636g	0.60	0.64	similar to uniprot|P25605 Saccharomyces cerevisiae YCL009C Acetolactate synthase small subunit(mitochondrial precursor)
YALI0D22891g	0.60	0.77	highly similar to uniprot|P38999 Saccharomyces cerevisiae YNR050C Saccharopine dehydrogenase [NADP L-glutamate forming] (EC 1.5.1.10) (Saccharopine reductase)
YALI0C12661g	0.59	0.56	uniprot|Q873M5 Yarrowia lipolytica UDP-Glc:glycoprotein glucosyltransferase
YALI0B14641g	0.58	0.78	similar to uniprot|P32454 Saccharomyces cerevisiae YKL157w APE2 aminopeptidase yscII
YALI0B11594g	0.57	0.84	similar to uniprot|Q96UQ4 Aspergillus niger Aminopeptidase B

YALI0E16346g	0.57	0.64	highly similar to uniprot|O13426 Candida albicans Serine hydroxymethyltransferase cytosolic (EC 2.1.2.1) (Serine methylase) (Glycine hydroxymethyltransferase) (SHMT) (SHMII)
YALI0F24893g	0.57	0.74	similar to uniprot|P00931 Saccharomyces cerevisiae YGL026c TRP5 tryptophan synthase
YALI0B16500g	0.55	0.40	similar to uniprot|P09232 Saccharomyces cerevisiae YEL060c PRB1 protease B vacuolar P3.40.f3.1
YALI0A15950g	0.55	0.75	similar to uniprot|P00815 Saccharomyces cerevisiae YCL030c HIS4 phosphoribosyl-AMP cyclohydrolase/phosphoribosyl-ATP pyrophosphatase/histidinol dehydrogenase
YALI0B16104g	0.50	0.47	highly similar to uniprot|P38625 Saccharomyces cerevisiae YMR217w GUA1 GMP synthase (glutamine- hydrolyzing) singleton
YALI0B15444g	0.50	0.70	uniprot|P38997 Yarrowia lipolytica Saccharopine dehydrogenase
YALI0C01859g	0.49	0.43	similar to uniprot|Q02253 Rattus norvegicus Methylmalonate-semialdehyde dehydrogenase (acylating) mitochondrial precursor (EC 1.2.1.27) (MMSDH)
YALI0D01265g	0.49	0.46	similar to uniprot|P38891 Saccharomyces cerevisiae YHR208w BAT1 branched chain amino acid aminotransferase mitochondrial precursor
YALI0A10615g	0.48	0.49	similar to uniprot|P39925 Saccharomyces cerevisiae YER017c AFG3 protease of the SEC18/CDC48/PAS1 family of ATPases (AAA)
YALI0F17820g	0.46	0.32	highly similar to uniprot|P39708 Saccharomyces cerevisiae YAL062w GDH3 NADP-glutamate dehydrogenase or uniprot|P07262 Saccharomyces cerevisiae YOR375c GDH1 glutamate dehydrogenase (NADP+)
YALI0E20977g	0.46	0.19	similar to uniprot|P53090 Saccharomyces cerevisiae YGL202w ARO8 aromatic amino acid aminotransferase I
YALI0E02728g	0.42	0.51	highly similar to DEHA0F08019g Debaryomyces hansenii and uniprot|P49367 Saccharomyces cerevisiae YDR234w LYS4 homoaconitase
YALI0C16775g	0.42	0.33	similar to uniprot|P53691 Saccharomyces cerevisiae YLR216c CPR6 member of the cyclophilin family P8.4.f6.1
YALI0F19910g	0.41	0.24	similar to uniprot|P47176 Saccharomyces cerevisiae YJR148w TWT2 branched chain amino acid aminotransferase cytosolic
YALI0B02860g	0.40	0.52	similar to uniprot|P38764 Saccharomyces cerevisiae YHR027C 26S proteasome regulatory subunit RPN1 (Proteasome non-ATPase subunit 1)
YALI0C09988g	0.39	0.39	similar to uniprot|P18253 Schizosaccharomyces pombe Peptidyl-prolyl cis-trans isomerase
YALI0F09966g	0.38	0.24	similar to uniprot|P40054 Saccharomyces cerevisiae YER081w SER3 3-phosphoglycerate dehydrogenase and DEHA0B11198g Debaryomyces hansenii
YALI0B20020g	0.37	0.69	highly similar to uniprot|P14843 Saccharomyces cerevisiae

			YDR035w ARO3 2-dehydro-3-deoxyphosphoheptonate aldolase phenylalanine-inhibited P2.438.f2.1
YALI0B19998g	0.37	0.46	similar to uniprot|Q12680 Saccharomyces cerevisiae YDL171c GLT1 glutamate synthase (NAPDPH) (GOGAT
YALI0F17842g	0.33	0.28	highly similar to uniprot|P43616 Saccharomyces cerevisiae YFR044c Glutamate carboxypeptidase-like protein
YALI0E13508g	0.33	0.33	similar to uniprot|P25294 Saccharomyces cerevisiae YNL007c SIS1 heat shock protein
YALI0E20119g	0.33	0.17	similar to uniprot|P40029 Saccharomyces cerevisiae YER042w MXR1 responsible for the reduction of methionine sulfoxide
YALI0D12452g	0.32	0.24	similar to uniprot|P54860 Saccharomyces cerevisiae YDL190c UFD2 ubiquitin fusion degradation protein
YALI0F07667g	0.31	0.56	similar to uniprot|P25375 Saccharomyces cerevisiae YCL057w PRD1 proteinase yscD
YALI0C06952g	0.31	0.40	highly similar to uniprot|P32449 Saccharomyces cerevisiae YBR249C Phospho-2-dehydro-3-deoxyheptonate aldolase tyrosine-inhibited (EC 4.1.2.15) (Phospho-2-keto- 3-deoxyheptonate aldolase) (DAHP synthetase) (3-deoxy-D-arabino-heptulosonate 7-phosphate synthase
YALI0D07370g	0.31	0.26	similar to uniprot|P87183 Trichoderma virens Carbamoyl-phosphate synthase arginine-specific small chain mitochondrial precursor
YALI0F23221g	0.31	0.31	similar to uniprot|P16120 Saccharomyces cerevisiae Threonine synthase (EC 4.2.3.1) (TS) YCR053w (o-p-homoserine p-lyase)
YALI0D11132g	0.30	0.25	similar to uniprot|P36037 Saccharomyces cerevisiae YKL213c DOA1 involved in ubiquitin-dependent proteolysis P29.1.f3.1
YALI0B17666g	0.29	0.32	similar to uniprot|Q01217 Saccharomyces cerevisiae YER069w ARG5_6 acetylglutamate kinase and acetylglutamyl- phosphate reductase
YALI0E18238g	0.27	0.50	similar to uniprot|P17649 Saccharomyces cerevisiae YGR019w UGA1 4-aminobutyrate aminotransferase (GABA transaminase)
YALI0F00506g	0.27	0.23	highly similar to uniprot|P32288 Saccharomyces cerevisiae YPR035w GLN1 glutamate--ammonia ligase and DEHA0G20317g Debaryomyces hansenii IPF 5106.1
YALI0E12595g	0.26	0.09	similar to uniprot|Q9V9A7 Drosophila melanogaster Putative propionyl-CoA carboxylase beta chain mitochondrial precursor (EC 6.4.1.3) (PCCase beta subunit) (Propanoyl-CoA:carbon dioxide ligase beta subunit)
YALI0D06325g	0.25	0.22	similar to uniprot|P52893 Saccharomyces cerevisiae YLR089C Putative alanine aminotransferase mitochondrial precursor
YALI0F27071g	0.25	0.19	similar to uniprot|O14413 Pichia angusta proteinase A and uniprot|P07267 Saccharomyces cerevisiae YPL154c PEP4 aspartyl protease
YALI0A21417g	0.25	0.21	similar to uniprot|O74267 Ashbya gossypii Threonine aldolase

YALI0F02585g	0.25	0.17	similar to uniprot|P40327 Saccharomyces cerevisiae YDL007w YTA5 26S proteasome regulatory subunit
YALI0B14465g	0.24	0.38	similar to uniprot|P46151 Saccharomyces cerevisiae YPL023c MET12 methylenetetrahydrofolate reductase P2.74.f2. 1
YALI0D09119g	0.23	0.12	highly similar to uniprot|P33297 Saccharomyces cerevisiae YOR117w YTA1 26S proteasome regulatory subunit
YALI0D23309g	0.21	0.51	similar to uniprot|P37898 Saccharomyces cerevisiae YHR047c AAP1 alanine/arginine aminopeptidase
YALI0D11770g	0.21	0.11	highly similar to uniprot|P33298 Saccharomyces cerevisiae YDR394w YTA2 26S proteasome regulatory subunit
YALI0D17204g	0.20	0.13	highly similar to uniprot|Q01939 Saccharomyces cerevisiae YGL048c SUG1 26S proteasome regulatory subunit
YALI0B02574g	0.19	0.29	similar to uniprot|P39925 Saccharomyces cerevisiae YER017C Mitochondrial respiratory chain complexes assembly protein AFG3 (EC 3.4.24.-) (TAT-binding homolog 10)
YALI0F12221g	0.19	0.21	similar to uniprot|P12945 Saccharomyces cerevisiae YDL040c
YALI0C23408g	0.18	0.27	highly similar to uniprot|P39522 Saccharomyces cerevisiae YJR016c ILV3 Dihydroxy-acid dehydratase mitochondrial precursor
YALI0F11033g	0.18	0.18	similar to uniprot|P47154 Saccharomyces cerevisiae YJR117w STE24 zinc metallo-protease and DEHA0F06820g Debaryomyces hansenii
YALI0D09977g	0.17	0.10	similar to uniprot|Q12250 Saccharomyces cerevisiae YDL147W 26S proteasome regulatory subunit RPN5 (Proteasome non-ATPase subunit 5)
YALI0E02398g	0.17	0.16	similar to uniprot|CAD70763 Neurospora crassa 80A10. 060 Related to 5-oxoprolinase
YALI0D22484g	0.17	0.46	similar to uniprot|P37292 Saccharomyces cerevisiae YBR263w SHM1 serine hydroxymethyltransferase precursor mitochondrial
YALI0D05665g	0.15	0.13	similar to uniprot|P40157 Saccharomyces cerevisiae YNL212w VID27
YALI0B14399g	0.13	0.26	similar to uniprot|Q12740 Zalerion arboricola Pyrroline- 5-carboxylate reductase PRO3 (P5C reductase)
YALI0F29381g	0.13	0.29	some similarities with uniprot|O93914 Aspergillus niger PDI related protein A
YALI0B01386g	0.12	0.23	similar to uniprot|P32795 Saccharomyces cerevisiae YPR024W YME1 protease of the SEC18/CDC48/PAS1 family of ATPases (AAA)
YALI0D08976g	0.12	0.06	similar to uniprot|Q12377 Saccharomyces cerevisiae YDL097c RPN6 subunit of the regulatory particle of the proteasome
YALI0D26367g	0.10	0.12	highly similar to uniprot|P04076 Saccharomyces cerevisiae YHR018c ARG4 arginosuccinate lyase
YALI0C19382g	0.10	0.17	highly similar to uniprot|P23638 Saccharomyces cerevisiae YGR135w PRE9 20S proteasome subunit Y13 (alpha3) P7.1.f7.1

YALI0D20966g	0.10	0.14	similar to uniprot|P38615 Saccharomyces cerevisiae YMR139w MDS1 ser/thr protein kinase
YALI0E06501g	0.08	0.10	similar to uniprot|P12688 Saccharomyces cerevisiae YKL126w YPK1 ser/thr-specific protein kinase
YALI0B22902g	0.07	0.07	similar to uniprot|Q05979 Saccharomyces cerevisiae YLR231c Probable kynureninase (EC 3.7.1.3) (L-kynurenine hydrolase
YALI0D05159g	0.06	0.57	similar to uniprot|P38174 Saccharomyces cerevisiae YBL091c MAP2 methionine aminopeptidase isoform 2

S1C TABLE. Energetic metabolism class

Gene ID	Mean PAI per control replicate	Mean PAI per EgDGAT1-1 replicate	Protein description in Génolevures database
YALI0F16819g	5.29	6.29	highly similar to uniprot|P00925 Saccharomyces cerevisiae YHR174w ENO2 enolase or uniprot|P00924 Saccharomyces cerevisiae YGR254w ENO1 enolase
YALI0D09361g	4.31	4.52	highly similar to uniprot|P19414 Saccharomyces cerevisiae YLR304C Aconitate hydratase mitochondrial precursor (EC 4.2.1.3) (Citrate hydro-lyase) (Aconitase)
YALI0D16753g	3.74	4.86	highly similar to uniprot|Q8TG27 Talaromyces emersonii Malate dehydrogenase precursor mitochondrial
YALI0B03982g	2.75	3.48	highly similar to uniprot|P00830 Saccharomyces cerevisiae YJR121W ATP synthase beta chain mitochondrial precursor (EC 3.6.3.14)
YALI0E33517g	2.59	3.15	highly similar to uniprot|P20967 Saccharomyces cerevisiae YIL125w KGD1 2-oxoglutarate dehydrogenase complex E1 component singleton
YALI0C06776g	2.33	2.50	highly similar to uniprot|P55250 Rhizopus oryzae Fumarate hydratase mitochondrial precursor (EC 4.2.1.2) (Fumarase)
YALI0C16995g	2.30	3.89	highly similar to uniprot|P10963 Saccharomyces cerevisiae YKR097w PCK1 phosphoenolpyruvate carboxykinase
YALI0C24101g	2.07	3.01	highly similar to uniprot|P11154 Saccharomyces cerevisiae YGL062w PYC1 pyruvate carboxylase 1
YALI0F03179g	1.97	1.88	highly similar to uniprot|P07251 Saccharomyces cerevisiae YBL099w ATP1 F1F0-ATPase complex F1 alpha subunit
YALI0B13970g	1.96	2.85	similar to uniprot|P32191 Saccharomyces cerevisiae YIL155c GUT2 glycerol-3-phosphate dehydrogenase mitochondrial
YALI0E26004g	1.93	3.02	similar to uniprot|P14540 Saccharomyces cerevisiae YKL060c FBA1 fructose-bisphosphate aldolase
YALI0F09185g	1.92	1.99	uniprot|P30614 Yarrowia lipolytica Pyruvate kinase
YALI0B15598g	1.81	2.08	highly similar to uniprot|P38720 Saccharomyces cerevisiae YHR183w GND1 6-phosphogluconate dehydrogenase P2.360.f2.1 or uniprot|P53319 Saccharomyces cerevisiae YGR256w GND2 phosphogluconate dehydrogenase P2.360.f2.1
YALI0D04741g	1.76	2.30	highly similar to uniprot|Q9P567 Neurospora crassa Probable succinyl-CoA ligase (GDP-forming) beta-chain mitochondrial precursor
YALI0C16885g	1.75	1.70	uniprot|P41555 Yarrowia lipolytica Isocitrate lyase
YALI0E27005g	1.75	2.04	highly similar to uniprot|P32473 Saccharomyces cerevisiae YBR221c PDB1 pyruvate dehydrogenase (lipoamide) beta chain precursor
YALI0D23683g	1.65	1.73	similar to uniprot|P12695 Saccharomyces cerevisiae YNL071W Dihydrolipoamide acetyltransferase component of pyruvate dehydrogenase complex mitochondrial

			precursor (PDC-E2)
YALI0D06303g	1.60	2.51	highly similar to uniprot|P28241 Saccharomyces cerevisiae YOR136w IDH2 isocitrate dehydrogenase
YALI0E05137g	1.58	3.00	similar to uniprot|P28834 Saccharomyces cerevisiae YNL037c IDH1 isocitrate dehydrogenase (NAD+) subunit 1 mitochondrial
YALI0F15587g	1.54	2.02	highly similar to uniprot|P15019 Saccharomyces cerevisiae YLR354c TAL1 transaldolase
YALI0D05467g	1.52	2.01	uniprot|Q9UUU3 Yarrowia lipolytica NUAM protein precursor
YALI0E14190g	1.51	1.82	similar to uniprot|P17505 Saccharomyces cerevisiae YKL085w MDH1 malate dehydrogenase precursor mitochondrial
YALI0F20702g	1.42	2.00	highly similar to uniprot|P16387 Saccharomyces cerevisiae YER178w PDA1 pyruvate dehydrogenase (lipoamide) alpha chain precursor
YALI0D11374g	1.23	1.83	similar to uniprot|P47052 Saccharomyces cerevisiae Probable succinate dehydrogenase [ubiquinone] flavoprotein subunit 2 mitochondrial precursor (EC 1.3.5.1) (FP) (Flavoprotein subunit of complex II) and uniprot|Q00711 Saccharomyces cerevisiae YKL148c SDH1 succinate dehydrogenase flavoprotein precursor P2.302.f2.1
YALI0B11913g	1.22	1.22	similar to uniprot|P81451 Saccharomyces cerevisiae YOL077w-a ATP19 Subunit K of mitochondrial ATP Synthase
YALI0D12400g	1.20	2.12	uniprot|P29407 Yarrowia lipolytica Phosphoglycerate kinase
YALI0F07711g	1.19	1.21	highly similar to uniprot|P12709 Saccharomyces cerevisiae YBR196c PGI1 glucose-6-phosphate isomerase
YALI0D12584g	1.13	1.03	similar to uniprot|Q9P602 Neurospora crassa ATP synthase oligomycin sensitivity conferring protein mitochondrial precursor
YALI0F05214g	1.13	1.08	similar to uniprot|P00942 Saccharomyces cerevisiae YDR050c TPI1 triose-phosphate isomerase singleton
YALI0F04095g	1.10	1.48	highly similar to uniprot|P41939 Saccharomyces cerevisiae YLR174w IDP2 isocitrate dehydrogenase
YALI0F25135g	1.09	1.63	uniprot|O74931 Yarrowia lipolytica Alternative NADH-dehydrogenase precursor
YALI0D16357g	1.09	1.59	uniprot|P59680 Yarrowia lipolytica 6- phosphofructokinase
YALI0B06831g	1.07	1.22	highly similar to uniprot|O13350 Kluyveromyces lactis ATP synthase D chain mitochondrial
YALI0D20768g	1.07	0.97	similar to uniprot|P09624 Saccharomyces cerevisiae YFL018c LPD1 dihydrolipoamide dehydrogenase precursor P3. 18.f3.1
YALI0D09273g	1.00	1.00	highly similar to uniprot|P00044 Saccharomyces cerevisiae YJR048w CYC1 cytochrome-c isoform 1
YALI0E24013g	0.97	0.82	similar to uniprot|P53598 Saccharomyces cerevisiae YOR142w LSC1 succinate-CoA ligase alpha subunit
YALI0F20306g	0.97	0.93	similar to uniprot|O13349 Kluyveromyces lactis ATP synthase subunit 4 mitochondrial precursor (EC 3.6.3.14)

			and uniprot|P05626 Saccharomyces cerevisiae YPL078c ATP4 F1F0- ATPase complex F0 subunit B singleton
YALI0F02893g	0.90	0.87	similar to uniprot|P38077 Saccharomyces cerevisiae YBR039w ATP3 F1F0-ATPase complex F1 gamma subunit
YALI0C06369g	0.89	0.96	highly similar to uniprot|Q9Y796 Cryptococcus curvatus Glyceraldehyde 3-phosphate dehydrogenase (EC 1.2. 1.12) (GAPDH)
YALI0D23397g	0.88	0.79	highly similar to uniprot|P21801 Saccharomyces cerevisiae YLL041C Succinate dehydrogenase [ubiquinone] iron-sulfur protein mitochondrial precursor (EC 1.3.5.1) (IP)
YALI0C20449g	0.87	0.64	similar to uniprot|P42940 Saccharomyces cerevisiae YGR207c ETF-BETA electron-transferring flavoprotein beta chain
YALI0B02728g	0.86	0.94	highly similar to uniprot|P00950 Saccharomyces cerevisiae YKL152C Phosphoglycerate mutase 1 (EC 5.4.2.1) (Phosphoglyceromutase 1) (PGAM 1) (MPGM 1) (BPG-dependent PGAM 1)
YALI0E34037g	0.83	0.94	similar to uniprot|P00128 Saccharomyces cerevisiae YDR529c QCR7 ubiquinol--cytochrome-c reductase subunit 7
YALI0D10593g	0.81	1.02	similar to uniprot|P40495 Saccharomyces cerevisiae YIL094c LYS12 homo-isocitrate dehydrogenase
YALI0D11814g	0.75	0.92	similar to uniprot|Q06405 Saccharomyces cerevisiae YDR377w ATP17 ATP synthase complex subunit f
YALI0A09900g	0.73	0.88	highly similar to uniprot|Q9UVJ8 Ashbya gossypii Vacuolar ATP synthase catalytic subunit A (V-ATPase 69 kDa subunit)
YALI0A14784g	0.67	0.67	some similarities with uniprot|P37298 Saccharomyces cerevisiae YDR178w SDH4 succinate dehydrogenase membrane anchor subunit for SDH2P
YALI0E16929g	0.67	0.52	similar to uniprot|P19262 Saccharomyces cerevisiae YDR148c KGD2 2-oxoglutarate dehydrogenase complex E2 component
YALI0F06061g	0.67	1.00	NADH-ubiquinone reductase complex accessory subunit 10 5 kDa
YALI0A17468g	0.67	0.86	similar to uniprot|P07143 Saccharomyces cerevisiae YOR065w CYT1 cytochrome-c1
YALI0B10340g	0.65	0.81	similar to uniprot|O42620 Magnaporthe grisea Carnitine acetyl transferase
YALI0F03567g	0.63	0.44	similar to uniprot|P00424 Saccharomyces cerevisiae YNL052w COX5A mitochondrial cytochrome-c oxidase chain V.A precursor
YALI0A02915g	0.63	0.58	similar to uniprot|P08067 Saccharomyces cerevisiae YEL024w RIP1 ubiquinol--cytochrome-c reductase iron-sulfur protein precursor singleton
YALI0A15972g	0.62	0.67	uniprot|Q7Z8Q0 Yarrowia lipolytica Fructose-1 6-bisphosphatase similar to uniprot|P09201 Saccharomyces cerevisiae YLR377c and similar to uniprot|P14065 YOR120W Saccharomyces cerevisiae

YALI0B02948g	0.60	0.42	uniprot|Q9UVF4 Yarrowia lipolytica Glycerol-3-phosphate dehydrogenase [NAD]
YALI0B00792g	0.57	0.62	uniprot|Q6CG53 Yarrowia lipolytica YALI0B00792g NADH- ubiquinone reductase accessory subunit 18.5 kDa (complex I)
YALI0A06765g	0.56	0.36	similar to uniprot|P40513 Saccharomyces cerevisiae YIL070c MAM33 mitochondrial acidic matrix protein
YALI0A02651g	0.56	0.67	similar to uniprot|P19968 Neurospora crassa NADH-ubiquinone oxidoreductase 21.3 kDa subunit (EC 1.6.5.3) (EC 1.6.99.3)
YALI0E13420g	0.55	0.48	similar to uniprot|Q9HZP7 Pseudomonas aeruginosa Electron transfer flavoprotein alpha-subunit
YALI0E15708g	0.54	0.56	similar to uniprot|P21826 Saccharomyces cerevisiae YIR031c DAL7 malate synthase 2
YALI0E02684g	0.52	0.56	highly similar to uniprot|P00890 Saccharomyces cerevisiae YNR001c CIT1 citrate (si)-synthase mitochondrial possible transmembrane segment
YALI0E14949g	0.51	0.80	highly similar to uniprot|P39533 Saccharomyces cerevisiae YJL200c strong similarity to aconitate hydratase
YALI0E22649g	0.51	0.44	similar to uniprot|P11412 Saccharomyces cerevisiae YNL241c ZWF1 glucose-6-phosphate dehydrogenase
YALI0E00638g	0.51	0.62	similar to uniprot|Q9TEM3 Emericella nidulans MCSA Methylcitrate synthase precursor
YALI0D09933g	0.48	0.76	similar to uniprot|Q8J0I8 Yarrowia lipolytica Alternative oxidase
YALI0F03201g	0.44	0.33	similar to uniprot|P32799 Saccharomyces cerevisiae YGL191w COX13 cytochrome-c oxidase chain VIa singleton
YALI0D24585g	0.44	0.61	similar to uniprot|Q86ZJ8 Podospora anserina NADH-ubiquinone reductase accessory subunit (complex I)
YALI0B20372g	0.42	0.53	uniprot|Q9UVT9 Yarrowia lipolytica NADH:ubiquinone oxidoreductase 51 kDa subunit nucleotide-binding subunit of NADH:ubiquinone oxidoreductase
YALI0D08602g	0.38	0.50	some similarities with uniprot|Q08023 Saccharomyces cerevisiae YLR077w
YALI0A01419g	0.38	0.57	uniprot|Q6CI60 Yarrowia lipolytica YALI0A01419g NADH- ubiquinone oxidoreductase 21.3 kDa accessory subunit
YALI0E30965g	0.37	0.16	similar to uniprot|P32316 Saccharomyces cerevisiae YBL015W ACH1 Acetyl-CoA hydrolase (EC 3.1.2.1) (Acetyl-CoA deacylase) (Acetyl-CoA acylase)
YALI0E02244g	0.35	0.54	similar to uniprot|Q03262 Saccharomyces cerevisiae YMR278w and DEHA0F03432g Debaryomyces hansenii
YALI0A20680g	0.33	0.29	similar to uniprot|P21976 Neurospora crassa NADH-ubiquinone oxidoreductase 20.8 kDa subunit
YALI0E31766g	0.33	0.60	similar to B18 Bos taurus accessory protein of NADH-ubiquinone reductase (complex I)
YALI0E28424g	0.28	0.28	similar to uniprot|Q02854 Neurospora crassa NADH-ubiquinone oxidoreductase 21 kDa subunit (Complex I)
YALI0E29095g	0.27	0.43	uniprot|Q6ZY23 Yarrowia lipolytica YALI0E29095g

			NUWM NADH-ubiquinone reductase accessory subunit (complexI)
YALI0F00924g	0.26	0.44	uniprot|Q9UUT8 Yarrowia lipolytica Subunit NUIM of protein NADH:ubiquinone oxidoreductase (Complex I) (EC 1.6. 99.3)
YALI0B14861g	0.17	0.28	similar to uniprot|P25711 Neurospora crassa NADH-ubiquinone oxidoreductase 21 kDa subunit mitochondrial precursor
YALI0E28930g	0.17	0.22	similar to B16.6 Bos taurus accessory protein of NADH-ubiquinone reductase (complex I)
YALI0C13508g	0.13	0.20	similar to uniprot|P14065 Saccharomyces cerevisiae YOR120w GCY1 galactose-induced protein of aldo/keto reductase family P6.5.f6.1 (EC 1.1.1.-)
YALI0E00264g	0.10	0.07	similar to uniprot|P46367 Saccharomyces cerevisiae YOR374w ALD4 aldehyde dehydrogenase mitochondrial possible transmembrane segment

S1D TABLE. Cellular transport and traffic class

Gene ID	Mean PAI per control replicate	Mean PAI per EgDGAT1-1 replicate	Protein description in Génolevures database
YALI0B22066g	7.80	8.89	highly similar to uniprot|P05030 Saccharomyces cerevisiae YGL008c PMA1 H+-transporting P-type ATPase major isoform plasma membrane
YALI0A10659g	3.11	3.71	uniprot|Q8J0M2 Yarrowia lipolytica ADP/ATP carrier protein
YALI0C20295g	2.87	3.20	some similarities with uniprot|P49573 Saccharomyces cerevisiae YPR124W Copper transport protein CTR1
YALI0D08228g	2.19	2.81	uniprot|Q9P8M1 Yarrowia lipolytica ADP/ATP carrier protein Aac1p
YALI0F14223g	1.86	2.00	similar to uniprot|P40035 Saccharomyces cerevisiae YER053c related to mitochondrial phosphate carrier protein
YALI0A17127g	1.75	1.74	similar to uniprot|P22137 Saccharomyces cerevisiae YGL206c CHC1 Clathrin heavy chain
YALI0F17314g	1.65	2.02	similar to uniprot|P04840 Saccharomyces cerevisiae YNL055c POR1 mitochondrial outer membrane porin
YALI0B13156g	1.41	1.20	uniprot|Q9HFC6 Yarrowia lipolytica Calnexin precursor involved in regulation of secretion
YALI0E30635g	1.04	0.88	uniprot|Q8J0E5 Yarrowia lipolytica Sec31p component of the COPII coat of ER-golgi vesicles
YALI0F04730g	1.02	0.95	uniprot|Q8TFK3 Yarrowia lipolytica GTP-binding protein
YALI0E19767g	0.98	0.88	similar to uniprot|P53622 Saccharomyces cerevisiae YDL145c RET1 coatomer complex alpha chain of secretory pathway vesicles
YALI0B10736g	0.83	0.57	highly similar to uniprot|Q04013 Saccharomyces cerevisiae YMR241w YHM2 yeast suppressor gene of HM (mitochondrial histone) mutant (ABF2) singleton
YALI0F19074g	0.78	0.79	similar to uniprot|P41810 Saccharomyces cerevisiae YDR238c SEC26 coatomer complex beta chain of secretory pathway vesicles
YALI0F10098g	0.75	0.74	similar to uniprot|P30822 Saccharomyces cerevisiae YGR218w CRM1 nuclear export factor exportin
YALI0D09713g	0.73	0.52	highly similar to uniprot|P21576 Saccharomyces cerevisiae YKR001c VPS1 member of the dynamin family of GTPases
YALI0B10362g	0.71	0.88	similar to uniprot|Q9HEG7 Neurospora crassa Mitochondrial precursor protein import receptor tom70
YALI0F05324g	0.69	0.64	similar to wi|NCU02391.1 Neurospora crassa NCU02391. 1 hypothetical protein and uniprot|P40482 Saccharomyces cerevisiae YIL109c SEC24 component of COPII coat of ER-Golgi vesicles
YALI0E27346g	0.68	0.49	highly similar to uniprot|P16140 Saccharomyces cerevisiae YBR127c VMA2 H+-ATPase V1 domain 60 KD subunit

			vacuolar
YALI0F03454g	0.68	0.83	similar to uniprot|P32074 Saccharomyces cerevisiae YNL287w SEC21 coatomer complex gamma chain (gamma-COP) of secretory pathway vesicles
YALI0B19668g	0.67	0.60	similar to uniprot|Q12402 Saccharomyces cerevisiae YPR028w YIP2 protein or Ypt Interacting Protein regulates vesicular traffic in stressed cells either to facilitate membrane turnover or to decrease unnecessary secretion
YALI0E16995g	0.65	0.67	similar to uniprot|P15303 Saccharomyces cerevisiae YPR181c SEC23 component of COPII coat of ER-golgi vesicles
YALI0D22803g	0.65	0.83	similar to uniprot|P40024 Saccharomyces cerevisiae Probable ATP-dependent transporter YER036C
YALI0E16324g	0.62	0.56	similar to uniprot|P46970 Saccharomyces cerevisiae YJR132w NMD5 NAM7P interacting protein
YALI0F02167g	0.58	0.54	highly similar to CA6093|CaARF22 Candida albicans CaARF22 or CA5095|CaARF21 Candida albicans CaARF21
YALI0D26147g	0.57	0.78	similar to uniprot|Q9C2K1 Neurospora crassa ARALAR1 Probable mitochondrial carrier protein
YALI0C21824g	0.56	0.46	similar to uniprot|P20606 Saccharomyces cerevisiae YPL218w SAR1 GTP-binding protein of the ARF family P7.3.f6. 1 no start
YALI0E12243g	0.55	0.59	similar to uniprot|P40069 Saccharomyces cerevisiae YER110c KAP123 RAN-binding protein
YALI0F21967g	0.55	0.42	similar to uniprot|P39986 Saccharomyces cerevisiae YEL031w SPF1 P-type ATPase
YALI0F28501g	0.46	0.21	similar to uniprot|Q02821 Saccharomyces cerevisiae YNL189w SRP1 karyopherin-alpha or importin
YALI0C14498g	0.44	0.53	highly similar to uniprot|P38988 Saccharomyces cerevisiae YDL198C Putative mitochondrial carrier protein YHM1/SHM1
YALI0D23947g	0.44	0.94	some similarities with uniprot|P25386 Saccharomyces cerevisiae YDL058w USO1 intracellular protein transport protein
YALI0F16379g	0.40	0.46	similar to uniprot|P54861 Saccharomyces cerevisiae YLL001w DNM1 dynamin-related protein
YALI0F21769g	0.39	0.38	similar to uniprot|P36000 Saccharomyces cerevisiae YKL135c APL2 AP-1 complex subunit beta1-adaptin
YALI0F01210g	0.39	2.00	similar to CA2873|CaAQY1 Candida albicans CaAQY1 putative plasma membrane and water channel protein
YALI0B02992g	0.39	0.22	similar to uniprot|P36147 Saccharomyces cerevisiae YKR065c Hypothetical 22.0 kDa protein
YALI0B04444g	0.37	0.26	similar to uniprot|P38264 Saccharomyces cerevisiae YBR106W Inorganic phosphate transporter PHO88
YALI0D12144g	0.37	0.51	similar to uniprot|Q06142 Saccharomyces cerevisiae YLR347c KAP95 karyopherin-beta singleton
YALI0D08162g	0.36	0.28	highly similar to uniprot|P33723 Neurospora crassa GTP-binding protein ypt1

YALI0C21802g	0.36	0.30	similar to uniprot|P41811 Saccharomyces cerevisiae YGL137w SEC27 coatomer complex beta chain (beta -cop) of secretory pathway vesicles P29.1.f7.1
YALI0B02544g	0.35	0.42	similar to uniprot|Q04182 Saccharomyces cerevisiae YDR406w PDR15 ATP-binding cassette transporter family member
YALI0D00319g	0.35	0.39	some similarities with uniprot|Q08269 Saccharomyces cerevisiae YOL130w ALR1 divalent cation transporter
YALI0E29249g	0.33	0.33	similar to uniprot|P18759 Saccharomyces cerevisiae YBR080C SEC18 Vesicular-fusion protein
YALI0F14575g	0.33	0.35	similar to uniprot|P32337 Saccharomyces cerevisiae YMR308c PSE1 beta karyopherin
YALI0E33649g	0.32	0.22	highly similar to uniprot|P39958 Saccharomyces cerevisiae YER136w GDI1 GDP dissociation inhibitor P2.83. f2.1
YALI0E34672g	0.31	0.21	similar to uniprot|P33303 Saccharomyces cerevisiae YJR095w ACR1 succinate-fumarate transporter P33.2.f7.1
YALI0E05247g	0.30	0.27	similar to uniprot|P32319 Saccharomyces cerevisiae YBL017c PEP1 vacuolar protein sorting/targeting protein
YALI0C10252g	0.29	0.47	similar to uniprot|P43585 Saccharomyces cerevisiae YFL004w VTC2 putative polyphosphate synthetase
YALI0D23463g	0.27	0.18	similar to uniprot|Q96X17 Pichia pastoris Sec7p
YALI0E07425g	0.26	0.28	similar to uniprot|Q12754 Saccharomyces cerevisiae YPL012w hypothetical protein
YALI0D05995g	0.26	0.08	similar to uniprot|P32368 Saccharomyces cerevisiae YKL212w SAC1 Recessive suppressor of secretory defect
YALI0E23243g	0.26	0.11	similar to uniprot|P32867 Saccharomyces cerevisiae YPL232w SSO1 syntaxin-related protein
YALI0E07139g	0.25	0.31	similar to uniprot|P33307 Saccharomyces cerevisiae YGL238w CSE1 importin-beta-like protein
YALI0E23067g	0.24	0.24	uniprot|P41924 Yarrowia lipolytica Ras-like GTP- binding protein
YALI0F31119g	0.23	0.46	similar to uniprot|P32563 Saccharomyces cerevisiae YOR270c VPH1 H+-ATPase V0 domain 95K subunit
YALI0D01133g	0.23	0.34	similar to uniprot|Q99189 Saccharomyces cerevisiae YOR160W mRNA transport regulator MTR10
YALI0E00880g	0.23	0.30	similar to uniprot|O43129 Aspergillus fumigatus MDR2 Multidrug resistance protein 2 possible transmembrane segment
YALI0B20856g	0.22	0.19	similar to KLLA0E23188g Kluyveromyces lactis IPF 2470.1
YALI0F18106g	0.22	0.33	similar to uniprot|Q12344 Saccharomyces cerevisiae YPL249c
YALI0B18282g	0.21	0.88	similar to uniprot|O14367 Schizosaccharomyces pombe Gluconate transport inducer 1
YALI0E02706g	0.19	0.27	similar to uniprot|P38181 Saccharomyces cerevisiae YBL079w NUP170 nuclear pore protein
YALI0F31207g	0.19	0.33	similar to uniprot|P23644 Saccharomyces cerevisiae YMR203w TOM40 forms the hydrophilic channel of the mitochondrial import pore for preproteins

YALI0D07128g	0.17	0.14	similar to uniprot|P36017 Saccharomyces cerevisiae YOR089c VPS21 GTP-binding protein
YALI0E25069g	0.15	0.23	similar to uniprot|P39109 Saccharomyces cerevisiae YDR135c YCF1 Metal resistance protein
YALI0F17996g	0.15	0.11	similar to uniprot|P32568 Saccharomyces cerevisiae YDR011w SNQ2 multidrug resistance protein
YALI0B10021g	0.13	0.23	similar to uniprot|P38861 Saccharomyces cerevisiae YHR170w NMD3 nonsense-mediated mRNA decay protein singleton
YALI0E09471g	0.12	0.17	uniprot|O43108 Yarrowia lipolytica Calcium- transporting ATPase 1
YALI0E21329g	0.12	0.14	similar to uniprot|P36015 Saccharomyces cerevisiae YKL196c YKT6 SNARE protein for Endoplasmic Reticulum-Golgi transport
YALI0E17413g	0.12	0.23	similar to uniprot|P34110 Saccharomyces cerevisiae YJL154c VPS35 protein-sorting protein vacuolar
YALI0F16181g	0.10	0.06	highly similar to uniprot|P32366 Saccharomyces cerevisiae YLR447c VMA6 H+-ATPase V0 domain 36 KD subunit
YALI0D27192g	0.09	0.43	similar to uniprot|P38817 Saccharomyces cerevisiae YHR108W ADP-ribosylation factor binding protein GGA2
YALI0B12980g	0.06	0.07	similar to uniprot|Q96VK4 Emericella nidulans ABC transporter protein
YALI0B05258g	0.04	0.18	similar to uniprot|P47161 Saccharomyces cerevisiae YJR126C Hypothetical 92.0 kDa protein in RPS5-ZMS1 intergenic region

S1E TABLE. Lipid metabolism class

Gene ID	Mean PAI per control replicate	Mean PAI per EgDGAT1-1 replicate	Protein description in Génolevures database
YALI0B19382g	3.04	2.16	similar to uniprot|P43098 Candida albicans Fatty acid synthase subunit alpha (EC 2.3.1.86)
YALI0E34793g	2.86	2.56	highly similar to uniprot|Q8X097 Neurospora crassa Probable ATP citrate lyase subunit 1
YALI0C11407g	2.81	1.75	similar to uniprot|Q00955 Saccharomyces cerevisiae YNR016C Acetyl-CoA carboxylase
YALI0B15059g	2.76	2.15	uniprot|P34229 Yarrowia lipolytica Fatty acid synthase subunit beta (EC 2.3.1.86)
YALI0D24431g	2.71	1.81	similar to uniprot|Q8X096 Neurospora crassa Probable ATP citrate lyase subunit 2
YALI0E15378g	1.86	2.42	uniprot|Q9P4D9 Yarrowia lipolytica Multifunctional beta-oxidation enzyme hydratase-dehydrogenase-epimerase peroxisomal
YALI0B04312g	1.85	2.76	similar to uniprot|O74247 Pichia pastoris and to uniprot|P11986 Saccharomyces cerevisiae YJL153c INO1 myo- inositol-1-phosphate synthase
YALI0D17864g	1.32	1.73	similar to uniprot|P30624 Saccharomyces cerevisiae YOR317w FAA1 long-chain-fatty-acid--CoA ligase
YALI0E01298g	1.11	0.72	uniprot|Q8J0I7 Yarrowia lipolytica Putative sterol carrier protein
YALI0D14850g	1.00	1.00	similar to uniprot|P32463 Saccharomyces cerevisiae YKL192c ACP1 mitochonrial acyl-carrier protein
YALI0F00484g	0.94	0.94	similar to uniprot|Q63060 Rattus norvegicus Glycerol kinase (EC 2.7.1.30)
YALI0E16060g	0.90	0.93	similar to uniprot|P39105 Saccharomyces cerevisiae YMR008c PLB1 phospholipase B (lysophospholipase)
YALI0D06039g	0.87	0.81	similar to uniprot|P22146 Saccharomyces cerevisiae YMR307w GAS1 glycophospholipid-anchored surface glycoprotein
YALI0D01980g	0.67	0.37	similar to uniprot|P40579 Saccharomyces cerevisiae YIR035c similarity to human corticosteroid 11-beta-dehydrogenase
YALI0F23793g	0.67	0.45	similar to uniprot|Q04458 Saccharomyces cerevisiae YMR110c
YALI0A02354g	0.52	0.81	similar to uniprot|Q02201 Saccharomyces cerevisiae YKR003w
YALI0C16797g	0.48	0.25	similar to uniprot|Q96VP9 Glomus intraradices Probable acyl-CoA dehydrogenase
YALI0F30481g	0.48	0.48	similar to uniprot|P54839 Saccharomyces cerevisiae YML126c HMGS 3-hydroxy-3-methylglutaryl coenzyme A synthase
YALI0D04488g	0.46	0.26	uniprot|P45816 Yarrowia lipolytica SEC14 cytosolic factor (Phosphatidylinositol / phosphatidylcholine transfer protein) (PI/PC TP)

YALI0D11330g	0.43	0.43	similar to uniprot|P36060 Saccharomyces cerevisiae YKL150w MCR1 cytochrome-b5 reductase
YALI0E12573g	0.43	0.17	similar to uniprot|Q8AW60 Brachydanio rerio SI:dZ181P14.1
YALI0D06215g	0.40	0.46	similar to uniprot|Q9C251 Neurospora crassa Related to enoyl-CoA-hydratase
YALI0D24750g	0.40	0.49	uniprot|O74936 Yarrowia lipolytica POX3 Acyl-CoA oxidase 3 (EC 1.3.3.6), peroxisomal, specific toward short chain fatty acid
YALI0E16016g	0.36	0.57	similar to uniprot|P38225 Saccharomyces cerevisiae YBR041w FAT1 very long-chain fatty acyl-CoA synthetase singleton
YALI0D18403g	0.33	0.35	similar to uniprot|Q06321 Saccharomyces cerevisiae YLR189c UGT51 sterol glucosyltransferase (UDP-glucose:sterol glucosyltransferase)
YALI0B08536g	0.31	0.45	similar to uniprot|Q04677 Candida tropicalis Acetyl- CoA acetyltransferase IB (EC 2.3.1.9) Peroxisomal acetoacetyl-CoA thiolase Thiolase IB
YALI0C23859g	0.31	0.64	uniprot|O74938 Yarrowia lipolytica POX5 Acyl-CoA oxidase 5 (EC 1.3.3.6), peroxisomal
YALI0D15708g	0.30	0.18	similar to uniprot|P45954 Homo sapiens Acyl-CoA dehydrogenase short/branched chain specific mitochondrial precursor
YALI0C03003g	0.30	0.23	similar to uniprot|P32573 Saccharomyces cerevisiae YNL202w SPS19 peroxisomal 2 4-dienoyl-CoA reductase singleton
YALI0C11693g	0.29	0.31	similar to uniprot|P35844 Saccharomyces cerevisiae YPL145C KES1 protein (Oxysterol-binding protein homolog 4)
YALI0D05291g	0.28	0.44	some similarities with uniprot|P40075 Saccharomyces cerevisiae YER120w SCS2 required for inositol metabolism
YALI0A06787g	0.26	0.24	similar to uniprot|P38286 Saccharomyces cerevisiae YBR159W Hypothetical oxidoreductase in RPB5-CDC28 intergenic region (EC 1.-.-.-)
YALI0F00396g	0.25	0.41	similar to uniprot|Q10740 Saccharomyces cerevisiae YNL045w
YALI0E05753g	0.24	0.24	similar to uniprot|P08524 Saccharomyces cerevisiae YJL167w ERG20 farnesyl-pyrophosphate synthetase
YALI0C00209g	0.21	0.39	similar to uniprot|P32784 Saccharomyces cerevisiae YBL011w SCT1 suppresses a choline-transport mutant
YALI0F10857g	0.20	0.33	uniprot|O74935 Yarrowia lipolytica POX2 Acyl-CoA oxidase 2 (EC 1.3.3.6)
YALI0E28153g	0.18	0.20	similar to uniprot|P37297 Saccharomyces cerevisiae YLR305c STT4 phosphatidylinositol-4-kinase
YALI0F16940g	0.15	0.32	some similarities with uniprot|P35845 Saccharomyces cerevisiae YAR042W SWH1 Protein similar to mammalian oxysterol-binding protein
YALI0E17655g	0.11	0.19	some similarities with uniprot|Q8X0B4 Neurospora crassa Related to hormone-sensitive lipase

YALI0C02805g	0.11	0.18	similar to uniprot|Q9HE38 Neurospora crassa Probable NADPH-dependent beta-ketoacyl reductase (rhlG)
YALI0C03179g	0.10	0.18	similar to uniprot|Q96VQ7 Emericella nidulans Serine palmitoyl CoA transferase subunit LCBA
YALI0E04807g	0.06	0.11	similar to uniprot|P12683 Saccharomyces cerevisiae YML075c HMG1 3-hydroxy-3-methylglutaryl-coenzyme A reductase 1

S1F TABLE. Sugar synthesis and cell wall biogenesis/architecture class

Gene ID	Mean PAI per control replicate	Mean PAI per EgDGAT1-1 replicate	Protein description in Génolevures database
YALI0B21428g	1.41	1.29	highly similar to uniprot|P14742 Saccharomyces cerevisiae YKL104C Glucosamine--fructose-6-phosphate aminotransferase
YALI0D04851g	1.32	1.67	similar to uniprot|Q08193 Saccharomyces cerevisiae YOL030w strongly similar to glycoprotein Gas1P
YALI0D07480g	1.19	0.89	similar to uniprot|P38248 Saccharomyces cerevisiae YBR078w ECM33 involved in cell wall
YALI0E06479g	1.04	1.60	similar to uniprot|P23254 Saccharomyces cerevisiae YPR074c TKL1 transketolase 1 or uniprot|P33315 Saccharomyces cerevisiae YBR117c TKL2 transketolase 2
YALI0D07634g	0.97	0.97	similar to uniprot|P38715 Saccharomyces cerevisiae YHR104w GRE3 aldose reductase
YALI0F18502g	0.95	1.50	similar to uniprot|P23337 Saccharomyces cerevisiae YFR015c GSY1 UDP glucose--starch glucosyltransferase isoform 1 or uniprot|P27472 Saccharomyces cerevisiae YLR258w GSY2 UDP-glucose--starch glucosyltransferase isoform 2
YALI0A03597g	0.91	1.16	similar to uniprot|Q03655 Saccharomyces cerevisiae YMR215W GAS3 protein precursor
YALI0A02310g	0.89	1.11	highly similar to uniprot|P32861 Saccharomyces cerevisiae YKL035w UGP1 UTP--glucose-1-phosphate uridylyltransferase
YALI0E02090g	0.80	0.59	similar to uniprot|P37012 Saccharomyces cerevisiae YMR105c PGM2 phosphoglucomutase major isoform or uniprot|P33401 Saccharomyces cerevisiae YKL127w PGM1 phosphoglucomutase minor isoform
YALI0C23364g	0.80	0.50	similar to uniprot|P31382 Saccharomyces cerevisiae YAL023c PMT2 mannosyltransferase
YALI0F18590g	0.72	1.00	similar to uniprot|P14065 Saccharomyces cerevisiae YOR120w GCY1 galactose-induced protein of aldo/keto reductase or uniprot|Q12458 Saccharomyces cerevisiae YDR368w YPR1 strong similarity to members of the aldo/keto reductase family
YALI0A19074g	0.61	0.82	similar to uniprot|O93808 Saccharomyces cerevisiae YPR184W Oligo-1 4-1 4-glucantransferase / amylo-1 6-glucosidase
YALI0E15125g	0.59	0.51	similar to uniprot|O60064 Schizosaccharomyces pombe Putative mannose-1-phosphate guanyltransferase
YALI0C06490g	0.52	0.63	highly similar to uniprot|O93827 Candida albicans CaPSA1 Mannose-1-phosphate guanyltransferase (EC 2.7.7.13)
YALI0D18964g	0.43	0.50	similar to uniprot|P87218 Candida albicans Sorbitol utilization protein SOU2
YALI0D06281g	0.42	0.12	highly similar to uniprot|Q9HGE2 Trichoderma reesei Mannose phospho-dolichol synthase

YALI0C06798g	0.36	0.58	similar to uniprot|P32775 Saccharomyces cerevisiae YEL011w GLC3 1 4-glucan branching enzyme (glycogen branching enzyme)
YALI0F04169g	0.36	0.80	similar to uniprot|P06738 Saccharomyces cerevisiae YPR160w GPH1 glycogen phosphorylase
YALI0D11176g	0.31	0.48	similar to uniprot|P47169 Saccharomyces cerevisiae YJR137c ECM17 involved in cell wall biogenesis and architecture
YALI0E15081g	0.29	0.14	similar to uniprot|P33775 Saccharomyces cerevisiae YDL095w PMT1 mannosyltransferase
YALI0E21021g	0.25	0.27	uniprot|Q9UVK9 Yarrowia lipolytica 1 3-beta-glucan synthase activity
YALI0E05929g	0.20	0.08	similar to uniprot|P46971 Saccharomyces cerevisiae YJR143c PMT4 dolichyl-phosphate-mannose--protein O-mannosyl transferase
YALI0F14927g	0.18	0.12	similar to uniprot|P53008 Saccharomyces cerevisiae YGL027c CWH41 ER glucosidase I
YALI0D07084g	0.15	0.15	similar to uniprot|Q03161 Saccharomyces cerevisiae YMR099c conserved protein
YALI0D02321g	0.14	0.09	highly similar to uniprot|Q96VU5 Cryptococcus neoformans UDP-glucose dehydrogenase Uxs2p (EC 1.1.1.22)
YALI0B06600g	0.11	0.07	similar to uniprot|P38138 Saccharomyces cerevisiae YBR229c ROT2 glucosidase II catalytic subunit singleton
YALI0A19910g	0.09	0.30	similar to uniprot|P47137 Saccharomyces cerevisiae Probable oxidoreductase YJR096W
YALI0B15510g	0.09	0.15	similar to uniprot|P32623 Saccharomyces cerevisiae YEL040w UTR2 cell wall protein P3.82.f3.1
YALI0B18348g	0.06	0.08	similar to uniprot|P29952 Saccharomyces cerevisiae YER003c PMI40 mannose-6-phosphate isomerase

S1G TABLE. Cellular cycle and cytoskeleton dynamics class

Gene ID	Mean PAI per control replicate	Mean PAI per EgDGAT1-1 replicate	Protein description in Génolevures database
YALI0D08272g	3.96	4.58	uniprot|Q9UVF3 Yarrowia lipolytica Actin
YALI0A19470g	1.45	1.52	similar to uniprot|Q06440 Saccharomyces cerevisiae YLR429w CRN1 a coronin that promotes actin polymerization and crosslinking to microtubules
YALI0B08272g	1.02	1.85	some similarities with uniprot|P08965 Schizosaccharomyces pombe Meiosis protein mei2
YALI0E19360g	0.94	0.54	highly similar to uniprot|P09733 Saccharomyces cerevisiae YML085c TUB1 alpha-1 tubulin or uniprot|P09734 Saccharomyces cerevisiae YML124c TUB3 alpha-3 tubulin
YALI0E00726g	0.78	0.51	highly similar to uniprot|P02557 Saccharomyces cerevisiae YFL037w TUB2 beta-tubulin
YALI0B17622g	0.74	0.89	highly similar to uniprot|P32599 Saccharomyces cerevisiae YDR129c SAC6 actin filament bundling protein fimbrin singleton
YALI0E17325g	0.69	0.83	some similarities with uniprot|P38314 Saccharomyces cerevisiae YBR214w SDS24
YALI0E13486g	0.64	0.76	highly similar to uniprot|O13473 Kluyveromyces lactis Centromere/microtubule binding protein CBF5 and KLLA0D04796g Kluyveromyces lactis IPF 5085.1
YALI0F20856g	0.62	0.57	highly similar to uniprot|Q03048 Saccharomyces cerevisiae YLL050c COF1 cofilin actin binding and severing protein
YALI0B07183g	0.61	0.67	similar to uniprot|P07274 Saccharomyces cerevisiae YOR122c PFY1 profilin
YALI0E02046g	0.58	0.61	similar to uniprot|Q04439 Saccharomyces cerevisiae YMR109w MYO5 myosin I
YALI0E00176g	0.56	0.38	similar to uniprot|P19524 Saccharomyces cerevisiae YOR326w MYO2 myosin heavy chain
YALI0C07502g	0.50	0.55	uniprot|O94097 Yarrowia lipolytica Sla2p cytoskeleton assembly control protein
YALI0D11220g	0.49	0.67	similar to uniprot|P47079 Saccharomyces cerevisiae YJL008C T-complex protein 1 theta subunit (TCP-1-theta) (CCT-theta)
YALI0F01694g	0.46	0.64	some similarities with uniprot|P25623 Saccharomyces cerevisiae YCR030c and DEHA0D03916g Debaryomyces hansenii IPF 10442.1
YALI0F11473g	0.43	0.26	highly similar to uniprot|P39076 Saccharomyces cerevisiae YIL142w CCT2 chaperonin of the TCP1 ring complex
YALI0B10516g	0.42	0.43	similar to uniprot|P17555 Saccharomyces cerevisiae YNL138w SRV2 adenylate cyclase-associated protein 70kDa
YALI0E21692g	0.38	0.10	highly similar to uniprot|P40413 Saccharomyces cerevisiae YJR064w CCT5 T-complex protein 1 epsilon subunit
YALI0D12518g	0.36	0.41	similar to uniprot|Q12018 Saccharomyces cerevisiae

			YDL132w CDC53 controls G1/S transition
YALI0D08536g	0.34	0.77	some similarities with uniprot|P40485 Saccharomyces cerevisiae YIL105c
YALI0D20570g	0.33	0.18	highly similar to uniprot|P39077 Saccharomyces cerevisiae YJL014w CCT3 chaperonin of the TCP1 ring complex cytosolic
YALI0D23595g	0.32	0.08	highly similar to uniprot|Q8J058 Coccidioides immitis Septin 2
YALI0C20735g	0.27	0.15	similar to uniprot|P13517 Saccharomyces cerevisiae YIL034c CAP2 F-actin capping protein beta subunit
YALI0A03025g	0.13	0.16	similar to uniprot|O14241 Schizosaccharomyces pombe Probable ARP2/3 complex 34 kDa subunit (P34-ARC)
YALI0D02805g	0.12	0.15	highly similar to uniprot|P33204 Saccharomyces cerevisiae YKL013c ARC19 subunit of the ARP2/3 complex
YALI0E15620g	0.07	0.06	similar to uniprot|P32908 Saccharomyces cerevisiae YFL008w SMC1 chromosome segregation protein

S1H TABLE. Nucleic acid metabolism class

Gene ID	Mean PAI per control replicate	Mean PAI per EgDGAT1-1 replicate	Protein description in Génolevures database
YALI0E09493g	1.28	1.35	similar to uniprot|P07259 Saccharomyces cerevisiae YJL130c URA2 multifunctional pyrimidine biosynthesis protein
YALI0F21010g	1.18	1.55	uniprot|Q99148 Yarrowia lipolytica Bifunctional purine biosynthetic protein ADE1
YALI0F09229g	1.17	1.23	highly similar to uniprot|P36010 Saccharomyces cerevisiae YKL067w YNK1 nucleoside diphosphate kinase
YALI0D24409g	1.16	1.63	highly similar to uniprot|P38009 Saccharomyces cerevisiae YMR120C Bifunctional purine biosynthesis protein ADE17 [Includes: Phosphoribosylaminoimidazolecarboxamide formyltransferase (EC 2.1.2.3) (AICAR transformylase)
YALI0F30019g	1.03	1.12	similar to uniprot|P38972 Saccharomyces cerevisiae YGR061c ADE6 5 -phosphoribosylformyl glycinamidine synthetase
YALI0A11157g	0.71	0.61	highly similar to uniprot|Q07478 Saccharomyces cerevisiae YDL084w SUB2 Probable ATP-dependent RNA helicase involved in pre-mRNA splicing
YALI0B00704g	0.67	0.71	highly similar to uniprot|P07170 Saccharomyces cerevisiae YDR226W Adenylate kinase cytosolic (EC 2.7.4.3) (ATP-AMP transphosphorylase)
YALI0D21530g	0.63	0.47	similar to CA1245|CaIMH3 Candida albicans IMP dehydrogenase
YALI0F09339g	0.59	0.56	some similarities with uniprot|P15700 Saccharomyces cerevisiae YKL024c URA6 uridine-monophosphate kinase and DEHA0D14762g Debaryomyces hansenii
YALI0E32736g	0.56	0.67	similar to uniprot|P49776| Schizosaccharomyces pombe Bis(5'-nucleosyl)-tetraphosphatase (Ap4A hydrolase)
YALI0E16753g	0.55	0.52	similar to uniprot|P47165 Saccharomyces cerevisiae YJR133w XPT1 xanthine phosphoribosyl transferase
YALI0C23144g	0.48	0.35	similar to uniprot|P22147 Saccharomyces cerevisiae YGL173c KEM1 multifunctional nuclease P2.117.f2.1
YALI0B23188g	0.46	0.61	highly similar to uniprot|Q9P4V1 Candida boidinii Phosphoribosyl-5-aminoimidazole carboxylase
YALI0F23463g	0.44	0.42	similar to uniprot|P47143 Saccharomyces cerevisiae Putative adenosine kinase (EC 2.7.1.20) YJR105w ADO1
YALI0B05368g	0.44	0.71	highly similar to uniprot|P28274 Saccharomyces cerevisiae YBL039C CTP synthase 1 (EC 6.3.4.2) (UTP-- ammonia ligase 1)
YALI0B17402g	0.43	0.49	highly similar to uniprot|Q05911 Saccharomyces cerevisiae YLR359w ADE13 adenylosuccinate lyase
YALI0F31273g	0.31	0.28	highly similar to uniprot|P18562 Saccharomyces cerevisiae YHR128w FUR1 uracil phosphoribosyltransferase

YALI0B15642g	0.26	0.32	highly similar to uniprot|P53131 Saccharomyces cerevisiae YGL120c PRP43 involved in spliceosome disassembly P7.5.f7.1
YALI0E03630g	0.26	0.26	similar to uniprot|Q08162 Saccharomyces cerevisiae YOL021c DIS3 3 -5 exoribonuclease required for 3 end formation of 5.8S rRNA
YALI0E31427g	0.25	0.16	similar to CA3464|CaDBP5 Candida albicans CaDBP5 RNA helicase (by homology)
YALI0E33033g	0.24	0.13	similar to uniprot|Q9C1J4 Pichia pastoris Phosphoribosylamidoimidazole-succinocarboxamide synthase (EC 6.3.2.6) (SAICAR synthetase)
YALI0F21032g	0.24	0.11	uniprot|Q9UUU4 Yarrowia lipolytica ATP-dependent RNA helicase
YALI0E15642g	0.22	0.29	some similarities with uniprot|P22336 Saccharomyces cerevisiae YAR007c RFA1 DNA replication factor A 69 KD subunit
YALI0C21868g	0.15	0.12	highly similar to uniprot|Q03940 Saccharomyces cerevisiae YDR190c RVB1 RUVB-like protein P2.38.f2.1
YALI0F31735g	0.12	0.21	highly similar to uniprot|Q9P839 Candida albicans RNR1 ribonucleotide reductase large subunit

S1I TABLE. Antioxidants and ROS metabolism class

Gene ID	Mean PAI per control replicate	Mean PAI per EgDGAT1-1 replicate	Protein description in Génolevures database
YALI0B15125g	1.57	2.37	highly similar to uniprot|P34760 Saccharomyces cerevisiae YML028w TSA1 thiol-specific antioxidant P3.76. f3.1
YALI0E02310g	1.29	1.19	highly similar to uniprot|P40581 Saccharomyces cerevisiae YIR037w HYR1 glutathione peroxidase
YALI0F08195g	1.26	1.19	similar to uniprot|P34227 Saccharomyces cerevisiae YBL064c
YALI0F20504g	1.10	1.31	similar to uniprot|P00431 Saccharomyces cerevisiae YKR066c CCP1 cytochrome-c peroxidase precursor
YALI0C22000g	1.07	2.33	similar to uniprot|Q04432 Saccharomyces cerevisiae YDR533c
YALI0A19426g	0.85	0.74	similar to uniprot|P34227 Saccharomyces cerevisiae YBL064c strong similarity to thiol-specific antioxidant enzyme
YALI0C21362g	0.83	1.06	similar to uniprot|P39676 Saccharomyces cerevisiae YGR234w YHB1 flavohemoglobin
YALI0D04268g	0.82	0.58	similar to uniprot|P00431 Saccharomyces cerevisiae YKR066c CCP1 cytochrome-c peroxidase precursor
YALI0F30987g	0.49	0.18	similar to uniprot|Q9HDP7 Emericella nidulans Catalase
YALI0D27126g	0.48	0.85	similar to uniprot|P29509 Saccharomyces cerevisiae YDR353W Thioredoxin reductase 1
YALI0E34749g	0.33	1.13	similar to uniprot|P06115 Saccharomyces cerevisiae YGR088w CTT1 catalase T cytosolic
YALI0C16621g	0.30	0.33	similar to uniprot|P00447 Saccharomyces cerevisiae YHR008c SOD2 superoxide dismutase (Mn) precursor mitochondrial (EC 1.15.1.1)

S1J TABLE. Transcription class

Gene ID	Mean PAI per control replicate	Mean PAI per EgDGAT1-1 replicate	Protein description in Génolevures database
YALI0C08635g	0.89	0.82	similar to uniprot|P87216 Schizosaccharomyces pombe VIP1 protein (P53 antigen homolog)
YALI0B12166g	0.71	0.38	uniprot|Q8TG23 Yarrowia lipolytica Putative multi- protein binding factor 1
YALI0F13783g	0.40	0.41	similar to uniprot|P10964 Saccharomyces cerevisiae YOR341w RPA190 DNA-directed RNA polymerase I 190 KD alpha subunit
YALI0D03718g	0.38	0.38	highly similar to uniprot|P08518 Saccharomyces cerevisiae YOR151c RPB2 DNA-directed RNA polymerase II 140 kDa chain
YALI0E09845g	0.30	0.33	similar to uniprot|P22138 Saccharomyces cerevisiae YPR010c RPA135 DNA-directed RNA polymerase I 135 KD subunit
YALI0B07579g	0.28	0.35	some similarities with uniprot|P14922 Saccharomyces cerevisiae YBR112c CYC8 general repressor of transcription singleton
YALI0D26315g	0.26	0.26	uniprot|Q8WZL5 Yarrowia lipolytica Sin3 protein transcription regulatory protein
YALI0B01224g	0.26	0.27	similar to uniprot|P23615 Saccharomyces cerevisiae YGR116w SPT6 transcription elongation protein singleton
YALI0C11561g	0.21	0.23	similar to uniprot|P27692 Saccharomyces cerevisiae YML010W Transcription initiation protein SPT5
YALI0C16566g	0.17	0.26	highly similar to uniprot|P04050 Saccharomyces cerevisiae YDL140c RPO21 DNA-directed RNA polymerase II 215 KD subunit
YALI0C22550g	0.10	0.06	similar to uniprot|P04051 Saccharomyces cerevisiae YOR116c RPO31 DNA-directed RNA polymerase III 160 KD subunit
YALI0A15686g	0.06	0.09	similar to uniprot|P89105 Saccharomyces cerevisiae YOL145c CTR9 required for G1 cyclin expression

S1K TABLE. DNA scaffold

Gene ID	Mean PAI per control replicate	Mean PAI per EgDGAT1-1 replicate	Protein description in Génolevures database
YALI0F25905g	2.22	2.44	highly similar to uniprot|P61830 Saccharomyces cerevisiae YBR010w HHT1 histone H3
YALI0F05962g	1.86	1.71	similar to uniprot|P52910 Saccharomyces cerevisiae YLR153c ACS2 acetyl-coenzyme A synthetase
YALI0C11385g	1.20	1.13	highly similar to uniprot|P02309 Saccharomyces cerevisiae YNL030w HHF2 histone H4 no start
YALI0F02827g	0.89	1.22	similar to uniprot|Q12692 Saccharomyces cerevisiae YOL012c HTZ1 evolutionarily conserved member of the histone H2A F/Z family of histone variants
YALI0E26477g	0.83	0.75	highly similar to uniprot|P04912 Saccharomyces cerevisiae YBL003c HTA2 histone H2A
YALI0E13035g	0.69	0.74	similar to uniprot|Q09184 Schizosaccharomyces pombe SPAC23H4.09 curved DNA-binding protein (42 kDa protein)
YALI0E26455g	0.67	0.92	similar to uniprot|P02293 Saccharomyces cerevisiae YDR224c HTB1 histone H2B
YALI0D14652g	0.44	0.31	similar to uniprot|P32558 Saccharomyces cerevisiae YGL207w SPT16 general chromatin factor
YALI0E02332g	0.34	0.25	similar to DEHA0C09174g Debaryomyces hansenii and uniprot|Q08773 Saccharomyces cerevisiae YOR304w ISW2
YALI0D26103g	0.31	0.51	similar to uniprot|Q00313 Candida albicans DNA topoisomerase I (EC 5.99.1.2)
YALI0F04334g	0.27	0.42	similar to uniprot|P43609 Saccharomyces cerevisiae YFR037c RSC8 subunit of the RSC complex P2.330.f2.1

S1L TABLE. Other proteins class

Gene ID	Mean PAI per control replicate	Mean PAI per EgDGAT1-1 replicate	Protein description in Génolevures database
YALI0F02497g	2.57	2.54	similar to uniprot|Q12428 Saccharomyces cerevisiae YPR002W Hypothetical 57.7 kDa protein in CIT3-HAL1 intergenic region
YALI0E19745g	2.33	1.56	similar to uniprot|Q9HEM5 Neurospora crassa NCU01320.1 hypothetical protein (AL451017) related to microsomal glutathione S-transferase 3
YALI0E01056g	1.96	2.02	uniprot|Q9HFV0 Yarrowia lipolytica C1-THFS protein C1-tetrahydrofolate synthase precursor mitochondrial
YALI0F12155g	1.90	2.06	highly similar to uniprot|P25694 Saccharomyces cerevisiae YDL126c and DEHA0G15994g Debaryomyces hansenii
YALI0C11341g	1.87	3.09	highly similar to uniprot|Q12230 Saccharomyces cerevisiae Hypothetical 38.1 kDa protein YPL004C
YALI0B14377g	1.44	1.85	uniprot|Q876M0 Yarrowia lipolytica 14-3-3 protein promoting filamentous growth
YALI0F11759g	1.42	1.88	highly similar to uniprot|P39954 Saccharomyces cerevisiae YER043c
YALI0F14509g	1.27	0.83	some similarities with uniprot|Q05050 Saccharomyces cerevisiae YMR031c or uniprot|P35736 Saccharomyces cerevisiae YKL050c
YALI0F13541g	1.03	1.46	highly similar to uniprot|P00817 Saccharomyces cerevisiae YBR011c IPP1 inorganic pyrophosphatase cytoplasmic
YALI0F30745g	0.91	1.07	similar to uniprot|Q9HFV0 Yarrowia lipolytica C1- THFS protein and uniprot|P09440 Saccharomyces cerevisiae YBR084w MIS1 C1-tetrahydrofolate synthase
YALI0F09273g	0.90	2.23	similar to uniprot|P43550 Saccharomyces cerevisiae YFL053w DAK2 Dihydroxyacetone kinase 2 or uniprot|P54838 Saccharomyces cerevisiae YML070w DAK1 dihydroxyacetone kinase and DEHA0A03971g Debaryomyces hansenii
YALI0F27555g	0.89	1.44	similar to DEHA0B01694g Debaryomyces hansenii
YALI0E23001g	0.88	0.79	uniprot|Q9HGU7 Yarrowia lipolytica GTP-binding protein
YALI0C24255g	0.81	0.60	similar to uniprot|Q03148 Saccharomyces cerevisiae YMR096w SNZ1 stationary phase protein
YALI0F00682g	0.81	1.22	similar to uniprot|Q04432 Saccharomyces cerevisiae YDR533c hypothetical protein and CAGL0C00275g Candida glabrata and KLLA0D00704g Kluyveromyces lactis or KLLA0D00682g Kluyveromyces lactis
YALI0F31075g	0.80	0.85	uniprot|Q12726 Yarrowia lipolytica Homocitrate synthase
YALI0C21043g	0.75	0.92	similar to uniprot|O14313 Schizosaccharomyces pombe Peroxisomal membrane protein pmp20
YALI0F13717g	0.72	0.74	similar to uniprot|P24276 Saccharomyces cerevisiae YDR293c SSD1 involved in the tolerance to high concentration of Ca2

YALI0D01045g	0.70	0.59	uniprot|Q9HGU7 Yarrowia lipolytica GTP-binding protein Rho1 GTP-binding protein of the rho subfamily of ras-like proteins
YALI0C06171g	0.67	0.94	similar to uniprot|Q884Q9 Pseudomonas syringae Oxidoreductase zinc-binding
YALI0C07414g	0.67	0.50	similar to uniprot|Q9P7B4 Schizosaccharomyces pombe Putative short chain dehydrogenase
YALI0B21340g	0.65	0.64	similar to uniprot|Q9C2C8 Neurospora crassa Probable gamma-adaptin
YALI0D04422g	0.57	0.52	uniprot|Q6L8F1 Yarrowia lipolytica YALI0D04422g YlCPR1 NADPH-cytochrome P450 reductase
YALI0F15873g	0.54	0.68	similar to uniprot|Q06493 Saccharomyces cerevisiae YPR125w MRS7 suppressor of MRS2-1 mutation
YALI0F01496g	0.53	0.53	similar to uniprot|P22803 Saccharomyces cerevisiae YGR209c TRX2 thioredoxin II
YALI0C05665g	0.44	0.33	uniprot|Q876L9 Yarrowia lipolytica 14-3-3 protein Bmh2
YALI0B09471g	0.42	0.15	similar to uniprot|O60121 Schizosaccharomyces pombe Stomatin family protein
YALI0C11803g	0.42	0.42	similar to uniprot|P00817 Saccharomyces cerevisiae YBR011c Inorganic pyrophosphatase
YALI0A09812g	0.41	0.18	similar to uniprot|Q04371 Saccharomyces cerevisiae YMR027w HRT2 high level expression reduced Ty3 transposition
YALI0D27016g	0.40	0.39	some similarities with uniprot|Q9UUJ6 Schizosaccharomyces pombe Nuclear elongation and deformation protein 1
YALI0F00836g	0.39	0.61	similar to uniprot|P31383 Saccharomyces cerevisiae YAL016w TPD3 ser/thr protein phosphatase 2A regulatory chain A
YALI0F30217g	0.38	0.29	highly similar to uniprot|Q8X230 Emericella nidulans VpsA GTPase
YALI0B12078g	0.37	0.24	similar to uniprot|Q02046 Saccharomyces cerevisiae YKR080W Methylenetetrahydrofolate dehydrogenase [NAD+] (EC 1.5.1.15)
YALI0E33473g	0.35	0.36	similar to uniprot|P25618 Saccharomyces cerevisiae YCR017c Hypothetical 107.9 kDa protein in POL4-SRD1 intergenic region putative sensor/transporter protein singleton
YALI0D04983g	0.33	0.33	similar to uniprot|P38626 Saccharomyces cerevisiae YIL043c CBR1 cytochrome-b5 reductase
YALI0B21120g	0.31	0.35	similar to uniprot|P40531 Saccharomyces cerevisiae YIL041W 36.7 kDa protein in CBR5-NOT3 intergenic region
YALI0F05038g	0.31	0.14	similar to uniprot|Q9P5K8 Neurospora crassa Probable 3-methyl-2-oxobutanoate dehydrogenase (Lipoamide)E1 beta chain
YALI0B07359g	0.31	0.25	similar to uniprot|P49954 Saccharomyces cerevisiae YLR351c NIT3 nitrilase P2.240.f2.1
YALI0E33143g	0.31	0.25	highly similar to uniprot|Q12074 Saccharomyces cerevisiae YPR069c SPE3 putrescine aminopropyltransferase

			(spermidine synthase) P2.22.f2.1
YALI0E05643g	0.30	0.33	similar to uniprot|Q9WYD3 Thermotoga maritima TM0297 Oxidoreductase short chain dehydrogenase/reductase
YALI0B06413g	0.22	0.29	similar to uniprot|Q03558 Saccharomyces cerevisiae YHR179w OYE2 NADPH dehydrogenase (old yellow enzyme) isoform 1
YALI0F32021g	0.22	0.19	similar to uniprot|P39952 Saccharomyces cerevisiae YER154w OXA1 cytochrome oxidase biogenesis protein
YALI0D10131g	0.22	0.80	similar to uniprot|P06169 Saccharomyces cerevisiae YLR044c PDC1 pyruvate decarboxylase isozyme 1 P7.9.f5.1
YALI0A05379g	0.18	0.27	similar to uniprot|P37254 Saccharomyces cerevisiae YNR033w ABZ1 para-aminobenzoate synthase
YALI0F11869g	0.18	0.22	similar to uniprot|P36123 Saccharomyces cerevisiae YKR028w
YALI0C16973g	0.16	0.12	similar to DEHA0G11330g Debaryomyces hansenii IPF 4134.1 and YDR346c uniprot|Q05515 Saccharomyces cerevisiae YDR346c
YALI0C20405g	0.15	0.09	similar to uniprot|P10768 Homo sapiens Esterase D (EC 3.1.1.1)
YALI0D00649g	0.14	0.14	similar to uniprot|P32317 Saccharomyces cerevisiae YEL052w AFG1 ATPase family gene
YALI0E03674g	0.13	0.23	similar to uniprot|P43535 Saccharomyces cerevisiae YFR009w GCN20 positive effector of GCN2P
YALI0E18117g	0.13	0.32	similar to uniprot|P39940 Saccharomyces cerevisiae YER125W Ubiquitin--protein ligase RSP5
YALI0E05533g	0.13	0.24	uniprot|P41929 Lysine acetyltransferase
YALI0E31405g	0.13	0.35	similar to ca|CA5025|CaAMI3 Candida albicans CaAMI3 protein required for normal mitochondrial structure (by homology)
YALI0E00154g	0.12	0.07	similar to uniprot|P38903 Saccharomyces cerevisiae YOR014w RTS1 potential regulatory subunit of protein phosphatase 2A possible transmembrane segment
YALI0A20108g	0.11	0.12	highly similar to uniprot|P38795 Saccharomyces cerevisiae YHR074w QNS1 Putative glutamine-dependent NAD(+) synthetase
YALI0F17248g	0.11	0.09	uniprot|Q9UUU1 Yarrowia lipolytica NUCM protein precursor
YALI0F02013g	0.10	0.06	similar to uniprot|O14295 Schizosaccharomyces pombe SPAC9E9.11 Pyridoxal reductase
